# Ear-EEG Forward Models: Improved Head-Models for Ear-EEG

**DOI:** 10.3389/fnins.2019.00943

**Published:** 2019-09-10

**Authors:** Simon L. Kappel, Scott Makeig, Preben Kidmose

**Affiliations:** ^1^Neurotechnology Lab, Department of Engineering, Aarhus University, Aarhus, Denmark; ^2^Department of Electronic and Telecommunication Engineering, University of Moratuwa, Katubedda, Sri Lanka; ^3^Swartz Center for Computational Neuroscience, Institute for Neural Computation, University of California, San Diego, La Jolla, CA, United States

**Keywords:** ear-EEG forward model, head-model, ear-topography, lead field sensitivity, ear-EEG, EEG forward model

## Abstract

Computational models for mapping electrical sources in the brain to potentials on the scalp have been widely explored. However, current models do not describe the external ear anatomy well, and is therefore not suitable for ear-EEG recordings. Here we present an extension to existing computational models, by incorporating an improved description of the external ear anatomy based on 3D scanned impressions of the ears. The result is a method to compute an ear-EEG forward model, which enables mapping of sources in the brain to potentials in the ear. To validate the method, individualized ear-EEG forward models were computed for four subjects, and ear-EEG and scalp EEG were recorded concurrently from the subjects in a study comprising both auditory and visual stimuli. The EEG recordings were analyzed with independent component analysis (ICA) and using the individualized ear-EEG forward models, single dipole fitting was performed for each independent component (IC). A subset of ICs were selected, based on how well they were modeled by a single dipole in the brain volume. The correlation between the topographic IC map and the topographic map predicted by the forward model, was computed for each IC. Generally, the correlation was high in the ear closest to the dipole location, showing that the ear-EEG forward models provided a good model to predict ear potentials. In addition, we demonstrated that the developed forward models can be used to explore the sensitivity to brain sources for different ear-EEG electrode configurations. We consider the proposed method to be an important step forward in the characterization and utilization of ear-EEG.

## 1. Introduction

Electroencephalography (EEG) is a non-invasive method for recording signals from the brain. Ear-EEG is a method in which EEG is measured from electrodes placed in the ear (Kidmose et al., 2013; Bleichner et al., 2015; Mikkelsen et al., 2015). The main advantage of ear-EEG is that it enables discreet and unobtrusive long-term monitoring of EEG in real-life environments (Fiedler et al., 2016; Goverdovsky et al., 2016; Kappel, 2016; Kappel and Kidmose, 2018).

The electrical field in the brain and on the surface of the scalp are related to electrical current from cortical sources through volume conduction. The volume conductor is described by the anatomy of the head, and is typically modeled as four segments of different tissue types: the brain, the cerebrospinal fluid (CSF), the skull, and the scalp. The geometric description of the head anatomy is referred to as the *head model*. As the different tissue types are associated with different electrical conductivity, the volume conductor is an inhomogeneous structure. Although the volume conductor is an inhomogeneous and anatomically complex structure, it is still a linear model (Nunez and Srinivasan, 2006). The transfer function from sources in the brain volume to electrodes on the surface of the head is referred to as the *forward model*. Due to the complex structure, there are in general no feasible closed form solution, and numerical methods are used to compute the transfer functions from equivalent current sources in the brain to electrical potentials on the surface of the head.

One of the main reasons for developing a forward model is to use it in the opposite direction i.e., to calculate the location of brain sources based on a measured surface topography. One approach to source localization is to first perform an independent component analysis (ICA) on the multichannel EEG signal (Makeig et al., 1996), and subsequently find the dipole position and orientation that best explains the topography of the components from the ICA – this method is known as dipole fitting (Scherg, 1990). ICA identifies temporally independent signal sources, called independent components (ICs), as well as linear projections from the sources to the observed signals (measurements). In the case of EEG signals, the linear projections represent how a given source maps to the surface of the head. Many of these linear projections, also called component maps, have shown to be very similar to the pattern generated by a single dipolar current source filtered through a forward model (Delorme et al., 2012), thus motivating the dipole fitting approach to source localization.

The objective and novelty of this paper is to extend existing forward models to include a precise description of the external ear anatomy, including the concha and ear-canal. We will refer to such a model as an *ear-EEG forward model*. Thus, compared to existing forward models, the ear-EEG forward model also describes the transfer function from brain sources to surface potentials of the outer ear.

**Figure 1:**
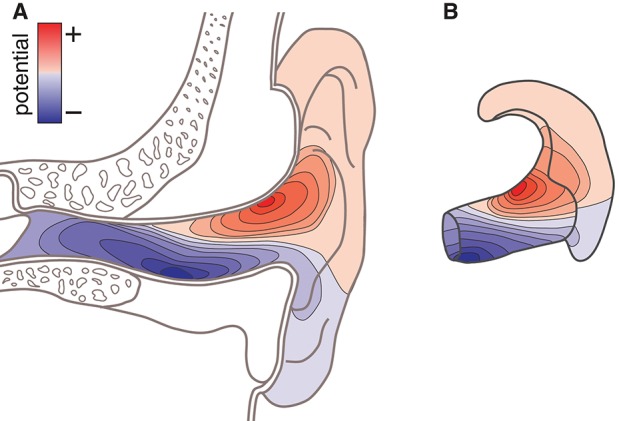
Conceptual sketch showing the surface potential as topographic maps. **(A)** Topographic map on an anatomical cross-section of the outer ear. **(B)** Topographic map shown on an ear-EEG earpiece (ear-topography).

There are several reasons why such a model would be valuable. For example, consider the case in which the locations of the neural sources related to a specific phenomenon is known a priori; or consider that the location of the neural sources can be estimated from conventional high-density scalp EEG recordings. In such cases, the ear-EEG forward model can be used to calculate the corresponding surface potential in the ears, and thereby predict to what extent the phenomenon of interest would be observable from ear-EEG recordings. Moreover, the optimal electrode configurations for measuring a phenomenon of interest can be determined from the surface topography of the potentials in the ear. Figure 1A illustrates the surface topography in the outer ear, and Figure 1B shows the ear-EEG earpiece with the corresponding surface topography, which we will refer to as the *ear-topography*.

Another valuable use of an ear-EEG forward model is to calculate the lead field sensitivity. This provides insight into which brain sources that map to the ear (Malmivuo et al., 1997), and also which regions of the brain that could be stimulated via transcutaneous electrical stimulation.

This paper presents a method for creating ear-EEG forward models by extending existing methods. Ear-EEG forward models were created for four subjects, and evaluated by comparing measured EEG data with data computed with the ear-EEG forward model. Furthermore, to illustrate the spatial coverage of ear-EEG, the lead field sensitivity to brain sources were calculated for both unilateral and bilateral ear-EEG electrode configurations. The study was approved by the regional scientific ethics committee (case no: 1-10-72-46-17).

## 2. Methods

This section is divided into five subsections: section 2.1 describe the methods used to create the individualized ear-EEG forward models, section 2.2 describes the experimental setup, section 2.3 describes the recording paradigms, section 2.4 describes the EEG signal processing, and section 2.5 describes the methods used to evaluate the ear-EEG forward models.

### 2.1. Ear-EEG Forward Models

The method to create ear-EEG forward models is an extension to existing methods for scalp EEG, and incorporates a more detailed description of the external ear anatomy.

**Figure 2:**
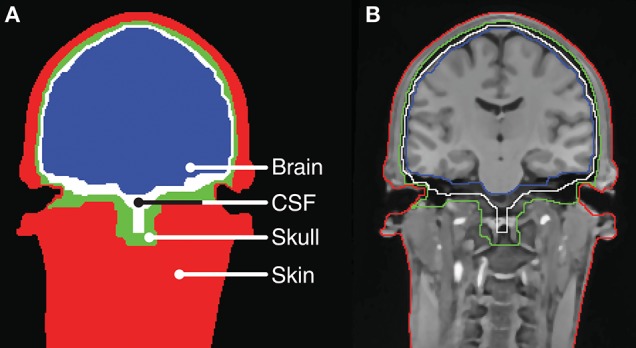
Tissue boundaries for a slice of the head model for Subject C. **(A)** Tissue segments. **(B)** MRI scan with indication of the tissue boundaries.

The head-modeling part relied on already established methods for tissue segmentation and mesh generation from a magnetic resonance imaging (MRI) scan. In this work we used the neuroelectromagnetic forward head modeling toolbox (NFT) for Matlab (Mathworks, MA, USA) developed by Acar and Makeig (2010). The NFT utilize a whole-head T1 weighted MRI scan with a resolution of 1 x 1 x 1 mm to create an individualized head model, and includes tools for segmenting the MRI scan into brain, inner skull, outer skull, and scalp regions. These regions are bounded by triangular tessellated surfaces, also called mesh grids or meshes. To incorporate the ears into the head-model, a detailed geometric representation of the ear anatomy was needed. It was not possible to extract this from the whole-head MRI scan because of poor contrast in the regions of the ears. Conceptually, it is not important how the detailed representation of the ear anatomy is obtained. In principle, MRI scans performed with surface coils close to the ears could be used, or an MRI scanner with a higher field strength could be utilized to increase the contrast in the ear regions. However, in this study the detailed representation of the ear anatomy was obtained from 3D scanned ear impressions of the outer ear, which were already available, as they were used to model individually customized ear-EEG earpieces. This approach further allowed us to determine the positions of the electrodes in the ears by co-registering the earpieces and the scalp electrodes to the head-model, and then calculate the ear electrode positions from the CAD-models of the earpieces. The ear impressions, used in the study, covered the concha and ear-canal part of the outer ear. To incorporate the ears into the head model, 3D scans of the ear impressions were aligned with the scalp mesh and subtracted from the individualized head model. An example of a segmentation, in which the ear impressions were subtracted, is illustrated in Figure 2.

**Figure 3:**
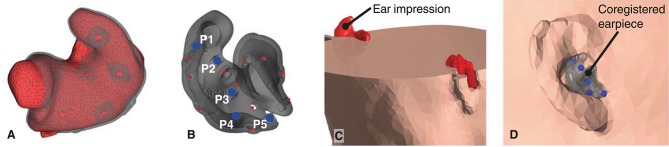
**(A)** 3D scanned ear impression for the right ear with overlay of the ear-EEG earpiece. **(B)** Ear-EEG earpiece for the right ear. Blue dots indicate the fiducials P1-P5 on the earpiece, red dots indicates the location of electrodes. **(C)**. Cross section of the head mesh grid, showing the subtraction of the ear impressions. **(D)** The earpiece placed in the scalp mesh grid of the head-model.

Although conceptually simple, the combination of mesh grids and alignment of coordinate systems requires a number of steps. The following 5 step procedure describes how this was done in the current study:

1. Mesh grids of the outer ear anatomy were generated from 3D scanned ear impressions using a Legato scanner (3Shape, Denmark), and the ear-EEG earpieces were modeled using the computer-aided design tool EarMouldDesigner (3Shape, Denmark). Five fiducial markers (P1-P5) were placed on the 3D model of each earpiece using the Netfabb (Autodesk, CA, USA) software as shown in Figure 3B. The position of the fiducial markers were chosen to span a volume. The distance between the markers were maximized within the limits of positions that could be reached with a digitizer stylus when the earpiece was placed in the ear. An example of an ear impression and the corresponding earpiece are shown in Figure 3A.
2. After manufacturing, the earpieces were placed in the ears of the subject. Locations of the scalp electrodes, the five fiducial markers on each earpiece, and three anatomical landmarks (nasion and the left and right pre-auricular point) were digitized using a NDI Polaris Vicra digitizer system (Northern Digital Inc., Canada).
3. The coordinate systems of the digitized locations and the scalp mesh grid of the head model were aligned as described in Acar and Makeig (2010).
4. To align the 3D scanned ear impressions with the scalp mesh, they were rotated and translated to minimize the least square distance between the digitized positions and the model positions of the fiducial points P1-P5.
5. Finally, the 3D scanned ear impressions were subtracted from the scalp mesh, as shown in Figures 3C,D. To avoid intersections between the head model meshes, the distance between the meshes were forced to a minimum distance of 1 mm. This was done sequentially starting from the scalp mesh to the brain mesh.

**Figure 4:**
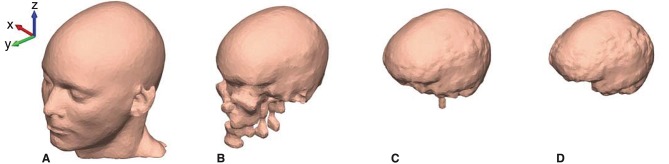
The head model mesh grids for Subject C. **(A)** The scalp, **(B)** The outer skull, **(C)** The inner skull, and **(D)** The brain mesh grid.

The mesh grids of the scalp, outer skull, inner skull, and brain were created with approximately 10,000 faces for each mesh. The resolution of the mesh grids was higher in the ear-region and coarser in the rest of the head model, to obtain a high-quality representation of the details in the concha and ear-canal. An example of the mesh grids are shown in Figure 4.

When calculating the ear-EEG forward model, the tissue between the boundaries were assumed to be isotropic and homogeneous, and the boundary element method (BEM) was used to compute the transfer function from the source space to the measurement space (Akalin-Acar and Gençer, 2004; Hallez et al., 2007). A source space was defined within the volume delimited by the brain mesh in a Cartesian 3D grid with a resolution of 4 mm. The mapping of the electric field from the source space locations to potentials at electrode locations were defined in a lead field matrix (LFM).

The conductivity of the tissues was defined to: σ_*scalp*_ = 0.33 *S*/*m*, σ_*skull* = 0.0132 *S*/*m*, σ_*CSF*_ = 1.79 *S*/*m*, and σ_*brain*_ = 0.33 *S*/*m*. The isolated problem approach (IPA), described by Acar et al., was used for the BEM calculations (Acar and Makeig, 2010).

### 2.2. Experimental Setup

**Figure 5:**
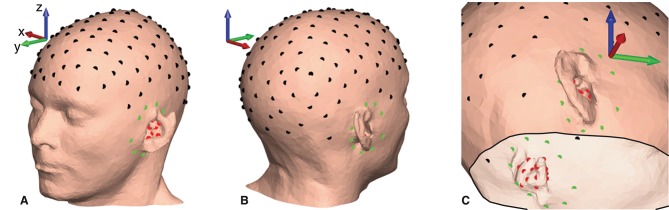
Electrode locations plotted on the scalp mesh grid for subject C. Black dots indicate gelled cap electrodes, green dots indicate dry-contact cEEGrid electrodes, and red dots indicate dry-contact ear-EEG electrodes. **(A,B)** Electrode locations on the scalp surface. **(C)** Electrode locations seen from the inside of the scalp mesh.

EEG was recorded concurrently from electrodes on the scalp (cap), around-the-ears (cEEGrid), and in-the-ears (ear-EEG) using three amplifiers. The electrode locations are shown in Figure 5. The cEEGrid electrodes were included in the measurement setup to increase the spatial coverage of scalp electrodes close to the ears. Thus, the EEG recorded from the cap electrodes and the cEEGrid electrodes will, in the following, collectively be referred to as scalp EEG.

**Figure 6:**
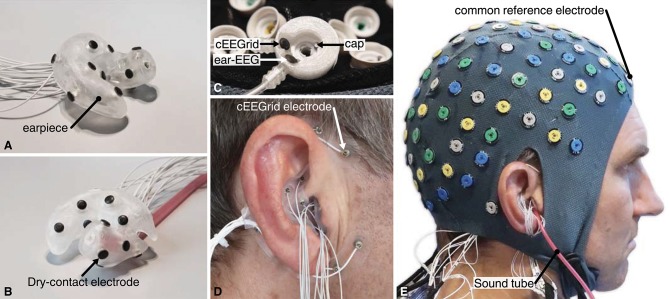
**(A,B)** Example of a right ear-EEG earpiece with 15 dry-contact electrodes on the surface, shown from two different angles. The earpiece shown in **(B)** also have the sound tube mounted. **(C)** The custom-made common reference electrode, mounted at the FPz location in the headcap. **(D)** Earpiece mounted in the ear and cEEGrid around the ear. **(E)** The full experimental setup with cap, cEEGrid, and ear-EEG electrodes. The subject, depictured in **(E)**, has given written informed consent to the publication of the image.

EEG from the cap electrodes was recorded with a TMSi RefA amplifier (TMSi, The Nederlands) at a sampling rate of 2,048 Hz from 128 gelled Ag/AgCl electrodes mounted in a TMSi EEG headcap. EEG from the cEEGrid electrodes was recorded with a first TMSi Mobita amplifier at a sampling rate of 2,000 Hz from 20 dry-contact IrO_2_ electrodes mounted around the ears on the cEEGrid positions (10 around each ear) (Debener et al., 2015; Bertelsen et al., 2019). The ear-EEG was recorded with a second TMSi Mobita amplifier at a sampling rate of 2,000 Hz from 30 dry-contact IrO_2_ electrodes mounted in individualized earpieces (15 in each ear); (Kappel and Kidmose, 2017; Kappel et al., 2019). The ear-EEG earpieces were constructed in non-conductive silicone rubber, and thus did not influence the surface potentials. Prior to insertion of the earpieces, the ears were cleaned with a cotton bud soaked in water. The complete setup is shown in Figure 6.

One channel from each of the 3 amplifiers was connected to a custom-made reference electrode. The reference electrode comprised three independent electrodes, one Ag/AgCl and two IrO_2_, as illustrated in Figure 6C. The custom-made reference electrode was mounted at the FPz location in the headcap and gelled in the same way as the other cap electrodes. Thereby the 3 amplifiers were referenced to practically the same scalp potential. The GND of each of the amplifiers was connected to a conductive bracelet on the right arm. A common trigger signal was connected to all three amplifiers.

Four healthy male subjects with normal hearing and vision, and an average age of 37 years, participated in the study. The recordings were performed in a laboratory, where the subjects were seated in a comfortable chair and instructed to relax. Audio stimuli were presented to the subjects in both ears by insert earphones (3M E-A-RTONE GOLD). The tubes from the earphones were inserted into the earpieces as shown in Figures 6B,E.

Individualized ear-EEG forward models were created for each of the subjects participating in the study. The forward models were based on MRI scans of the subjects' head and impressions of the subjects' ears. The MRI scans were performed with a Siemens 3T MRI scanner (Siemens, Munich, Germany) at a resolution of 1 x 1 x 1 mm and using the Siemens MPRAGE T1 weighed sequence.

### 2.3. Recordings

For the purpose of validating the measurement setup, an auditory steady-state response (ASSR) was recorded prior to the recordings performed for the validation of the ear-EEG forward model. The ASSR stimulus was Gaussian distributed white noise amplitude modulated at 40 Hz. The stimulus was presented binaurally to the subjects with the same stimulus in both ears (mono) and at a sound pressure level of 55 dB relative to the individual hearing threshold. The hearing threshold was estimated using behavioral pure-tone audiometry at 1 kHz according to the guidelines in ISO 8253-1:2010. An 8-Hz trigger, phase locked to the audio stimulus, was recorded together with the EEG, to enable precise alignment of segments in the time domain averaging of the ASSR. The subjects were watching a silent movie without subtitles during the ASSR recording.

For the validation of the ear-EEG forward models, EEG was recorded while the subjects attended an engaging narrative comprising both visual and auditory stimulation. This was chosen to evoke coherent field dynamic patterns (i.e., sources) from several parts of cortex. The narratives were storytelling videos created by the non-profit organization StoryCorps (StoryCorps, NY, USA), and the videos were unknown to the subjects prior to the recording. Specifically we used the videos "‘Q&A'" and "‘The last viewing'" of 3.5 and 3 min duration, respectively. The videos were presented to the subjects on a 40-inch monitor, placed at distance of approximately 100 cm from the subjects' forehead. The audio was presented dichotically (stereo) to the subjects by the insert earphones. A trigger signal was sent to the amplifiers at the beginning and end of each of the videos.

### 2.4. Data Processing

The EEG data processing consisted of:

- Alignment and synchronization of EEG data
- Discarding of channels
- Independent component analysis (ICA)
- Dipole fitting (DipFit)

#### 2.4.1. Alignment and Synchronization of EEG Data

EEG data from the three amplifiers were aligned based on the trigger indicating the beginning of the stimuli. The difference in sampling rate of the amplifiers were determined by calculating the difference in the number of samples between the first and last trigger. Based on this, the data were resampled to a common sampling rate of 500 Hz. Data from all three amplifiers were then referenced to the FPz electrode (the common reference electrode) so that all 178 channels were in the same potential reference system. Finally, the data from the amplifiers were combined into joint data-sets with a common sampling rate and reference. The joint data-sets from the stimulation with the two storytelling videos were cascaded and used for the validation of the ear-EEG forward models.

#### 2.4.2. Discarding

The cEEGrid and ear-EEG electrodes were dry-contact electrodes, which typically have 2–3 orders of magnitude higher electrode impedances compared to wet electrodes (Kappel and Kidmose, 2015). When an electrode has poor skin contact, the impedance may be even higher, which can cause the amplifier's bias current to drive the amplifier into saturation. Therefore, all electrodes which were saturated during a recording were removed from the data-set.

Subsequently, the ASSR was calculated for all possible bipolar configurations of channels within a group, and electrodes were discarded if they were not involved in a configuration with a statistically significant ASSR (*F*-test, *p* < 0.05), as described by Kappel et al. (2019). The groups for discarding were: left ear-EEG (15 electrodes), right ear-EEG (15 electrodes), left scalp (79 electrodes), and right scalp (79 electrodes), with the central scalp (10 electrodes) included in both scalp groups.

#### 2.4.3. Independent Component Analysis (ICA)

The EEG data from the accepted electrodes of the joint data-set including both ear and scalp electrodes, were average referenced, high-pass filtered with a cutoff frequency of 1 Hz, and filtered with 4th order 50, 100, 150, and 200 Hz notch filters, to reduce the harmonics of power line noise. Then, the FPz electrode was removed from the data-set to obtain full rank of the data, and the AMICA (ver. 1.5) ICA algorithm was applied to the data (Palmer et al., 2011).

#### 2.4.4. Dipole Fitting

Single dipole fitting was performed for each independent component (IC). For reasons that will become apparent in the section 2.5, the dipole fitting was based only on the component maps for the scalp electrodes. In practice this was done by removing the rows representing the ear-EEG electrodes in the inverse ICA weight matrix (icawinv in the EEGLab data structure) before the dipole fitting. The dipole fitting was performed using the dipfit algorithm in EEGLab (Delorme and Makeig, 2004) and using the individualized ear-EEG forward models. The dipole fitting algorithm search for the optimal location and orientation of a single dipole to represent the component map.

### 2.5. Evaluation of the Ear-EEG Forward Models

A crucial aspect of mathematical modeling is to evaluate how well a given model describes the system that it is intended to model. A common approach to model evaluation is to define a metric to measure the distance between observed data and modeled data. The difficulty here, however, is that it is not feasible to place a source in the brain volume and then measure the response in the ear. Instead, we estimated brain sources based on scalp EEG recordings, fed these sources through the ear-EEG forward model, and then measured the distance between the component maps from the ICA and the modeled component maps for the ear-EEG electrodes. Thus, to make the estimation of the source location and orientation independent of the ear-EEG recordings, the dipole fitting was based only on the component maps for the scalp electrodes.

In the evaluation of the ear-EEG forward models we used three performance metrics described in the following. The distance between the component maps from the ICA and the modeled component maps were quantified with Pearson's correlation coefficient. C_*m*_ is the Pearson's correlation for the *m*th IC

(1) $$\begin{array}{r} {\text{C}}_{m}=\frac{cov\left(c_{mn},f_{mn}\right)}{\sqrt{var\left(c_{mn}\right)\;\cdot \;var\left(f_{mn}\right)}} \end{array}$$

where *cov*(*c*_*mn*_, *f*_*mn*_) is the covariance between *c*_*mn*_ and *f*_*mn*_ across the electrodes, *n* = 1, …, *N*, and *var*(·) is the variance across the electrodes, *c*_*mn*_ is the coefficients of the *m*th component map, and *f*_*mn*_ is the corresponding coefficients from the ear-EEG forward model, describing the transfer function from the location of the *m*th source (dipole fitted to the mth IC) to the electrode locations. The source locations were quantized to the source space of the ear-EEG forward model. The Pearson's correlation coefficients were computed separately for the left ear-EEG electrodes, the scalp electrodes, and the right ear-EEG electrodes.

To quantify how well the single dipole model described the component maps, we used the residual variance (RV) between the component map from the ICA and the component map modeled by a single dipole. RV_*m*_ denote the RV for the *m*th IC

(2) $$\begin{array}{r} {\text{RV}}_{m}=100\cdot \frac{var\left(c_{mn}-f_{mn}\right)}{var\left(c_{mn}\right)} \end{array}$$

As a measure of the relationship between a measured signal and a given IC, we calculated the percent of variance accounted for (PVAF). PVAF_*m*_ is the total contribution to the measured signals from the *m*th IC, calculated as the mean PVAF over the *N* electrodes

(3) $$\begin{array}{r} {\text{PVAF}}_{m}=100-100\cdot \frac{1}{N}\overset{N}{\underset{n=1}{\sum }}\frac{var\left(x_{n}\left(t\right)-y_{mn}\left(t\right)\right)}{var\left(x_{n}\left(t\right)\right)} \end{array}$$

where *var*(·) is the variance over time, *x*_*n*_(*t*) is the measured signal from electrode *n*, and *y*_*mn*_(*t*) is the projection of the *m*th IC to the *n*th electrode computed as

(4) $$\begin{array}{r} y_{nm}\left(t\right)={\left({\text{W}}^{-1}\right)}_{nm}\cdot u_{m}\left(t\right) \end{array}$$

where *u*_*m*_(*t*) is the *m*th IC, and ${\left({\text{W}}^{-1}\right)}_{nm}$ is the (*n, m*)th element of the inverse ICA mixing matrix **W**^−1^ (corresponding to icawinv in the EEGLab data structure).

## 3. Results and Discussion

### 3.1. Validation of Ear-EEG Forward Models

Each of the head model mesh grids, defining the tissue boundaries, was visually inspected to verify that the segmentation and subtraction of the ear impressions were realistic when compared to the MRI scan. Based on the individual head models, ear-EEG forward models were created for each of the subjects. 3D figures of the head model mesh grids are available in the Supplementary Material for all four subjects.

In the preprocessing of the EEG recordings an average of 6 % of the 30 ear-EEG electrodes, 13 % of the 20 cEEGrid electrodes, and 1 % of the 128 cap electrodes were discarded according to the discard criteria described in section 2.4.

After preprocessing and ICA, single dipole fitting was performed for all ICs. One of the main challenges and criticisms related to dipole fitting of ICs, is to what extent it is appropriate to approximate brain sources with dipole sources. Indeed, it is clear, from practical experiences, that some ICs are better modeled by a single dipole source than others. Thus, the ear-EEG forward models were evaluated based on 12 ICs from each subject, selected according to the following predefined criteria: localized within the brain volume and best modeled by a single dipole. Specifically, among the sources located within the brain volume, we selected the 12 ICs with the lowest residual variance.

For the validation of the ear-EEG forward models, each component map from the ICA were compared with the corresponding component map modeled with the ear-EEG forward model. For the modeling, each of the source locations, determined by dipole fitting, was quantized to the closest source space location of the forward model. Each of the modeled component maps represented the projection from the quantized source location to the electrode locations. The average distance between the source locations, determined by dipole fitting, and the quantized source locations was 3.8 mm (s.d. 5.5 mm) across the 48 selected ICs, and 2.1 mm (s.d. 1.6 mm) across the subset of ICs in **Figure 8**.

**Figure 7:**
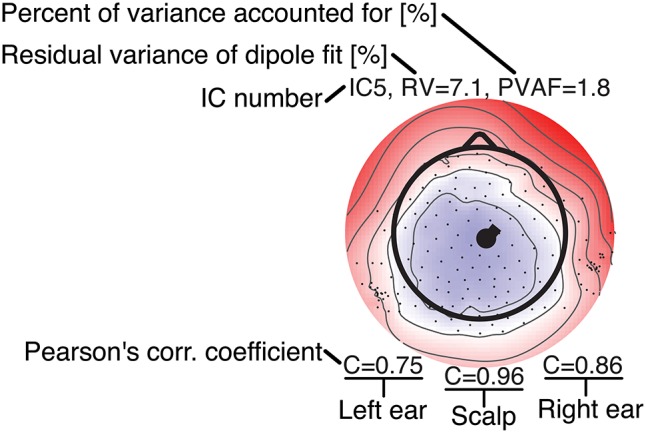
Legend for the component maps shown in Figure 8.

**Figure 8:**
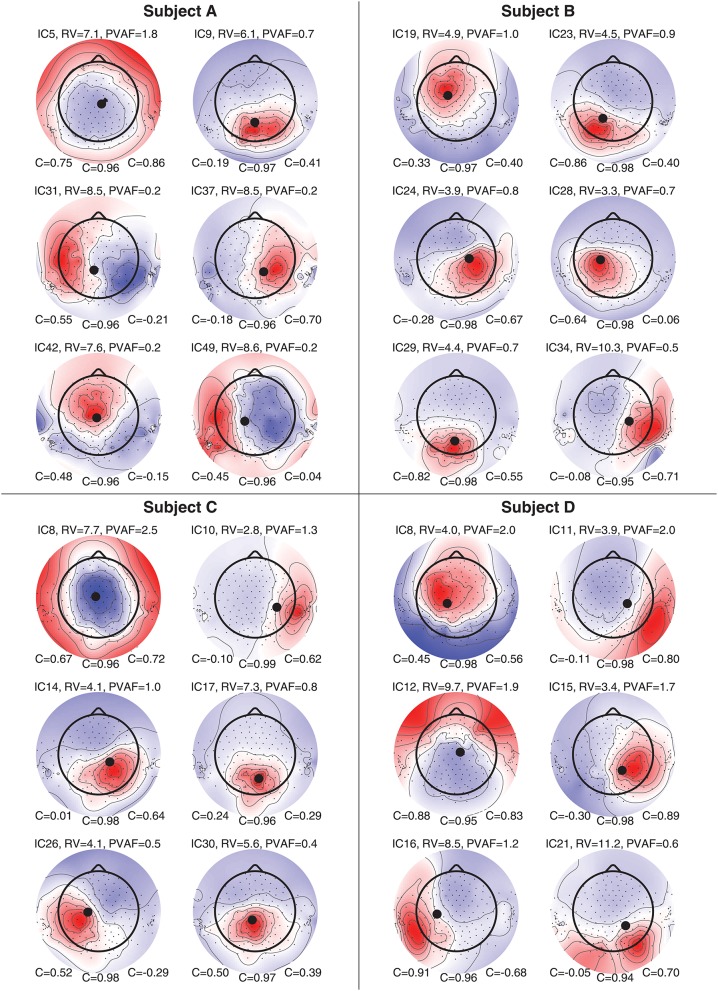
Component maps for 6 ICs from each subject. Above each component map is shown the IC number, the residual variance (RV), and percent of variance accounted (PVAF). Below each component map is given the Pearson's correlation coefficient for the left ear, the scalp, and the right ear, respectively.

**Figure 8** shows component maps for 6 of the 12 ICs for each subject. Above each component map is given the IC number, residual variance (RV) of the dipole fit, and the percent of data variance accounted for (PVAF) by the component. The Pearson's correlation coefficient for the left ear-EEG electrodes, the scalp electrodes, and the right ear-EEG electrodes are given below the component map, as shown in Figure 7. Component maps of all 12 ICs are available in the Supplementary Material for all four subjects. In the following, when referring to a specific IC, we use the notation “ICxS” for IC number “x” from subject “S” ∈ {A, B, C, D}.

From visual inspections of the component maps in Figure 8, it was observed that the topographies in general were smooth across the scalp, and the smooth patterns extended to the regions around and in the ears. This suggests that the component maps for electrodes on the scalp and in the ears were related to the same underlying neural sources. The RV provides a quantitative measure of how well the dipole model fits the component map, as defined by Equation (2). A RV of 0% corresponds to a perfect match between the component map from the ICA and the component map modeled by a single dipole, whereas 100% corresponds to a dipole model in which the variance of the differences between the maps are as large as the total variance of the component map.

The mean RV across the 24 component maps, shown in Figure 8, was 6.3%, which indicates a very good source modeling. Note that the dipole fitting was based only on the component maps for the scalp electrodes, and that each component map was modeled by a single dipole (Delorme et al., 2012). Despite this simple approach, a large majority of the ICs were modeled very well with the single dipole model. A few ICs (see e.g., scalp maps for IC9A and IC12D) would probably have benefited from a more advanced source model, such as a composite model consisting of two symmetrically positioned bilateral dipoles (Piazza et al., 2016). However, to be consistent, and to avoid any manual choices in the source modeling, we choose to limit the model to a single dipole for all ICs.

Pearson's correlation coefficients were calculated between the component maps from the ICA and the modeled component maps, for the left ear-EEG electrodes, the scalp electrodes (i.e., electrodes in the cap and cEEGrid), and the right ear-EEG electrodes separately, as detailed in Equation (1). The mean value of the correlations across the 24 components maps shown in Figure 8 were 0.34, 0.97, and 0.41, for the left ear, the scalp, and the right ear, respectively. The mean correlation for the scalp electrodes indicated a very good match between the component maps from the ICA and the modeled component maps, whereas the correlations for the ear-EEG electrodes were significantly lower. There are likely several reasons for the relatively large deviations between the scalp and ear correlations. One aspect was that the dipole fitting was based only on data from the scalp electrodes. Another aspect could be that the coefficients in the inverse mixing matrix of the ICA decomposition were dominated by noise when a source had a weak mapping to the ear, resulting in an unreliable component map for the ear-EEG electrodes. Nevertheless, mean correlations of 0.34 and 0.41 for the left and right ear, respectively, showed that the ear-EEG forward models generally provided a reasonably good estimate of the ear-topographies (i.e., component maps for the ear-EEG electrodes) for the selected ICs.

**Figure 9:**
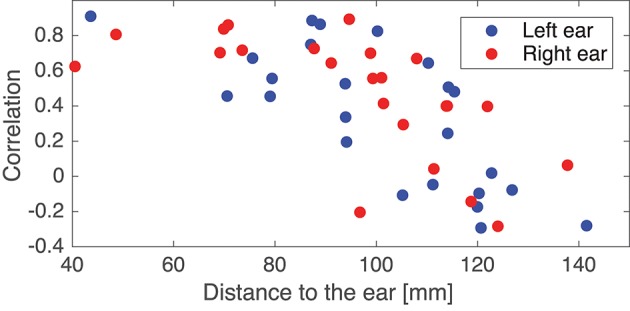
Scatter plot showing the relationship between the Pearson's correlation coefficient and the distance from the source to the left or right ear. The location of an ear was calculated as the average of the ear electrode locations in that ear. The plot is based on the ICs in Figure 8.

In the following some of the results shown in Figure 8 will be discussed in more detail. As a first example, consider the set {IC5A, IC29B, IC8C, IC12D}; these components were characterized by relatively high correlations in both ears (mean correlations of 0.78 and 0.74 for the left and right ear, respectively), and in all four cases the source location was estimated to be quite close to the mid-sagital plane. Even though these sources were relatively far away from the ears, there were still a good match between the ear-topographies from the ICA and the ear-topographies modeled with the ear-EEG forward models. As a second example, consider the set {IC37A, IC34B, IC10C, IC15D}; these components were characterized by low correlations in the left ear and high correlations in the right ear (mean correlations of –0.16 and 0.73 for the left and right ear, respectively). The source location was in these cases estimated to be in the right brain hemisphere. To further investigate this apparent dependency of the distance to the source, Figure 9 shows the relationship between the ear correlation and the distance to the ear for the ICs in Figure 8. The figure shows that the correlation was higher when the source location was close to the ear as compared to further away from the ear. Generally, the correlation was lower for source locations more than 100 mm away from the ear, and we speculate that this could be related to a weak mapping of the source to the ear. As mentioned above, a weak mapping causes uncertain estimates of the coefficients in the inverse mixing matrix, and thereby an unreliable ear-topography.

**Figure 10:**
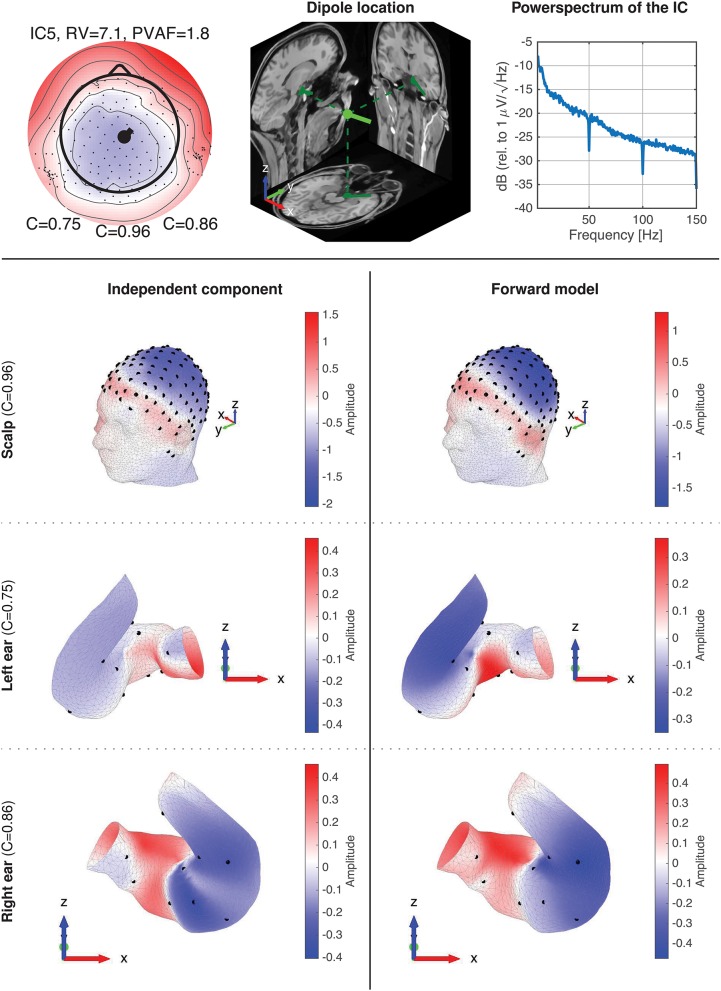
Details of IC5A with corresponding forward model data. The first row shows the component map, dipole location, and power spectrum for the IC. The dipole location shows the dipole location and orientation determined by single dipole fitting of the IC. The 2-4th rows show topographic 3D maps for the scalp, the left ear, and the right ear, respectively. The left column (row 2–4) is the topographic maps (i.e., component maps) for the IC, and the right column is the corresponding topographic maps modeled with the ear-EEG forward model for the subject.

Figure 10 shows data for IC5A. The top row shows the component map, the dipole location, and the power spectrum of the IC data. The dipole location is the location and orientation determined by single dipole fitting of the IC. The power spectrum for the IC is typical for an EEG source, and is representative of the power spectra available in the Supplementary Material. Rows 2–4 shows topographic maps for the scalp, the left ear, and the right ear, respectively. The topographies for the ear data, referred to as *ear-topographies*, were plotted on the mesh grids of the 3D scanned ear impressions. The topographic maps in the left column show the coefficients of the component map for the IC, and the maps in the right column show the coefficients of the corresponding component map modeled with the ear-EEG forward model for the subject. The Pearson's correlation coefficient (C) reflects the correlation between the topographic maps.

A visual inspection of the 3D scalp topographies in Figure 10 revealed a high similarity between the IC and forward model topographies, which were reflected in a high correlation and a low RV. Also for the ears, a high similarity between the ear-topographies were observed, and the corresponding correlations were high for both ears (0.75 and 0.86 for the left and right ear, respectively).

The 24 component maps shown in Figure 8 represents dipoles scattered over the complete brain volume. A consequence of the underlying dipole model is that component maps with a low RV have smooth topographic patterns. However, the patterns of the component maps varies widely depending on the dipole location and orientation. Not surprisingly, ear-topographies share the same characteristics; this can be seen in Figure 10 and is further substantiated by visual inspections of the 96 ear-topographies shown in the Supplementary Material. Therefore, in the same way as for scalp EEG, it is meaningful to have a reasonable high spatial sampling of the surface potential in the ear, to exploit the information mapped from cortical sources to the ear-EEG. This is the reasoning behind high-density ear-EEG recording as proposed in Kappel and Kidmose (2017).

A very large base of scientific studies have mapped different functions to different locations in the brain. The ear-EEG forward model provides a way to calculate how these brain sources maps to the ear. Thereby it is possible to quantify to what extent such sources would be observable from ear-EEG and to optimize the ear-EEG electrode configuration. For example, consider a brain source corresponding to the ear-topographies shown in Figure 10; for these particular ear-topographies, the highest sensitivity would be obtained with an electrode located in the ear-canal relative to an electrode in the upper concha region, corresponding to electrode locations at the highest (red) and lowest (blue) potential, respectively.

Previous studies have mapped the electric field from brain sources to potentials in the ear, based on an idealized two-dimensional model with only one circular shell (Kidmose et al., 2013) and a more advanced 3-layer anatomical model (Goverdovsky et al., 2017). However, none of these studies were based on individualized forward models, and no validation of the models was performed with measured ear-EEG data. Bleichner et al. (2015) emphasized the need for advanced and individual forward models based on digitized ear-EEG electrode locations, to investigate the sensitivity of the ear-EEG to different brain processes. The current study is a significant improvement of previously presented methods, and enables mapping of arbitrary brain source locations and orientations to potentials in the ears.

The proposed method for creating ear-EEG forward models is an extension to forward models for scalp EEG. Therefore, in addition to the fine resolution of the outer ear anatomy, it also includes the scalp anatomy. Thereby, an ear-EEG forward model can be used in the analysis of joint scalp EEG and ear-EEG recordings. It is therefore likely, yet only a speculation, that ear-EEG in combination with scalp EEG can be used to improve source localization. This is likely most relevant for sources in the temporal and insula region of the brain and in the brain stem.

### 3.2. Lead Field Sensitivity

**Figure 11:**
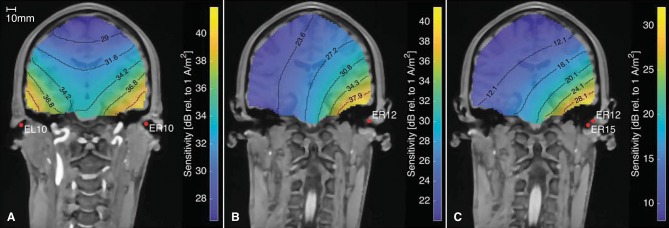
The sensitivity distribution for different electrode configurations, based on an ear-EEG forward model for Subject C. **(A)** Between-ears electrode configuration, **(B)** Ear electrode to an infinite reference, **(C)** Within-ear electrode configuration.

In addition to determining the ear-topographies for a given neural source, the ear-EEG forward models can be used the opposite way around to calculate the lead field sensitivity for different electrode configurations, as described by Malmivuo et al. (1997). Figure 11 shows the sensitivity for three different electrode configurations. The figure is based on an ear-EEG forward model for subject C with a source space resolution of 2 mm. Each plot shows the sensitivity for dipole locations within the brain, and can be used to determine the brain areas from which it might be possible to measure an EEG signal with the specified electrode configuration. According to the reciprocity theorem, described by Nunez and Srinivasan (2006), the orientation of the current density vector, is the dipole orientation which results in the largest potential difference between the electrodes. Thus, the sensitivities given in Figure 11 is for the dipole orientation with the highest sensitivity at the particular location and for the particular electrode configuration.

Figure 11A shows the sensitivity distribution for a between-ears configuration with a high sensitivity close to both ears. Figures 11B,C show the sensitivity distributions for a single electrode, with a reference located in infinity and for a within-ear configuration, respectively. The distance between the contour lines for the within-ear configuration is 4 dB, whereas for the between-ears configuration it is only 2.6 dB. Thus, the sensitivity decreases faster as a function of distance for the within-ear configuration compared to the between-ears configuration. This is in agreement with our intuition from classical physics, where the electrical potential from a dipole scales approximately proportional to the dipole moment, and inverse proportional to the squared distance from the dipole (Nunez and Srinivasan, 2006). Comparing Figures 11A,C, the amplitudes are in general 10–20 dB higher for the between-ears configuration as compared to the within-ear configuration. This is in accordance with many previous studies, in which the amplitudes of within-ear recordings were 10–20 dB lower as compared to scalp referenced ear recordings (see e.g., Kidmose et al., 2013; Mikkelsen et al., 2015; Christensen et al., 2018; Kappel et al., 2019).

**Figure 12:**
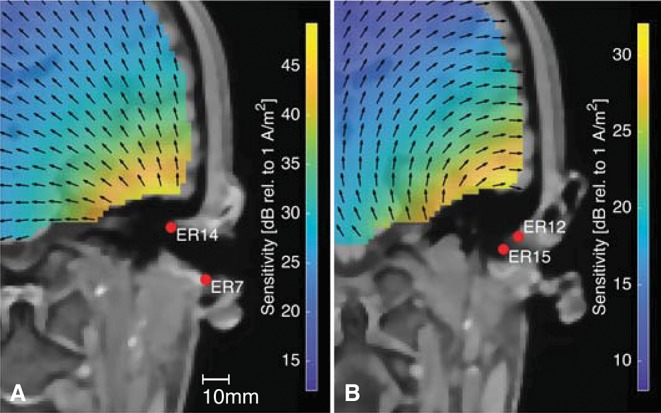
The sensitivity distribution for two different within-ear electrode configurations, with the black arrows showing the orientation of the current density vector. **(A)** The ER14-ER7 electrode configuration, **(B)** The ER12-ER15 electrode configuration.

Figure 12 shows the sensitivity close to the right ear for two different within-ear electrode configurations. Figure 12 is based on the same forward model as Figure 12. The arrows indicate the orientation of the current density vector, corresponding to the dipole orientation of maximum sensitivity as described above. The two electrode configurations are almost perpendicular to each other, and it is noticed that the direction of the current density vectors shown in Figures 12A,B are also almost perpendicular. Any source perpendicular to the current density vector will have zero sensitivity. This provides an intuitive insight into which sources can be recorded from the ear-EEG electrodes, and the importance of the electrode configuration. A consequence of this was discussed in a previous study of individually optimized electrode configurations for measuring the ASSR with ear-EEG (Kappel et al., 2016).

In conventional scalp EEG it is only possible to place electrodes on a surface which, locally, is more or less parallel to the surface of the brain volume. However, this plane limitation can be compensated by placing several electrodes along the scalp surface, and thereby different electrode configurations will be sensitive to different source orientations. In contrast, ear-EEG is inherently limited to a lower spatial coverage, but the concave shape of the outer ear, makes it possible to place electrodes in the ear so that they span a volume. Thereby, sensitivity to different source orientations can be obtained, as illustrated in Figure 12.

## 4. Conclusion

A novel method to create ear-EEG forward models was developed. The method is an extension to existing forward models for scalp EEG, and incorporates a more detailed description of the external ear anatomy. Individualized ear-EEG forward models were created for 4 subjects based on whole head MRI scans and 3D scanned ear impressions. The same 4 subjects participated in a combined ear-EEG and scalp EEG study comprising both auditory and visual stimuli. The EEG recordings were analyzed with ICA. The scalp-part of the component maps were used for single dipole fitting, and the resulting dipole locations were quantized to the source space of the forward models. The ability of an ear-EEG forward model to estimate potentials in the ears, was validated by comparing the ear-part of the component maps from the ICA and the corresponding dipoles projected through the ear-EEG forward model to the ear.

When a component map could be fitted well with a single dipole in the brain volume, the correlation between the component from the ICA and the modeled component map was generally high for the ear-EEG electrodes located in the ear closest to the dipole location. This shows that ear-EEG forward models based on the proposed method, provides a good model to predict potentials in the ears for a given neural source location and orientation. We also demonstrated that ear-EEG forward models can be used to calculate the lead field sensitivity for source locations in the brain. This is an important tool to explore how different ear-EEG electrode configurations are sensitive to different source locations and orientations. Finally, we envision that ear-EEG forward models can be used to improve the precision of source localization for recordings performed with ear-EEG and scalp EEG electrodes simultaneously.

To conclude, we consider the method of creating ear-EEG forward models to be an important step toward the characterization and utilization of the ear-EEG method.

## Data Availability

The dataset for this manuscript are not publicly available because the authors are currently working with the dataset, in relation to other research hypotheses. The dataset will be made available when the current work is finished. Requests to access the dataset should be directed to PK, pki@eng.au.dk.

## Ethics Statement

This study was carried out in accordance with the recommendations of the regional scientific ethics committee with written informed consent from all subjects. All subjects gave written informed consent in accordance with the Declaration of Helsinki. The protocol was approved by the Videnskabsetiske Komitéer for Region Midtjylland, Denmark (case no: 1-10-72-46-17).

## Author Contributions

PK and SK devised the method and wrote the paper. SK implemented the method and experiment, performed the data acquisition, and the data processing with supervision from PK and SM. All authors read and approved the final manuscript.

### Conflict of Interest Statement

The authors declare that the research was conducted in the absence of any commercial or financial relationships that could be construed as a potential conflict of interest. The reviewer YX declared a shared affiliation, with no collaboration, with one of the authors, SM, to the handling editor at time of review.

## Supplementary Material

The Supplementary Material contains figures showing the head model mesh grids for all subjects, component maps of all 12 selected ICs from each subject, and detailed figures comparing the measured and modeled data for each of the selected ICs.
