# Supplementary material for: Ear-EEG Forward Models: Improved Head-Models for Ear-EEG
Source: Front Neurosci. 2019 Sep 10;13:943. doi: 10.3389/fnins.2019.00943 (PMC6747017; doi:10.3389/fnins.2019.00943)

frontiers

Supplementary Material

Ear-EEG Forward Models: Improved Head-Models for Ear-EEG

This document contains additional figures to describe the head models and validation of the ear-EEG forward models. For each subject, the following figures are available:

1. Boundary mesh grids of the head model.
2. Component maps for the 12 independent components (ICs), which were selected for the evaluation of the ear-EEG forward models.
    - Among the sources located within the brain volume, we selected the 12 ICs with lowest residual variance.
3. Detailed figures for each of the 12 components.
    - Refer to Figure 10 in the main manuscript for a description of the individual plots in the figures.

Figures S2, S4, S6, and S8 show the boundary mesh grids.
Figures S3, S5, S7, and S9 show the component maps. Above each component map is given the IC number, residual variance (RV) of the dipole fit, and the percent of data variance accounted for (PVAF) by the component. The Pearson's correlation coefficients for the left ear-EEG electrodes, scalp electrodes, and right ear-EEG electrodes are given below the component map, as shown in Figure S1.
All 3D figures are embedded 3D plots, which can be rotated when the document is opened in Adobe Acrobat Reader.

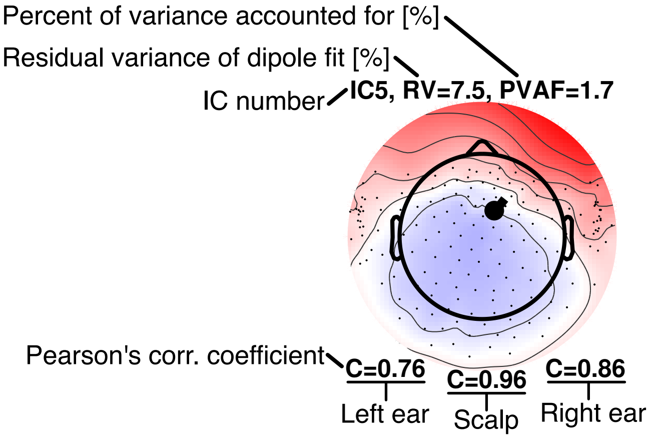

Figure S1. Legend for the component maps in Figures S3, S5, S7, and S9.

CONTENTS

1 Subject A 2
2 Subject B 15
3 Subject C 28
4 Subject D 41

1 SUBJECT A

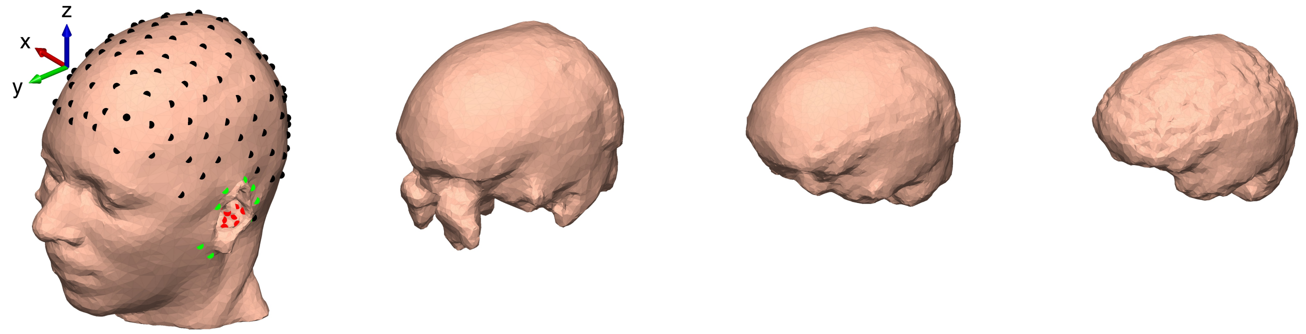

(a) Scalp (b) Outer skull (c) Inner skull (d) Brain

Figure S2. Headmodel mesh grids.

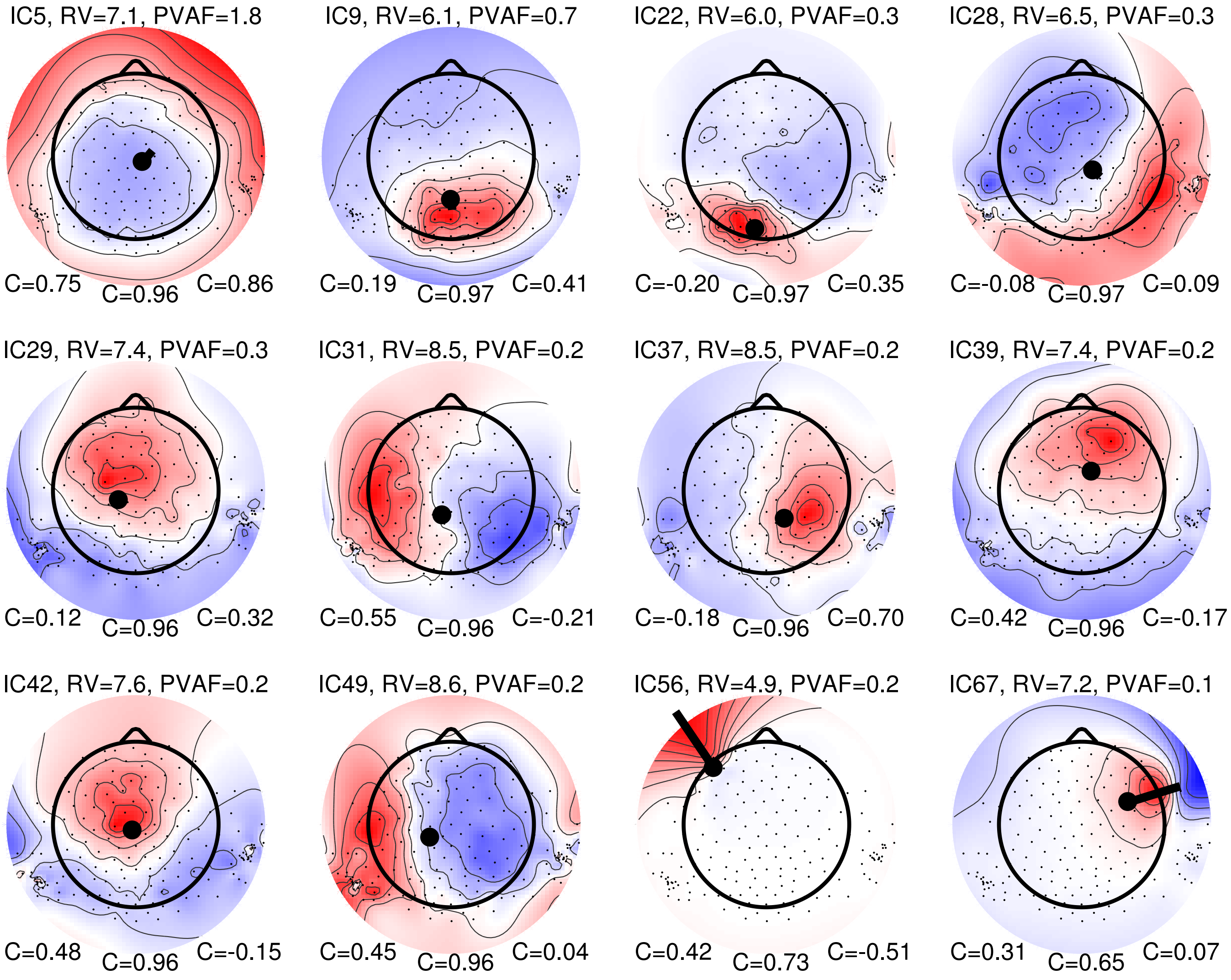

Figure S3. Topographic plots of the 12 independent components with the lowest residual variance.

Subject A - IC 5

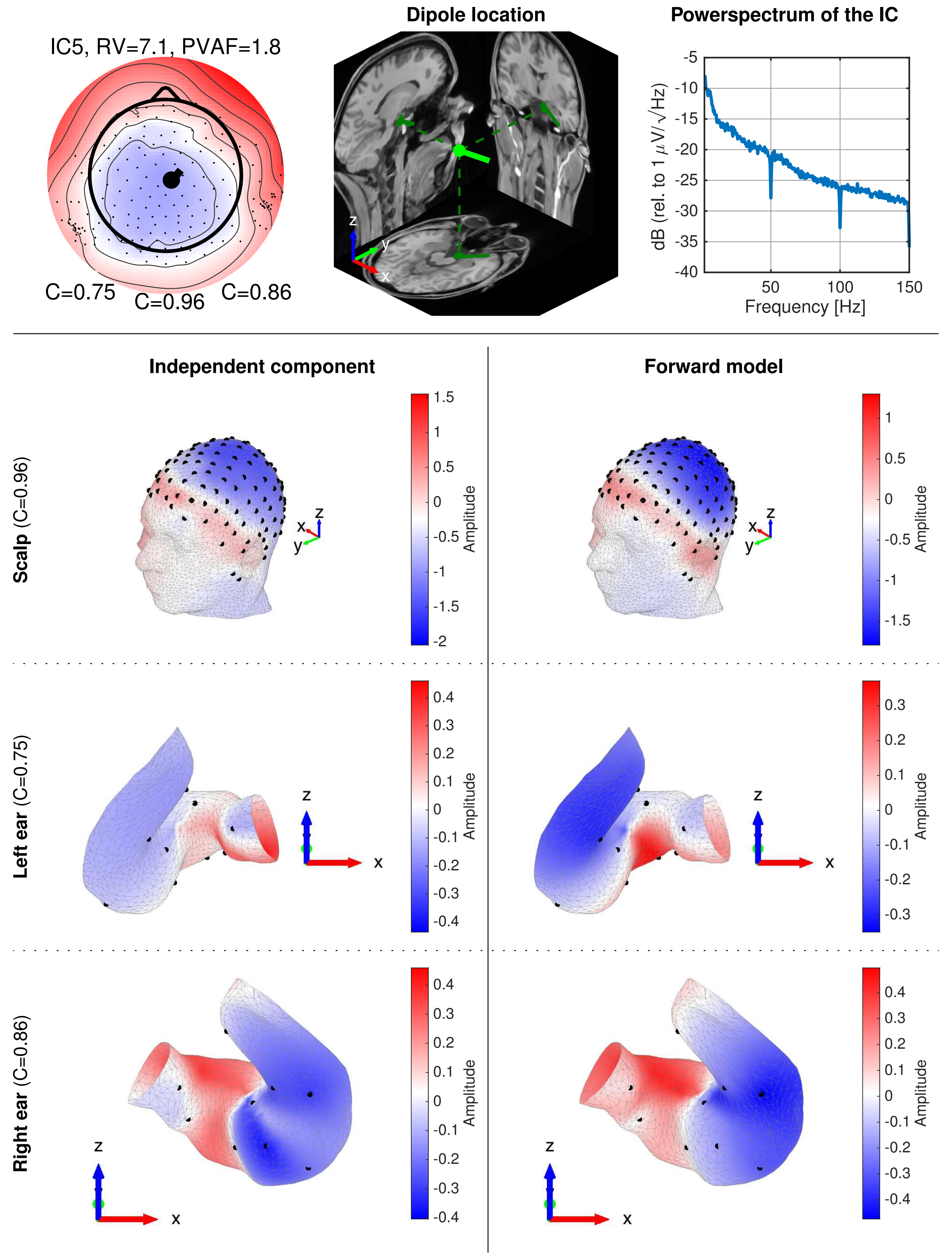

Subject A - IC 9

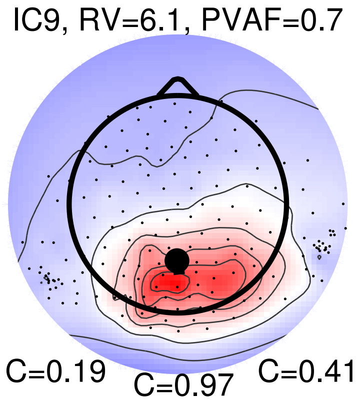

Dipole location

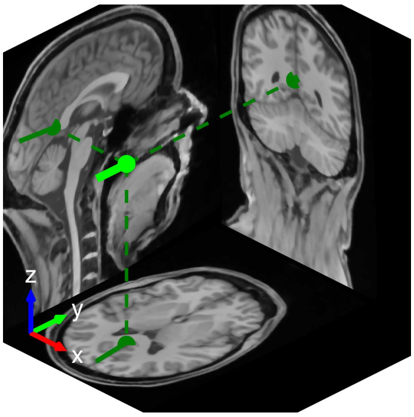

Powerspectrum of the IC

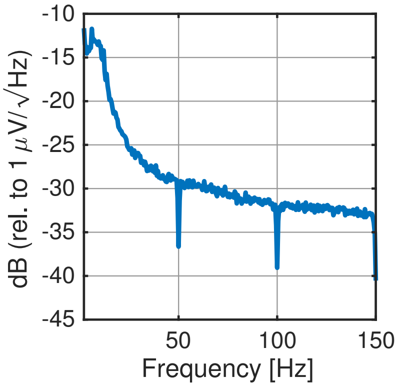

Independent component

Forward model

Scalp (C=0.97)

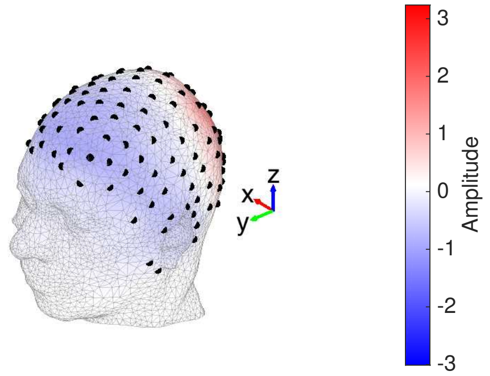

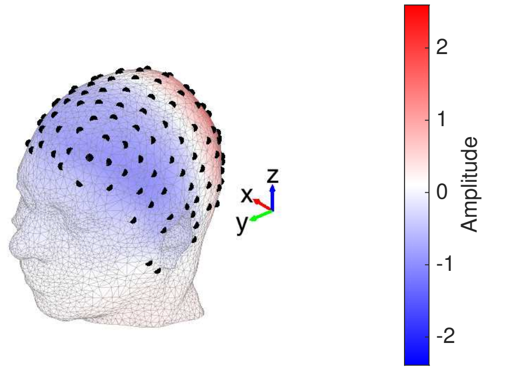

Left ear (C=0.19)

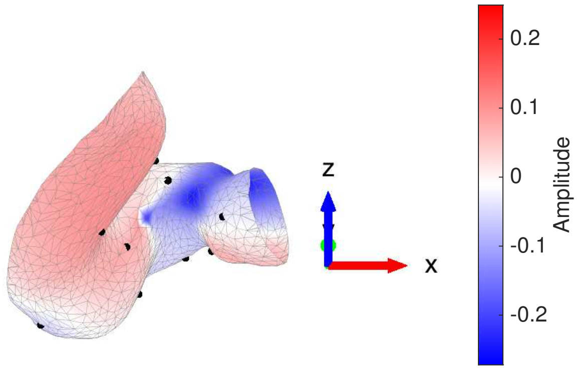

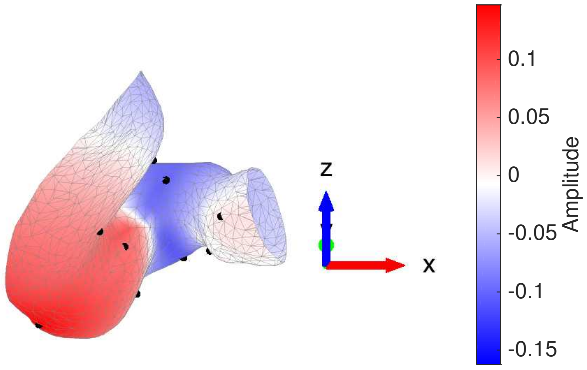

Right ear (C=0.41)

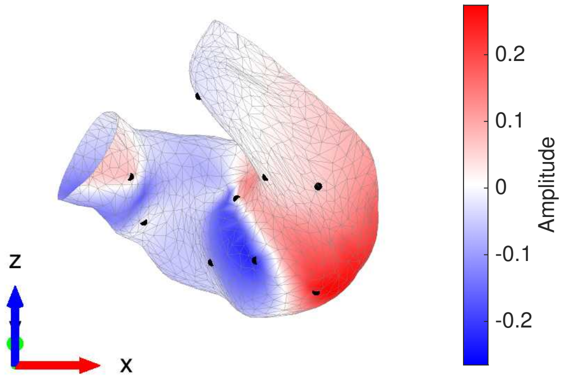

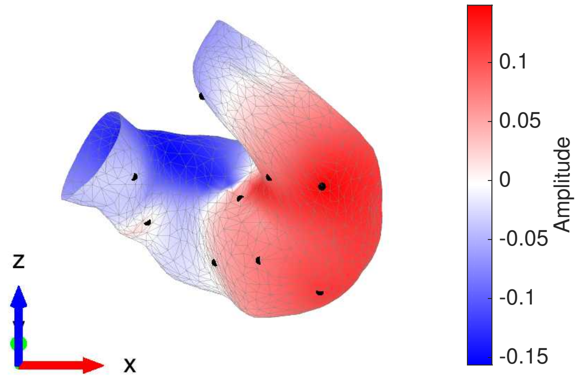

Subject A - IC 22

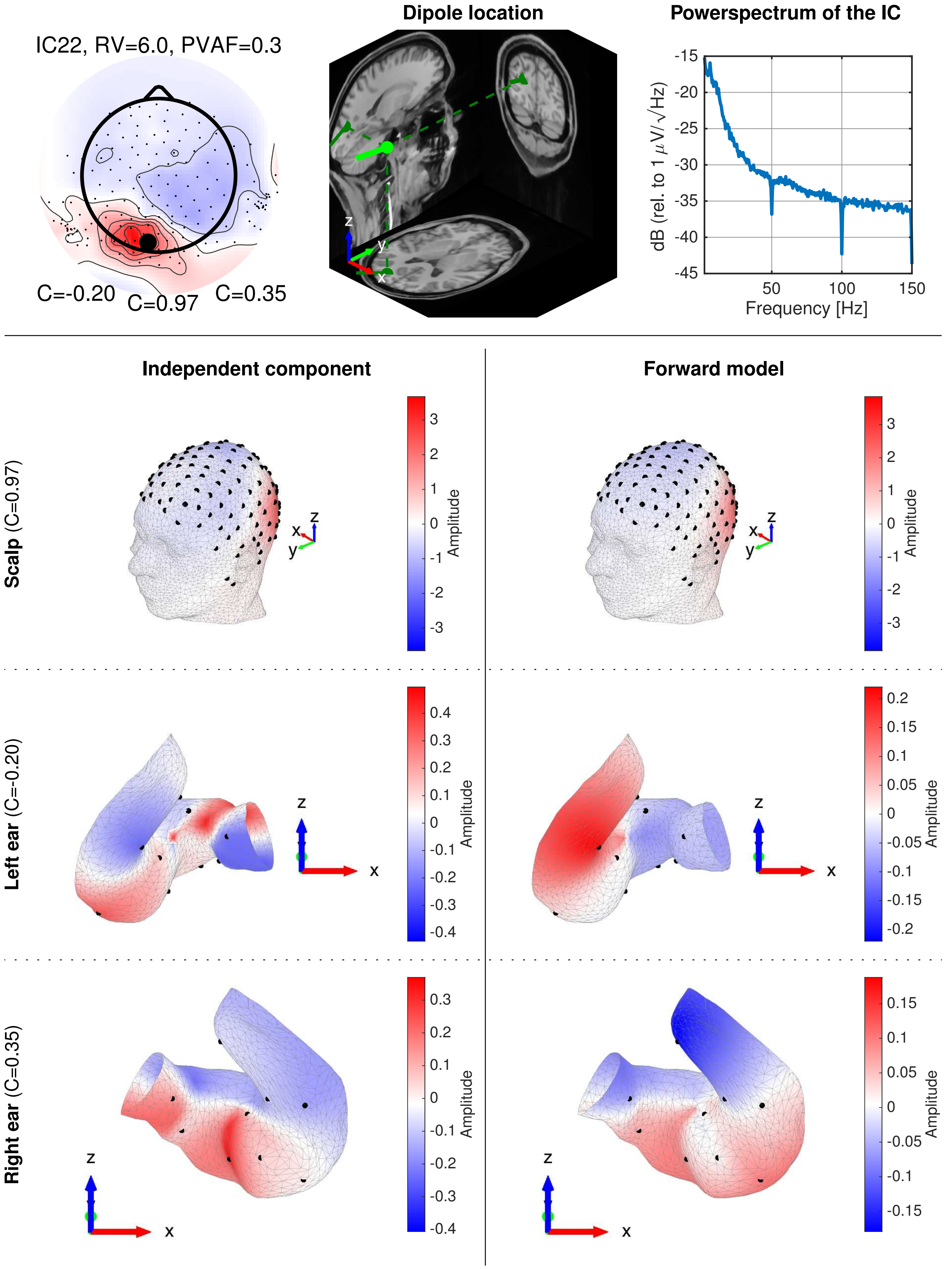

Subject A - IC 28

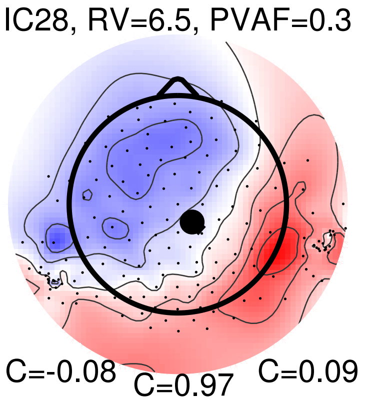

Dipole location

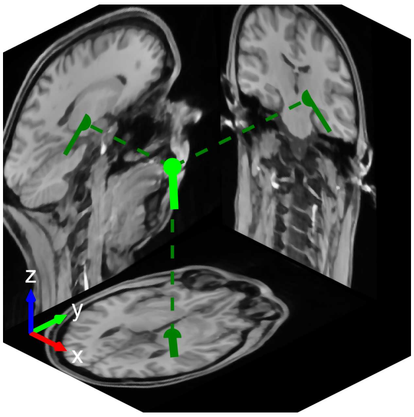

Powerspectrum of the IC

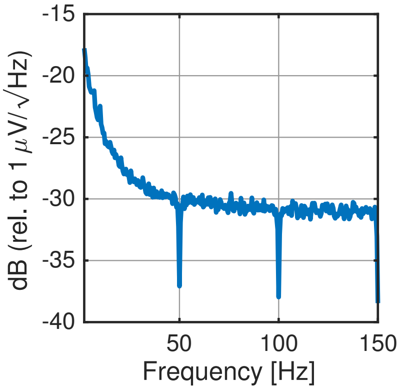

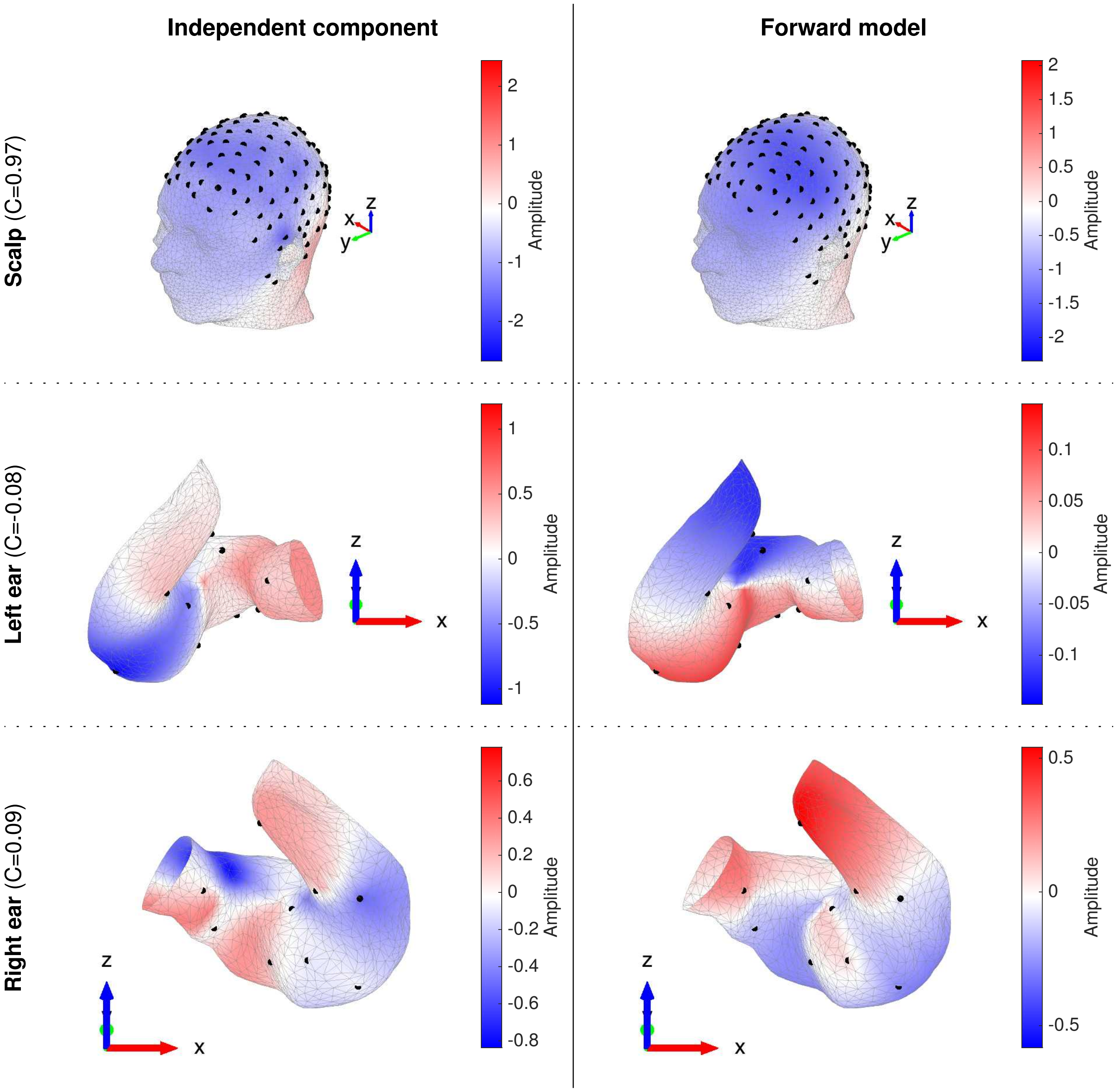

Subject A - IC 29

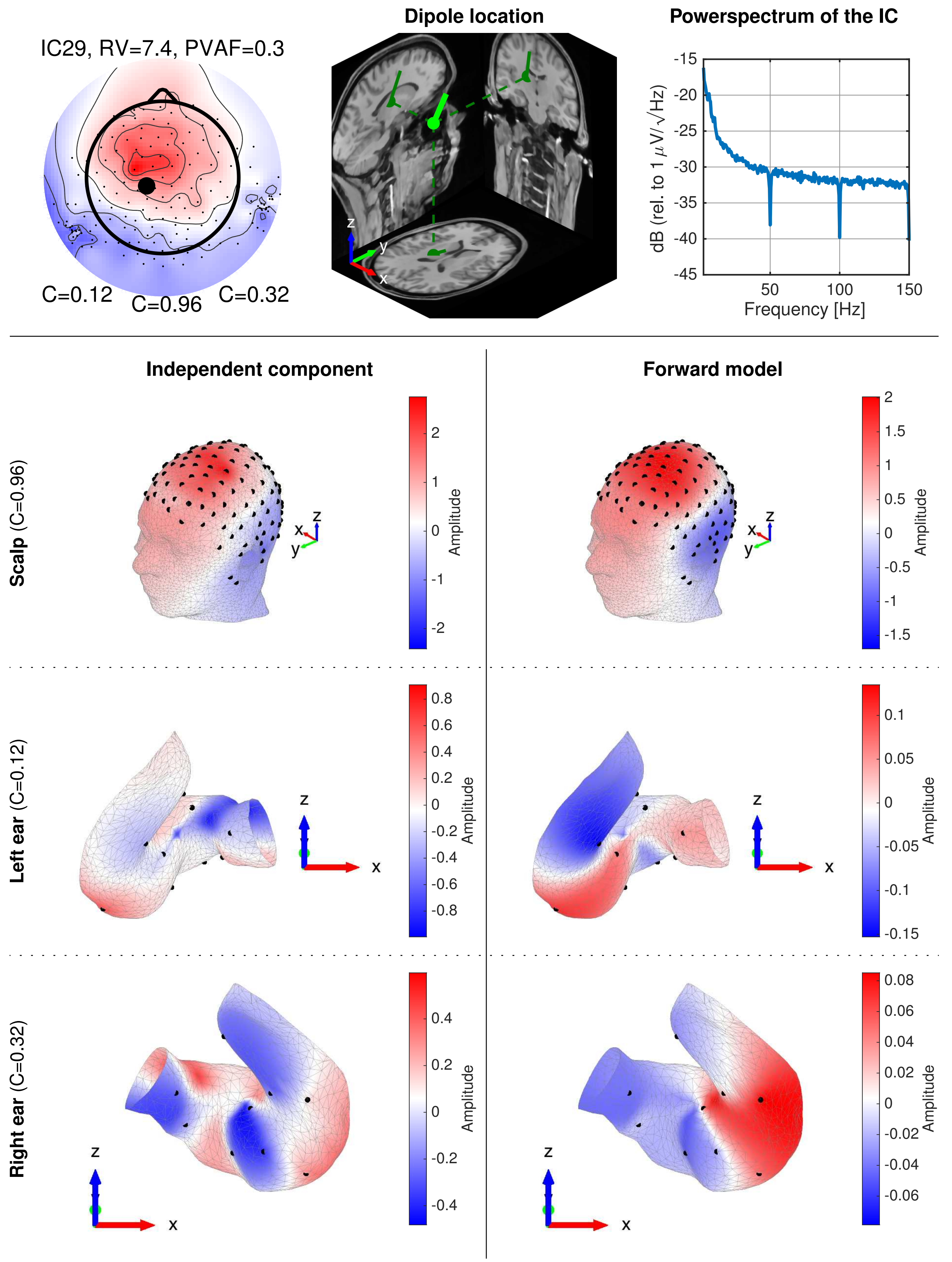

Subject A - IC 31

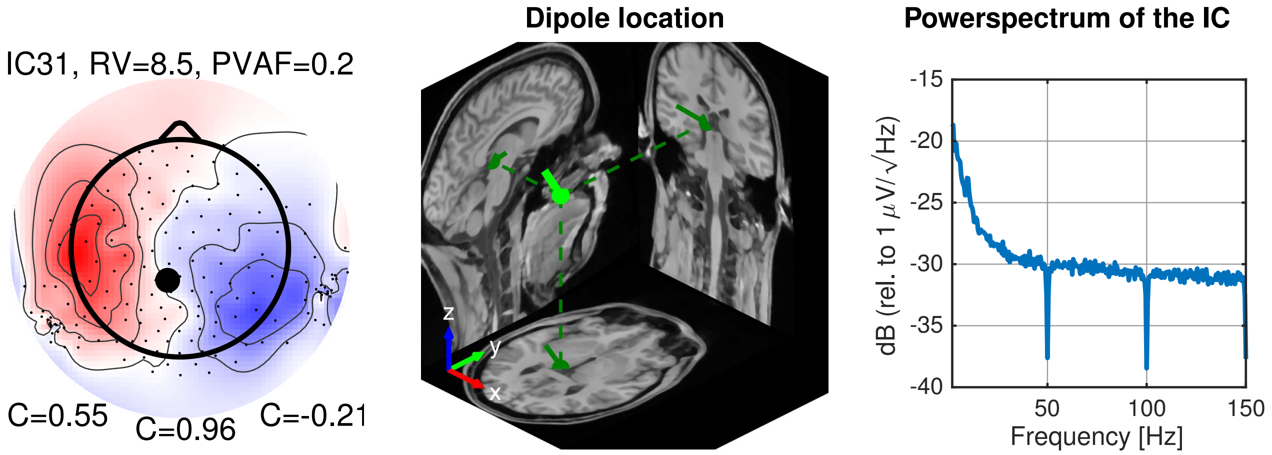

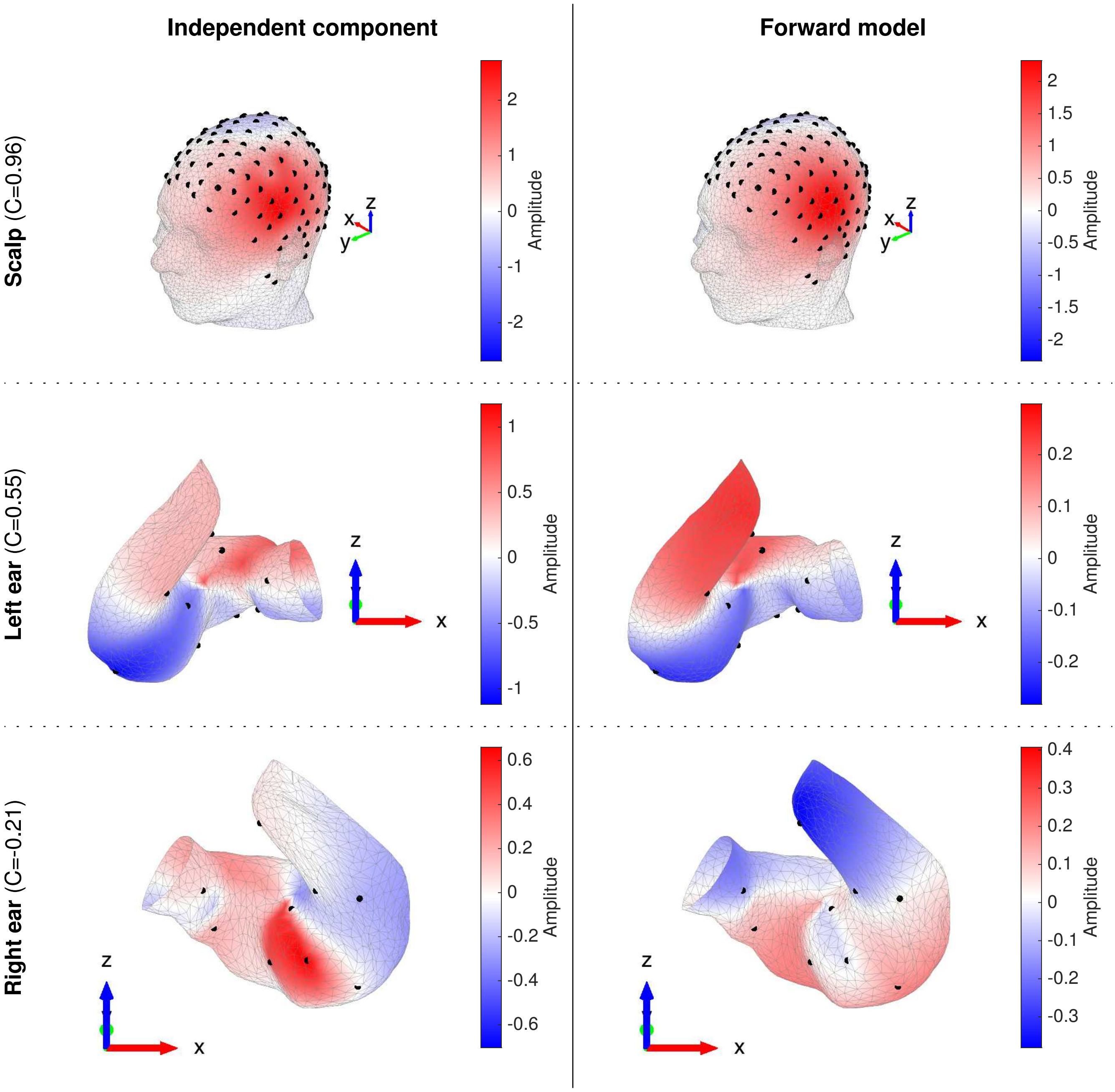

Subject A - IC 37

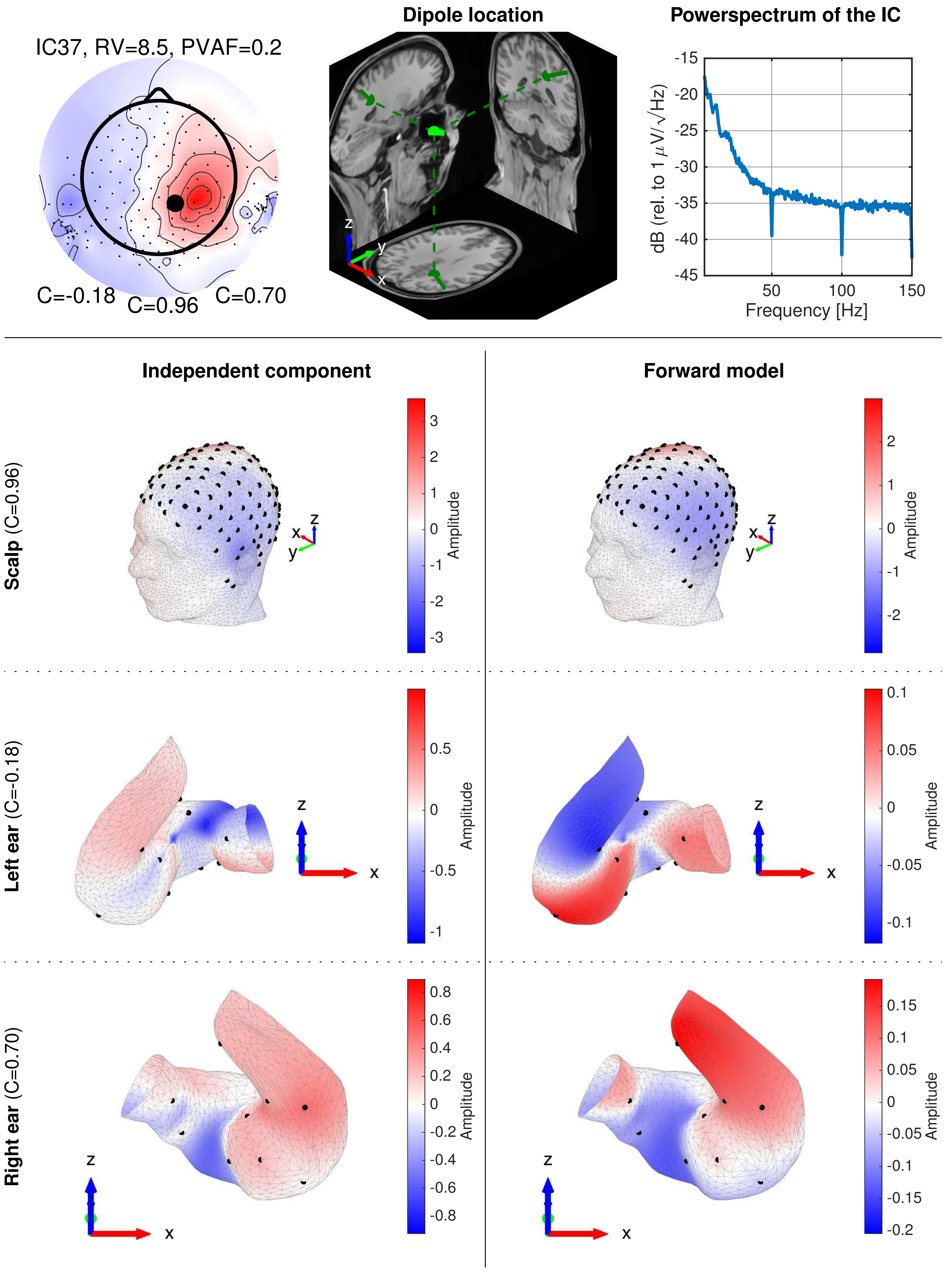

Subject A - IC 39

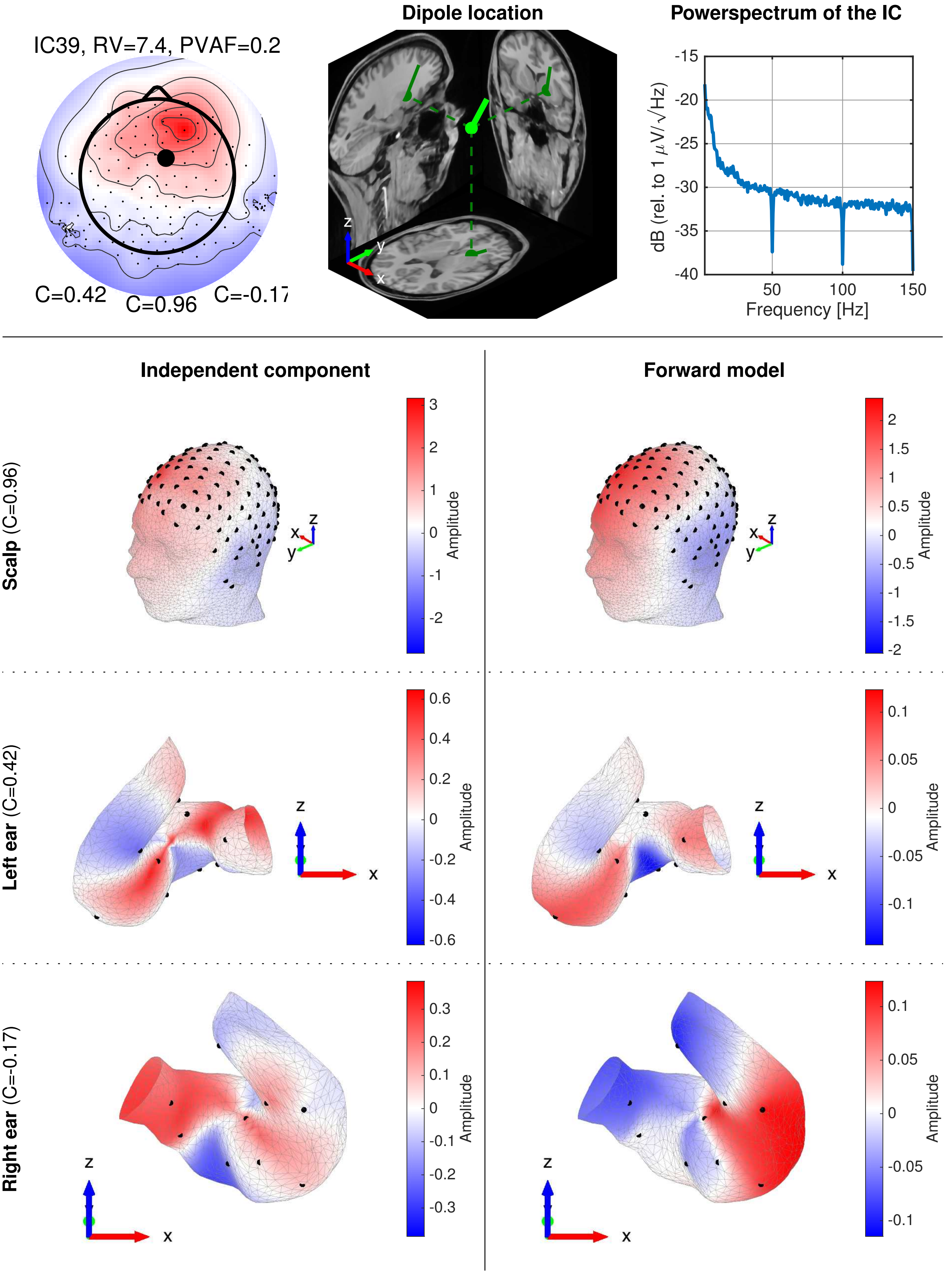

Subject A - IC 42

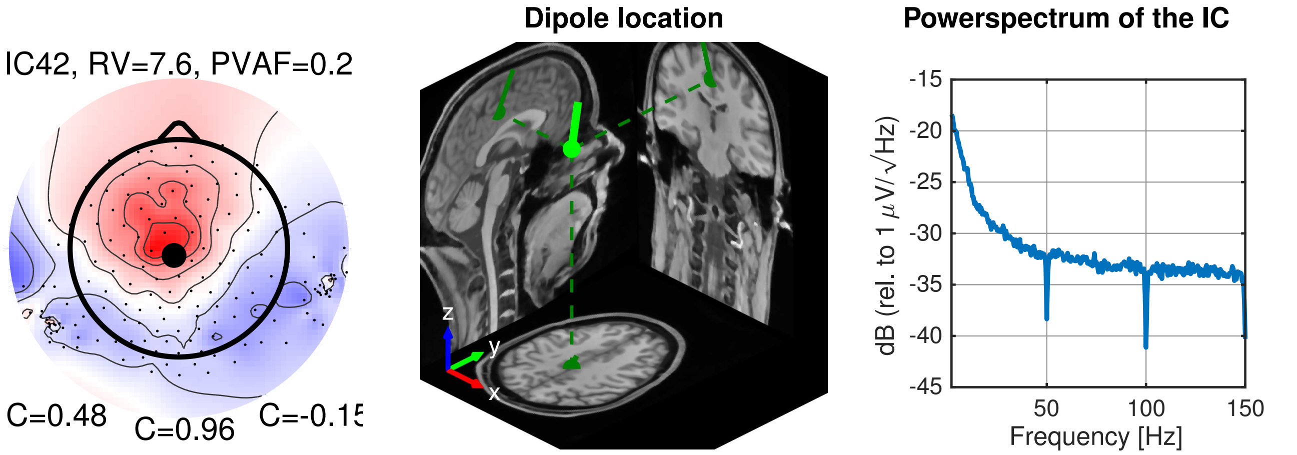

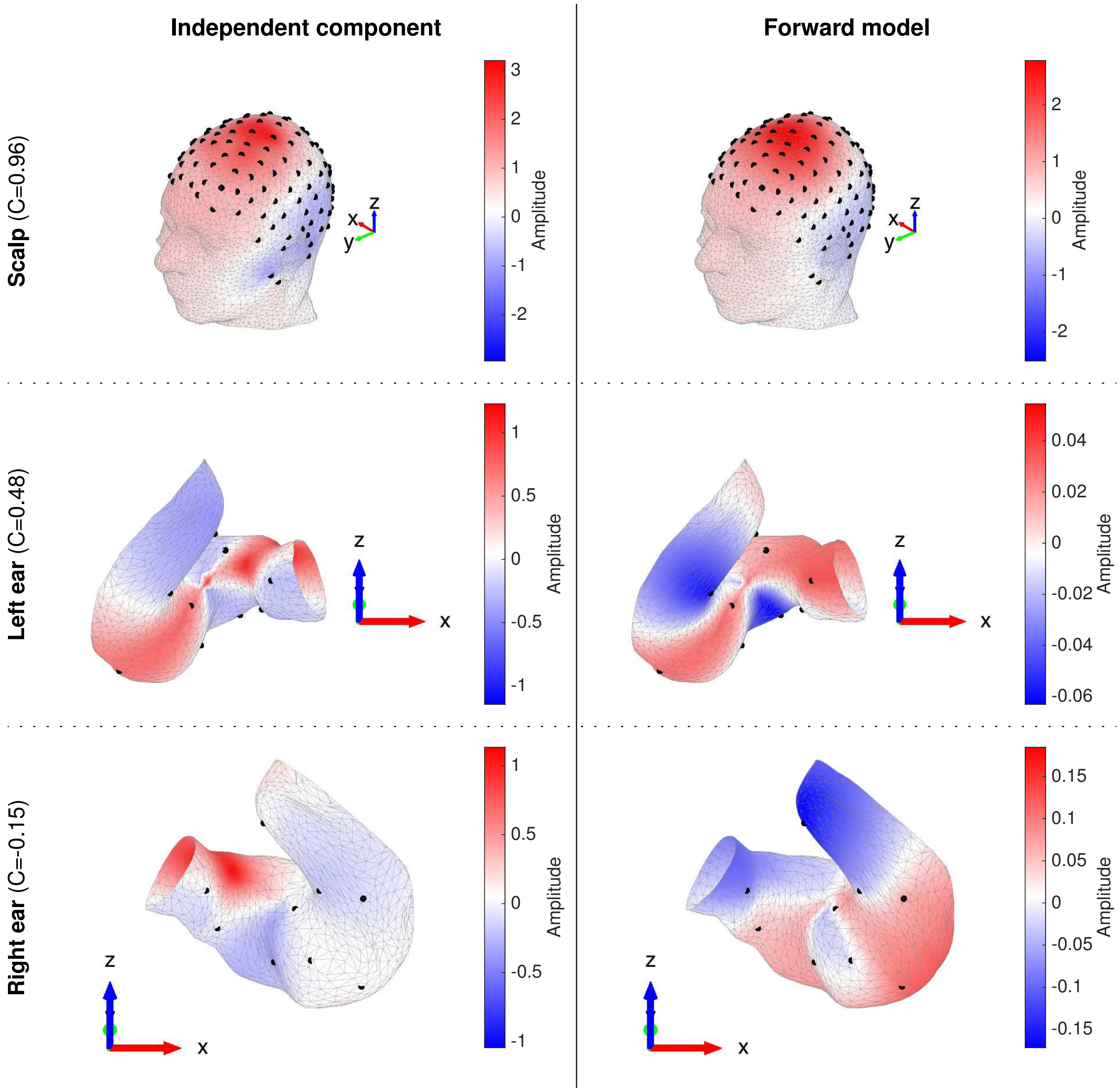

Subject A - IC 49

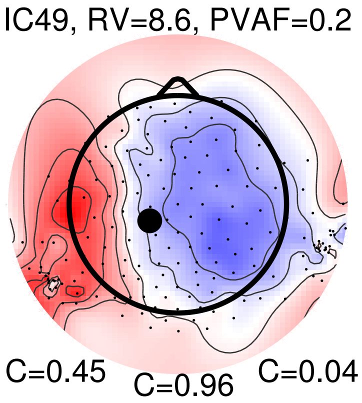

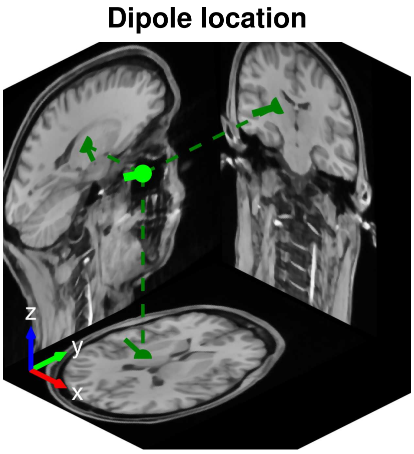

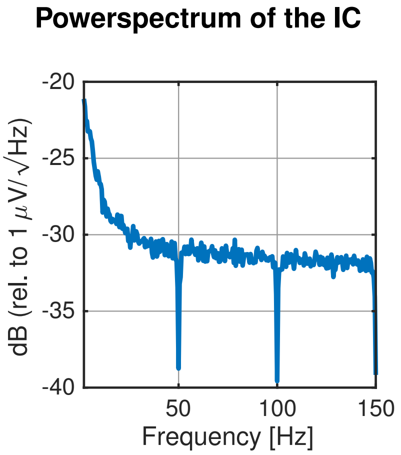

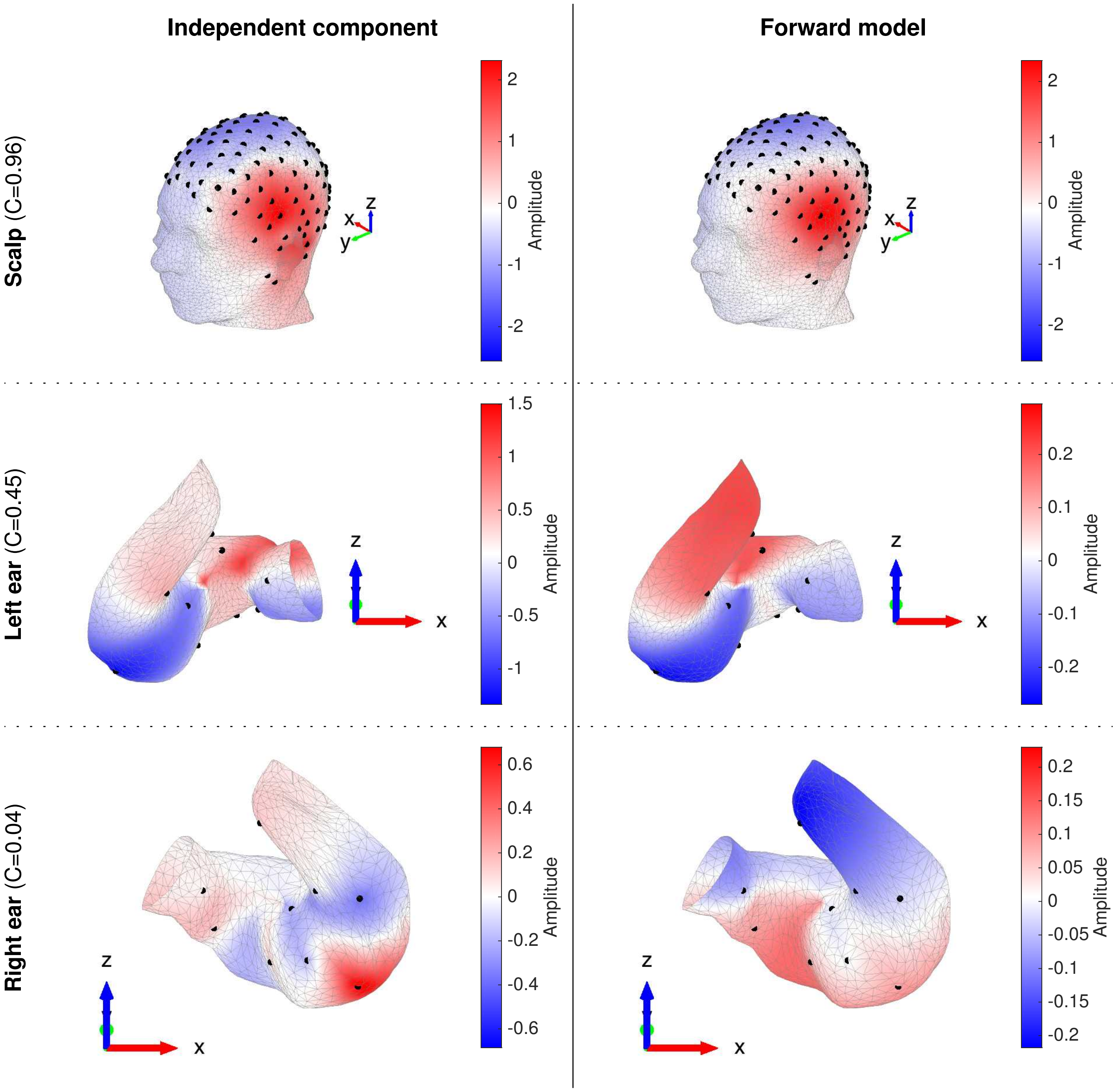

Subject A - IC 56

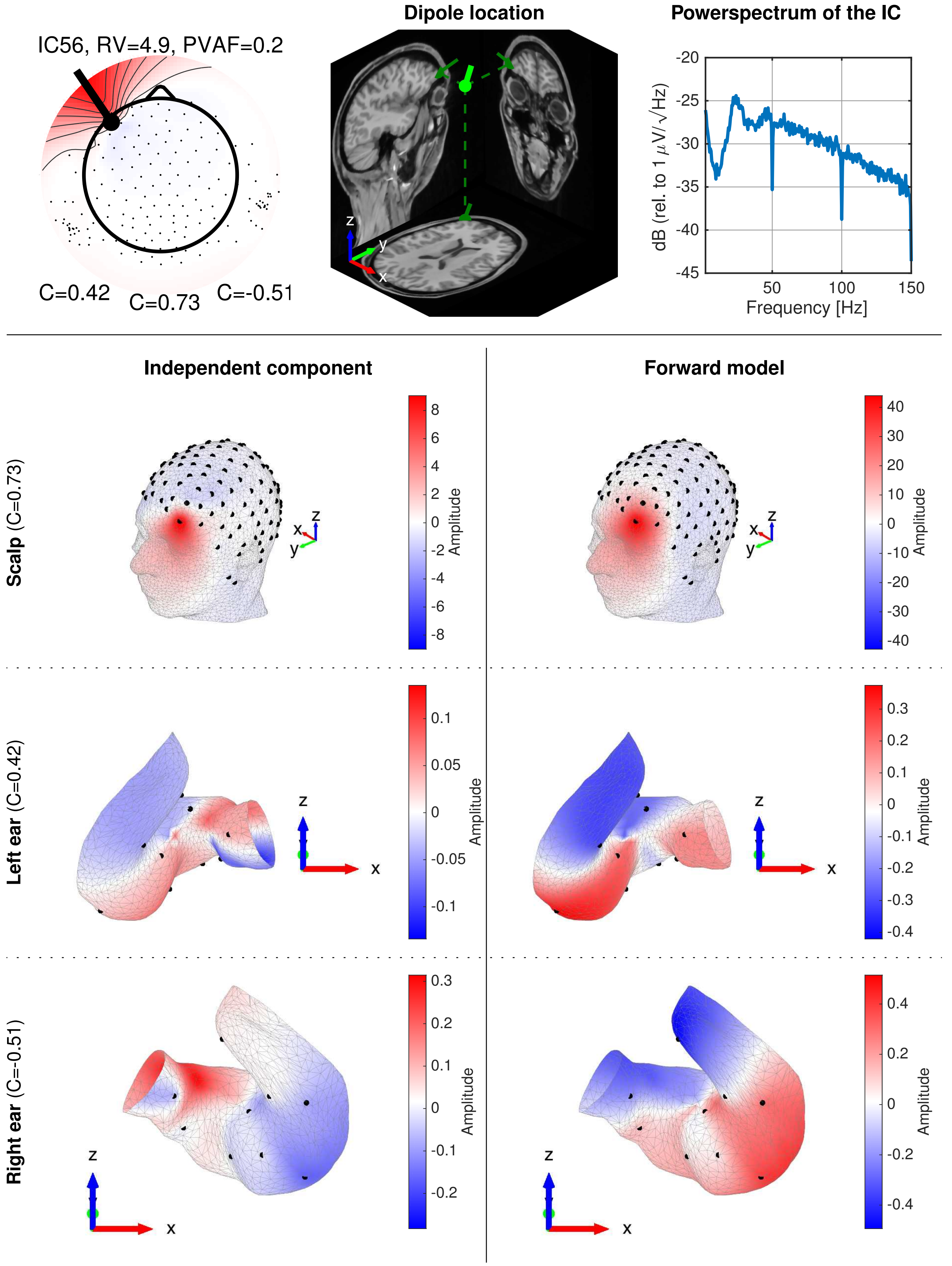

Subject A - IC 67

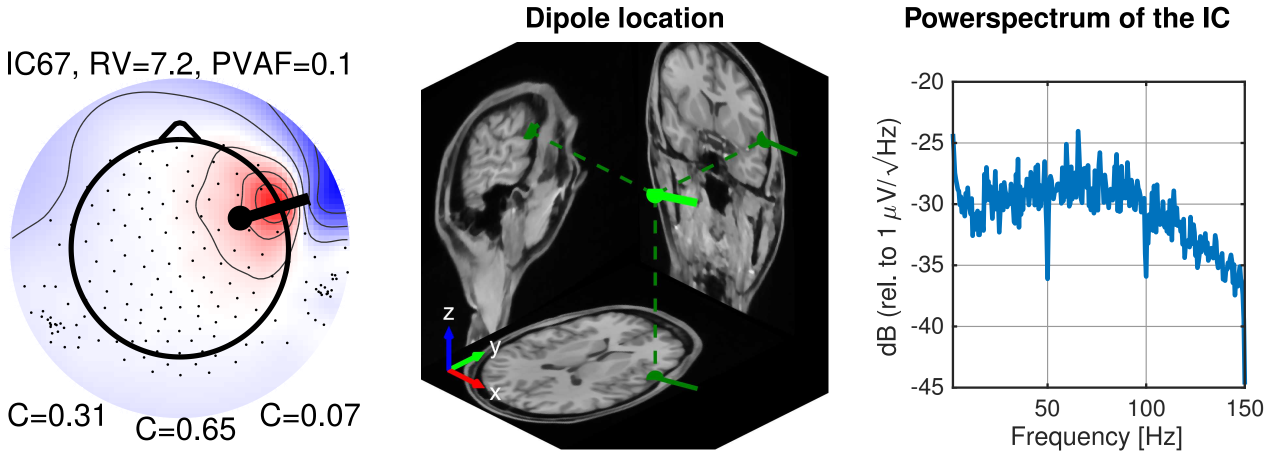

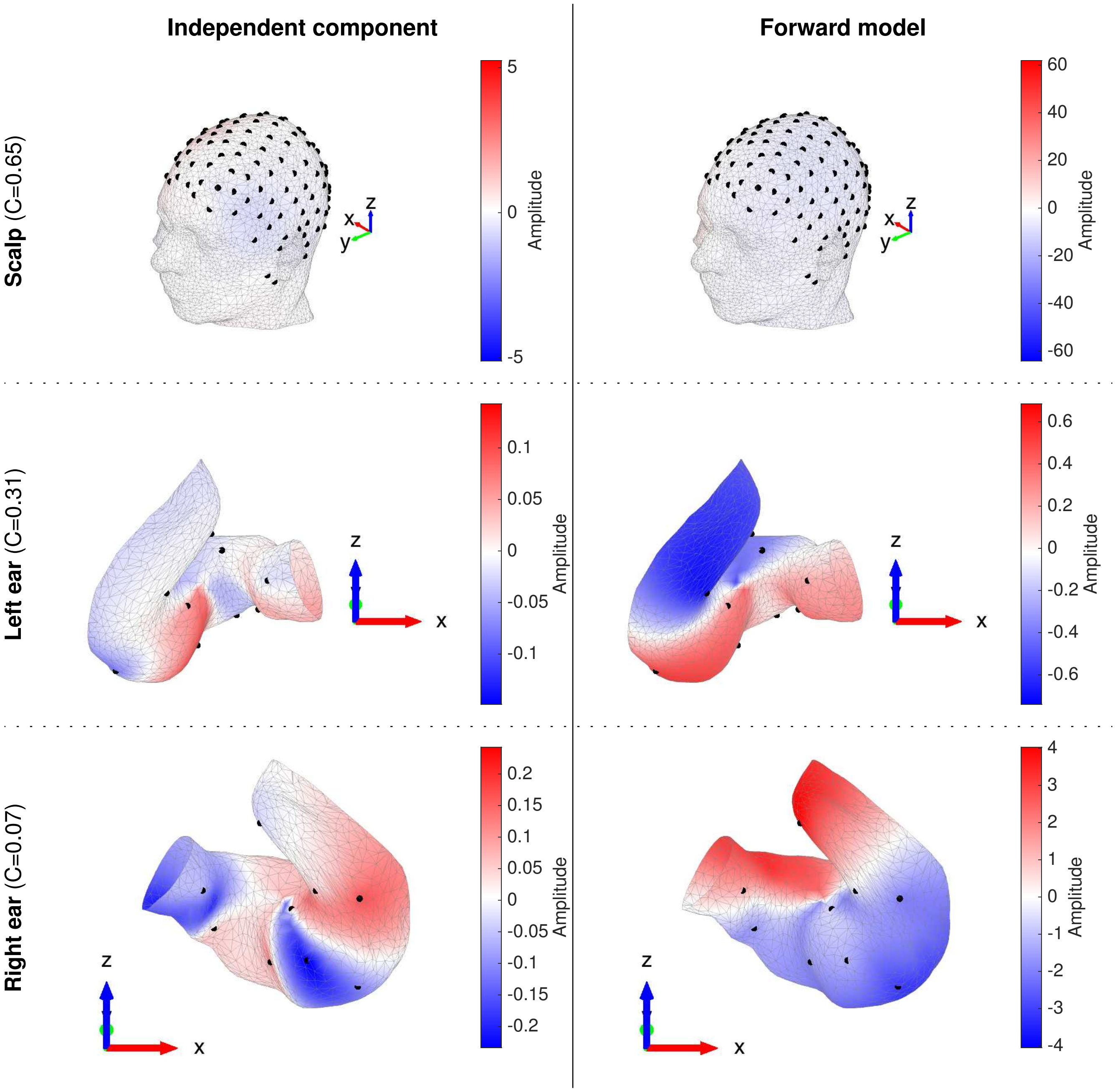

2 SUBJECT B

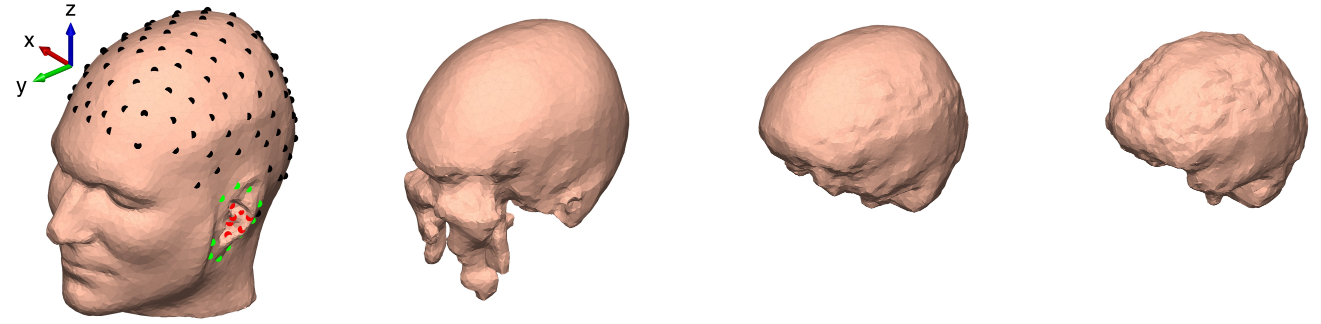

(a) Scalp (b) Outer skull (c) Inner skull (d) Brain

Figure S4. Headmodel mesh grids.

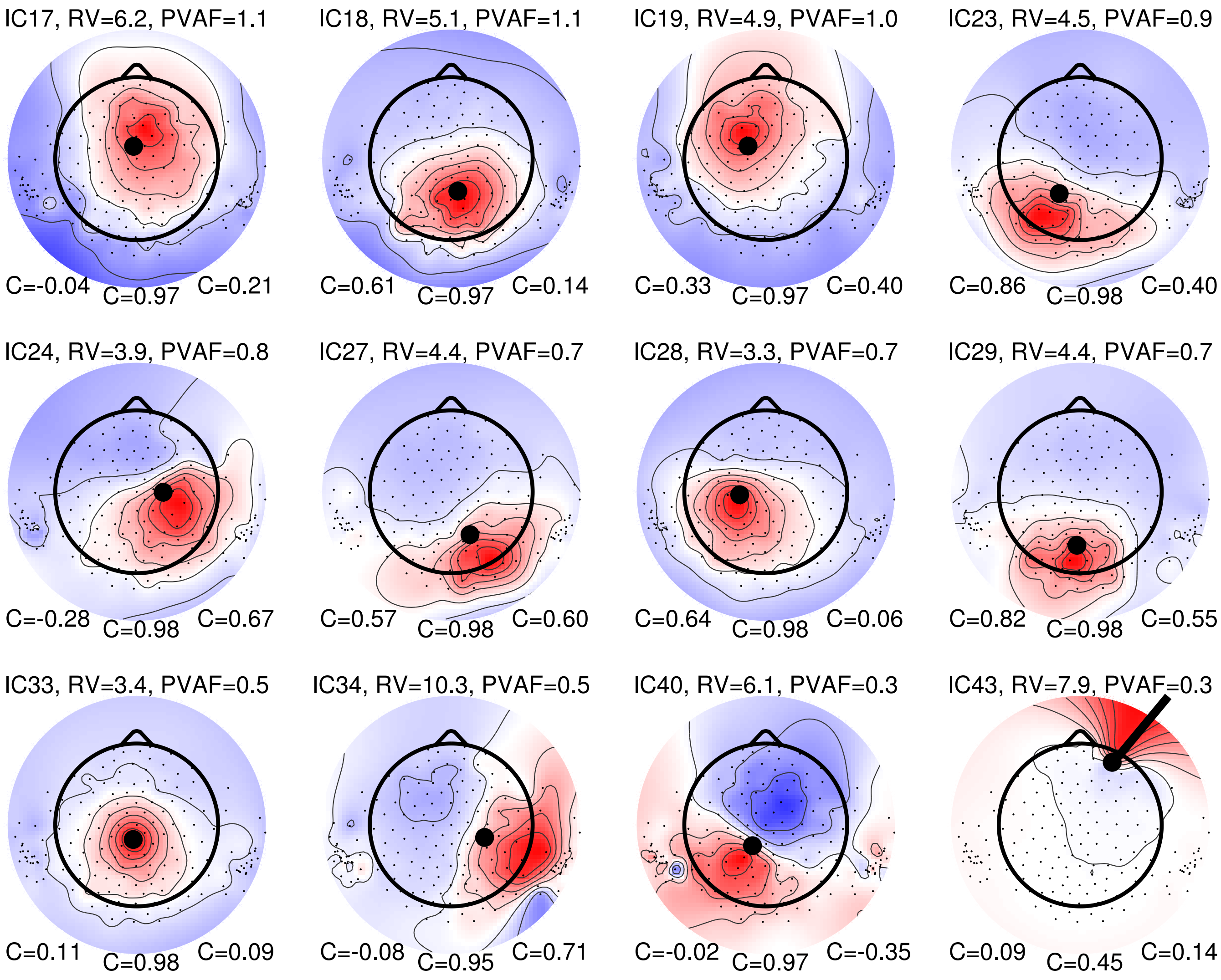

Figure S5. Topographic plots of the 12 independent components with the lowest residual variance.

Subject B - IC 17

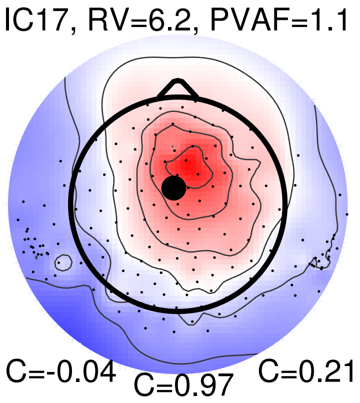

Dipole location

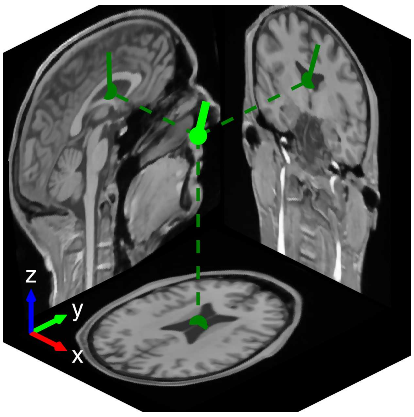

Powerspectrum of the IC

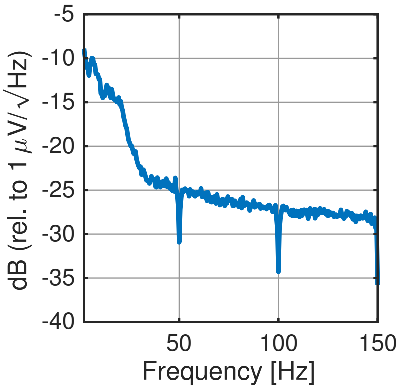

Independent component | **Forward model**

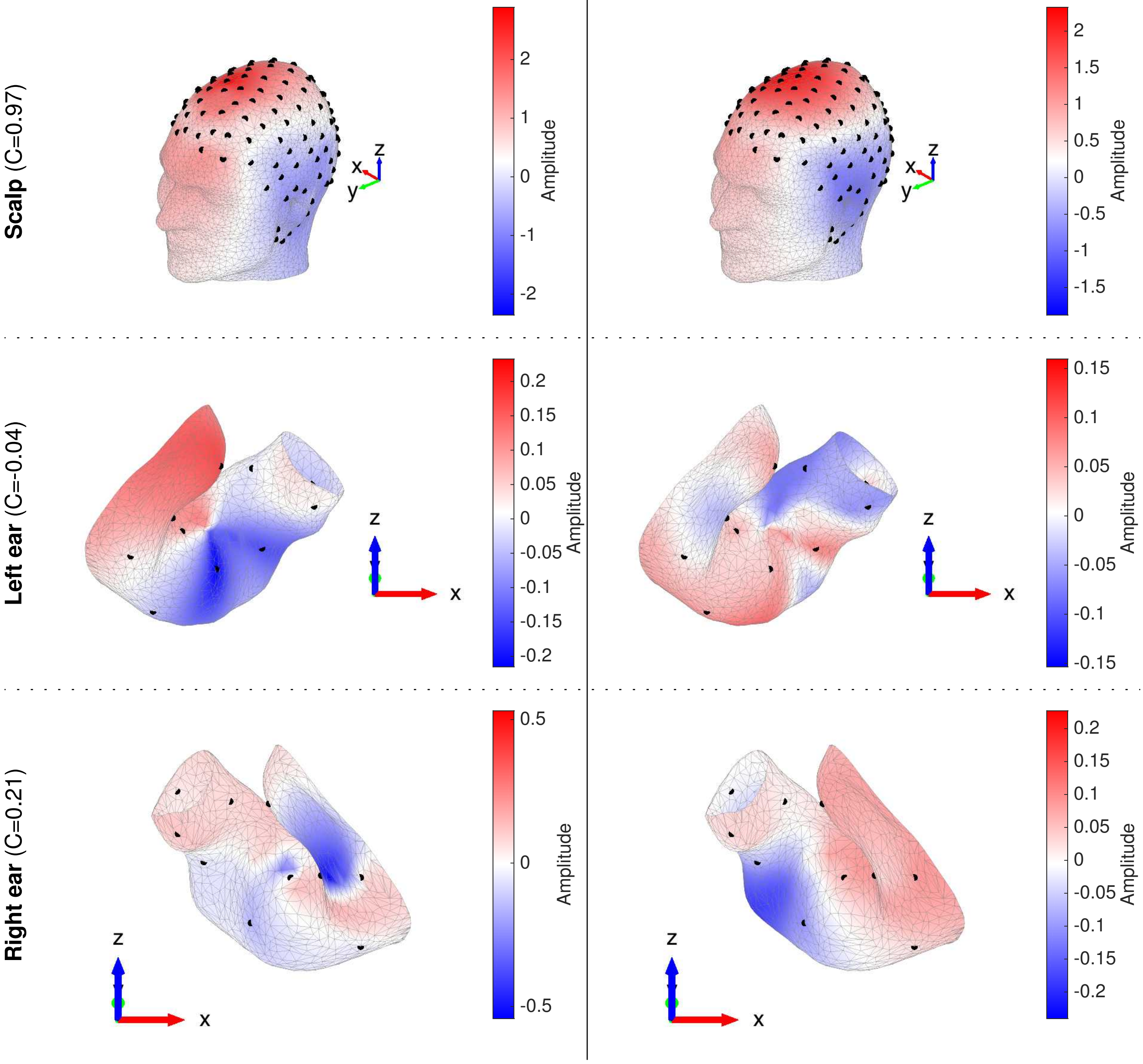

Subject B - IC 18

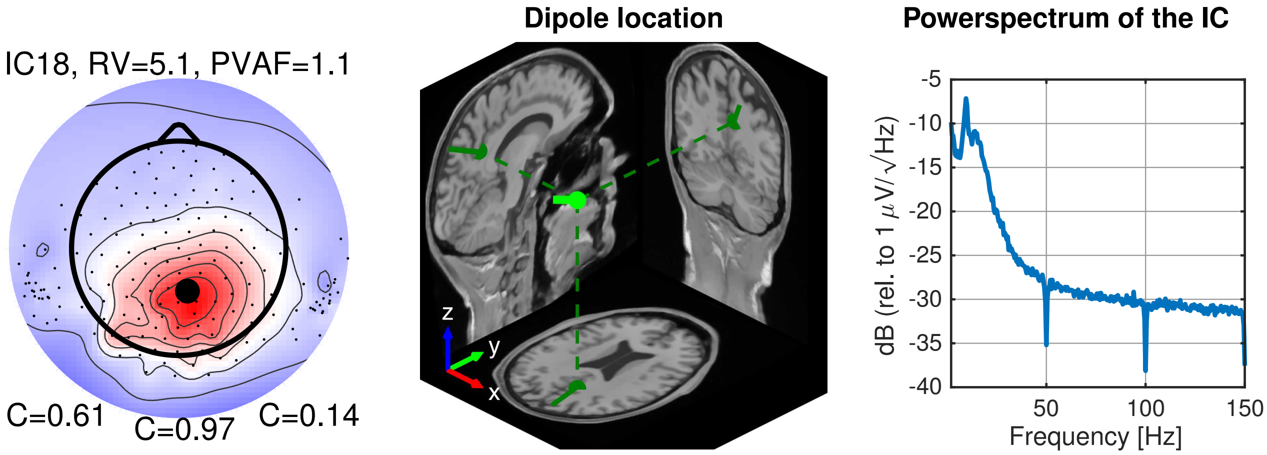

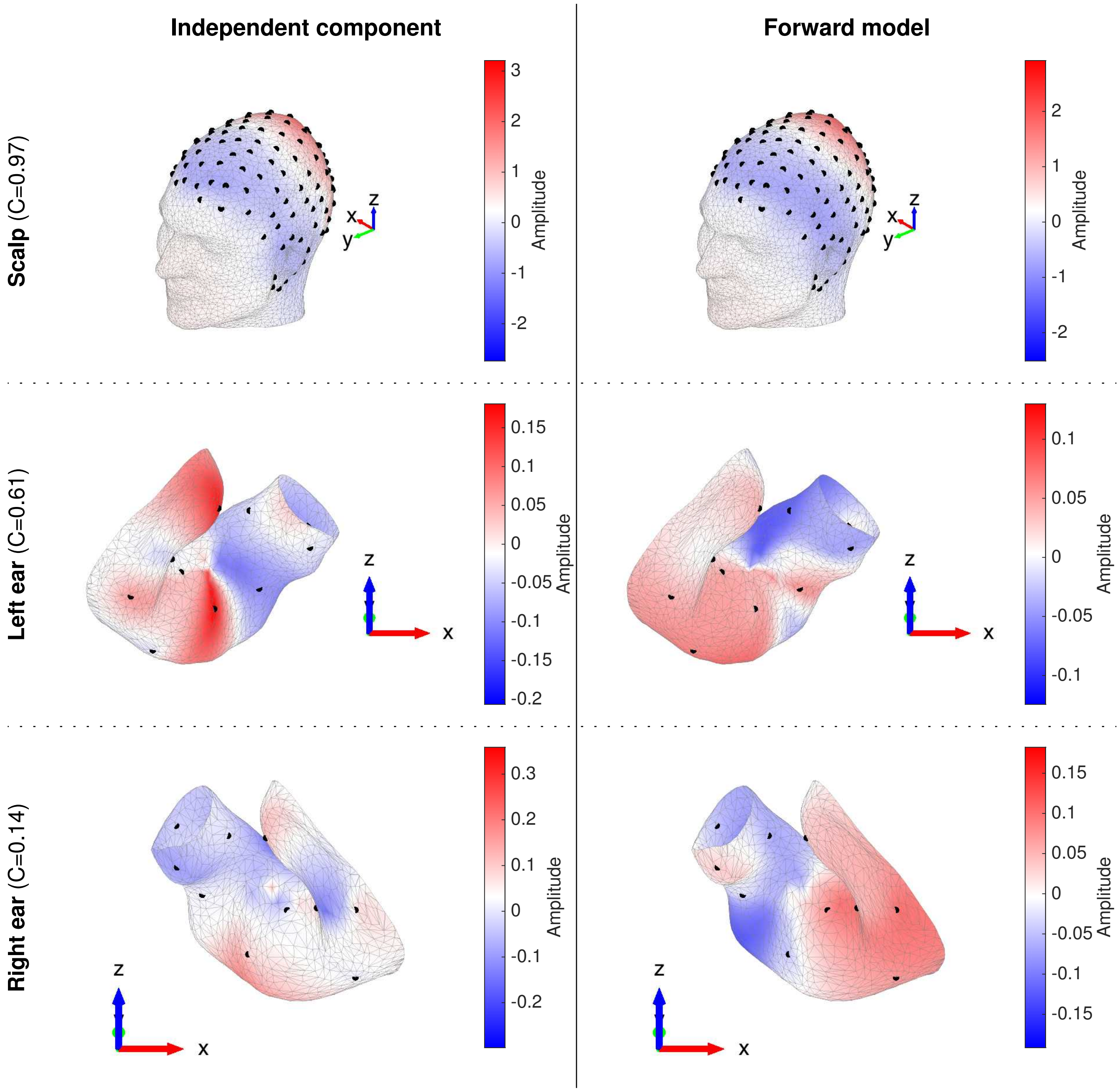

Subject B - IC 19

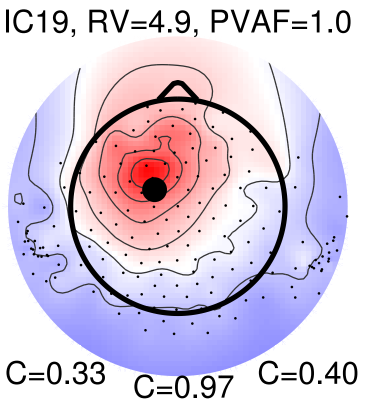

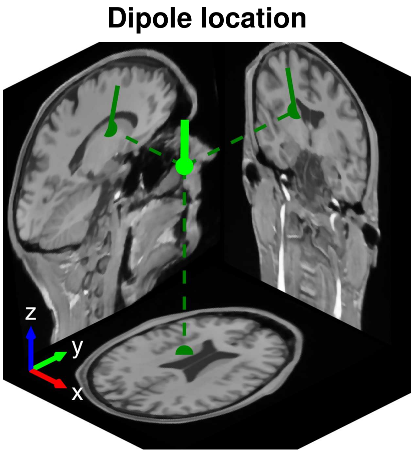

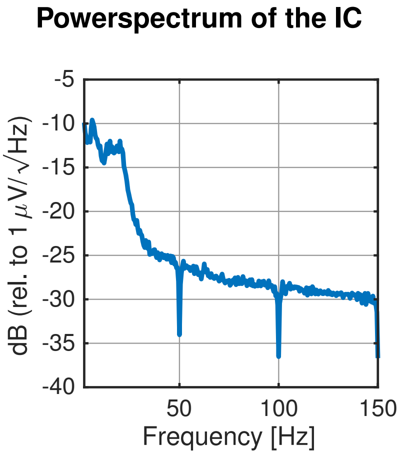

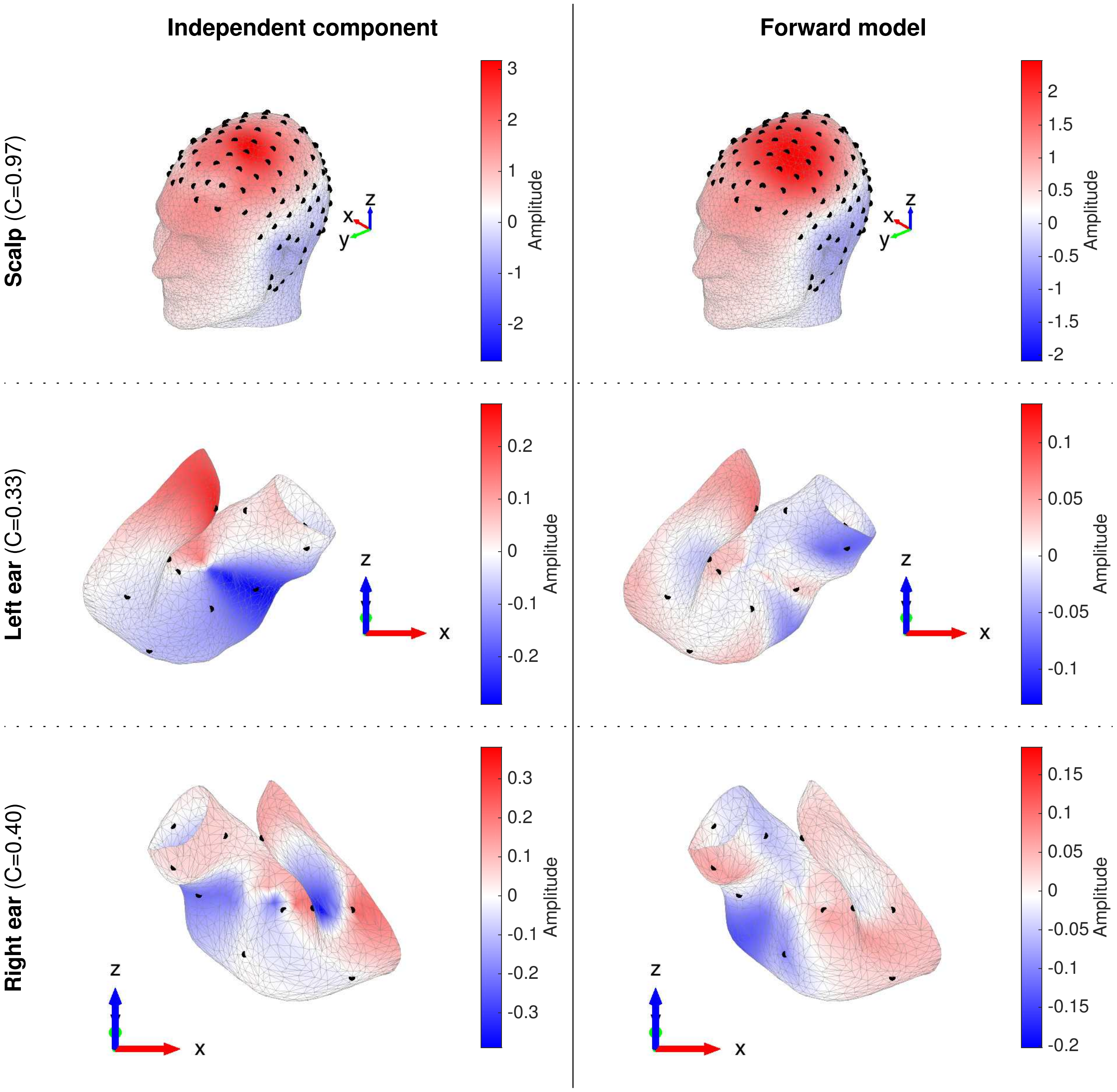

Subject B - IC 23

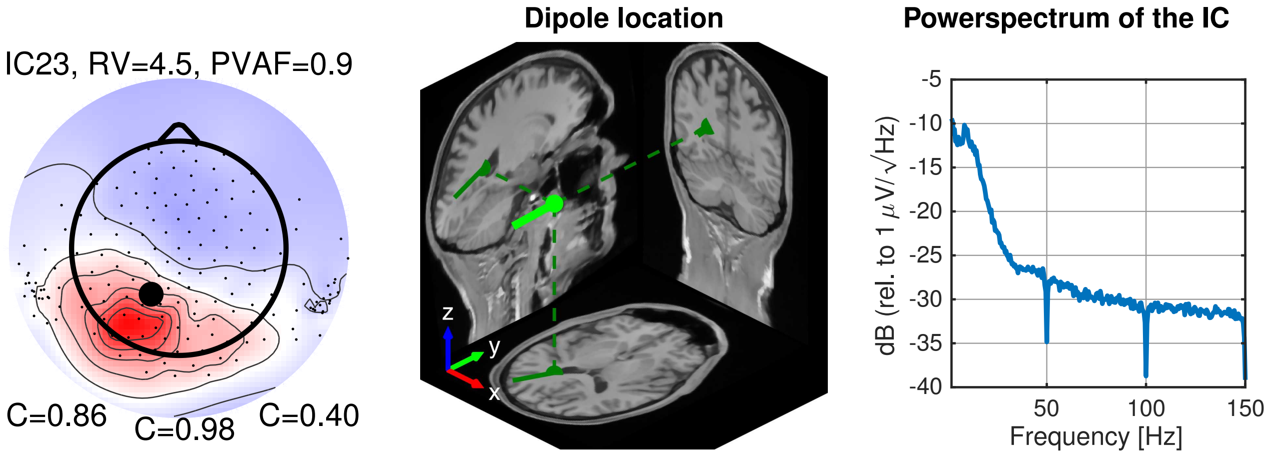

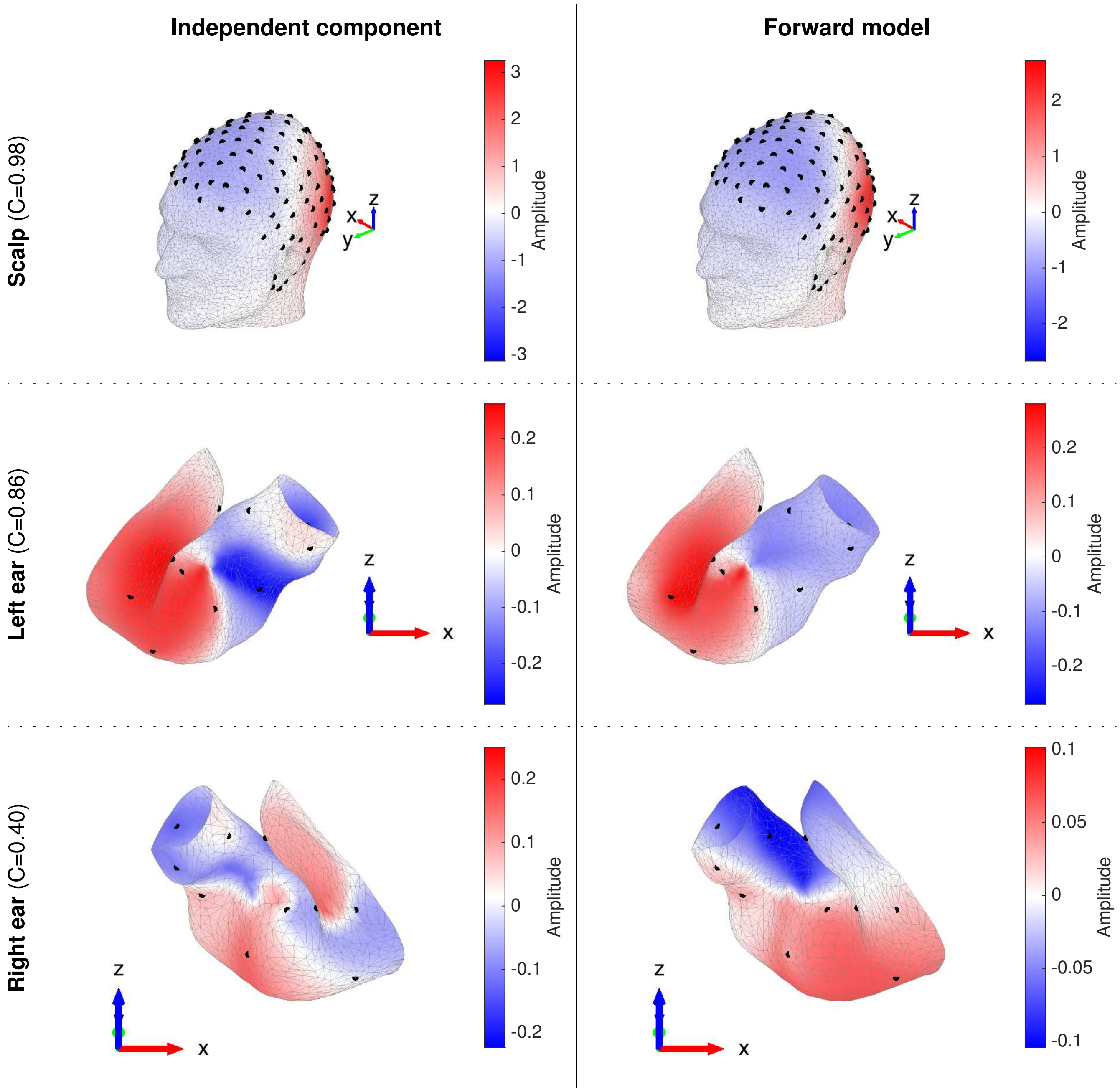

Subject B - IC 24

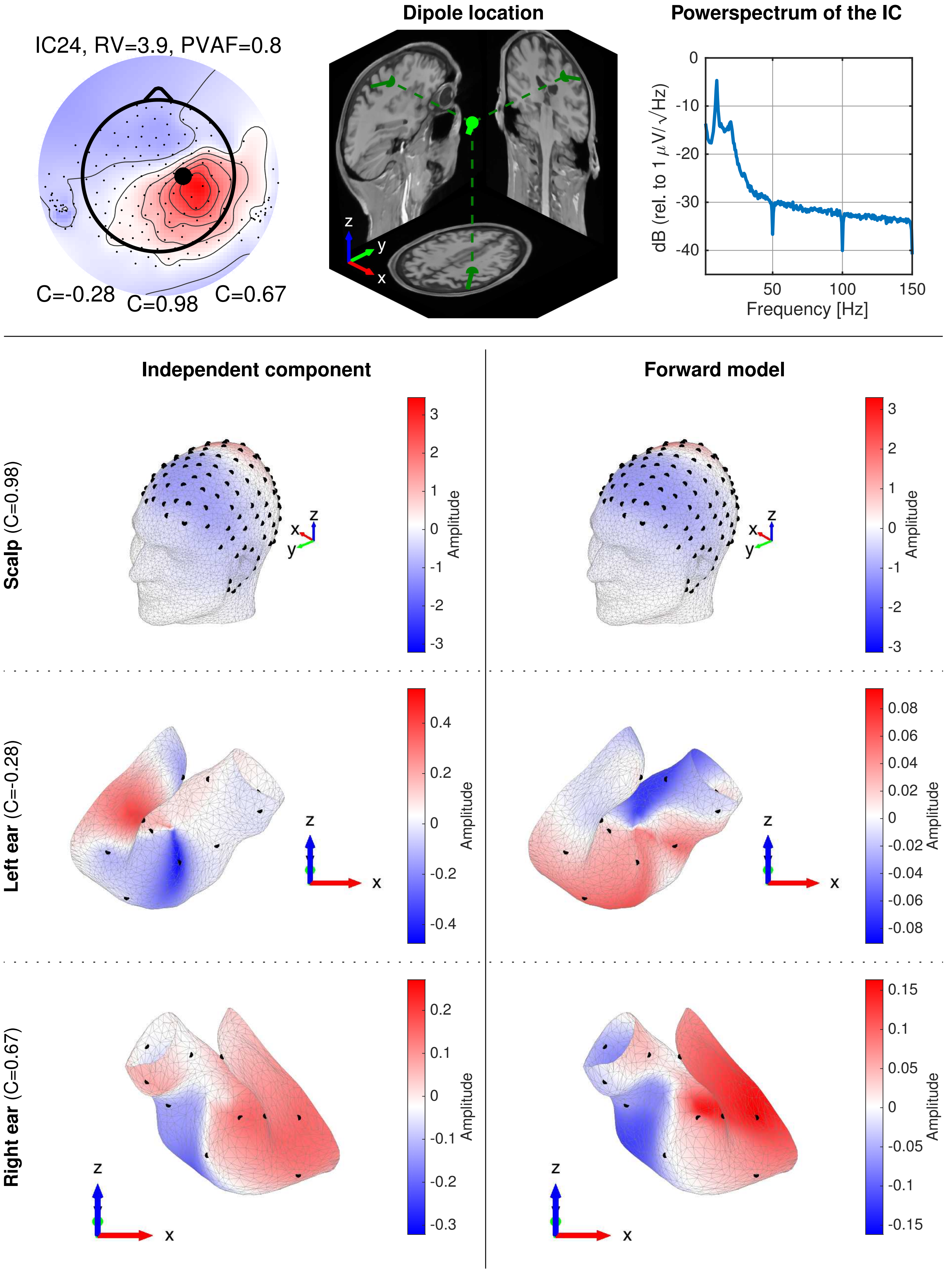

Subject B - IC 27

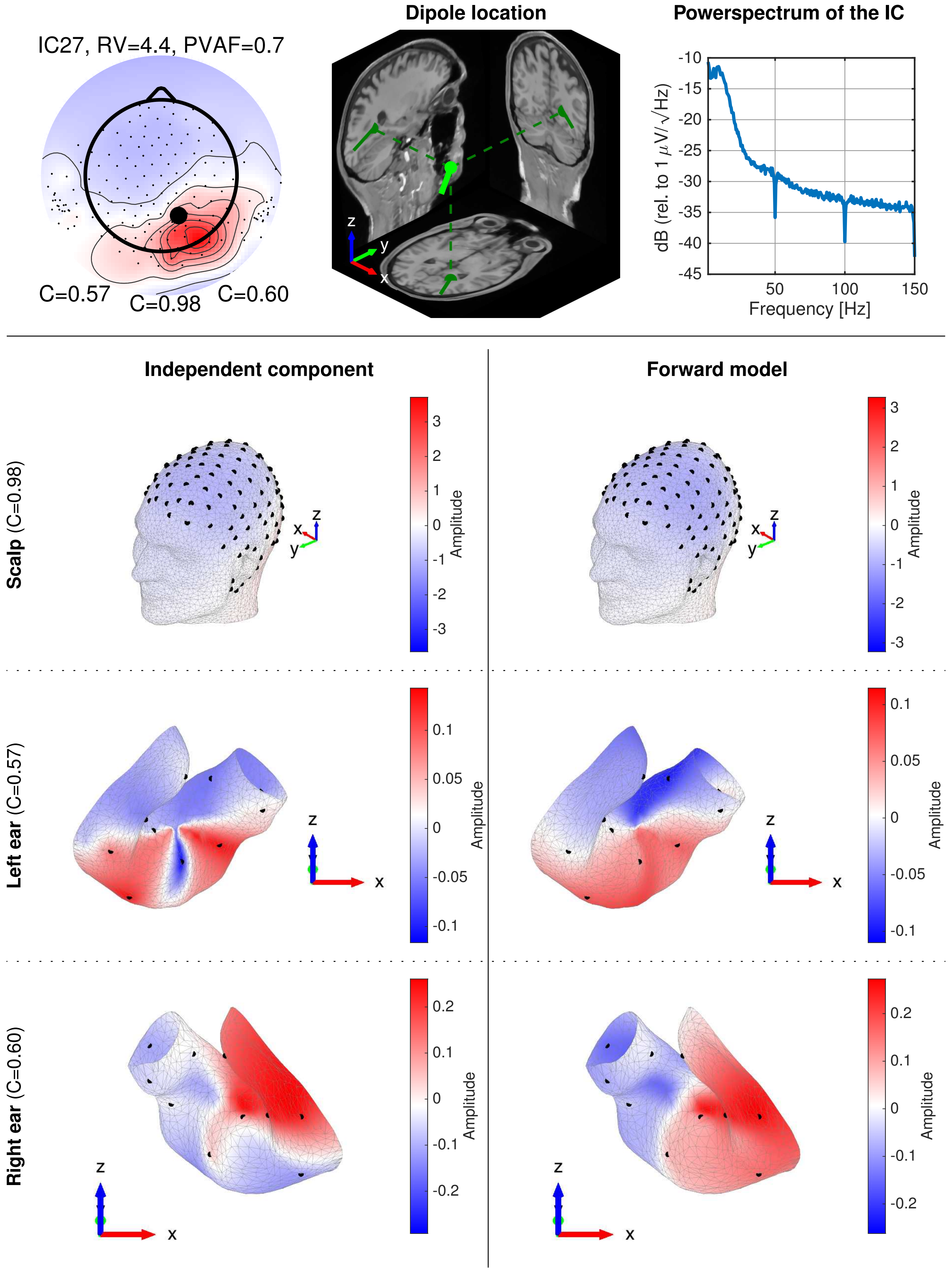

Subject B - IC 28

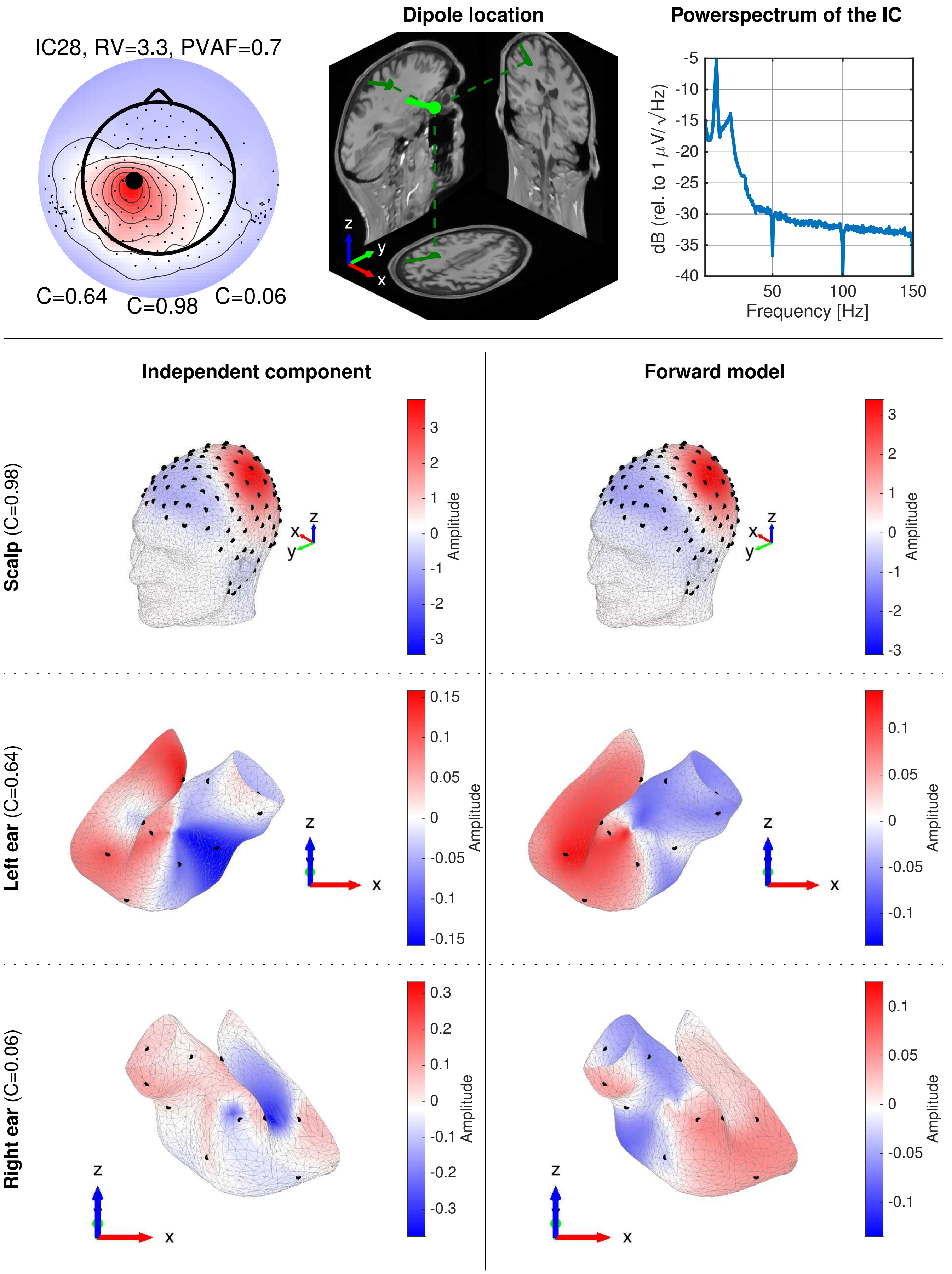

Subject B - IC 29

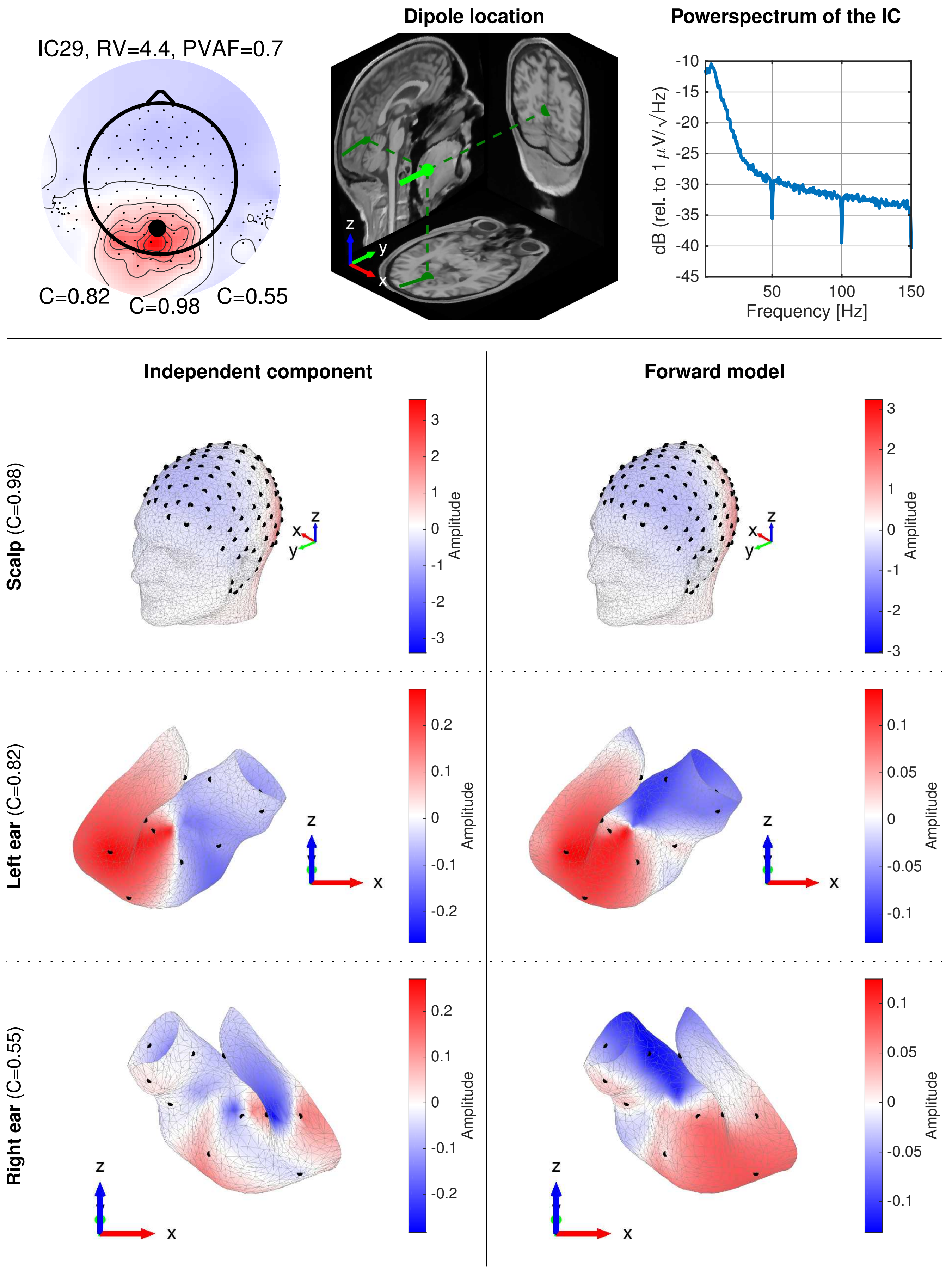

Subject B - IC 33

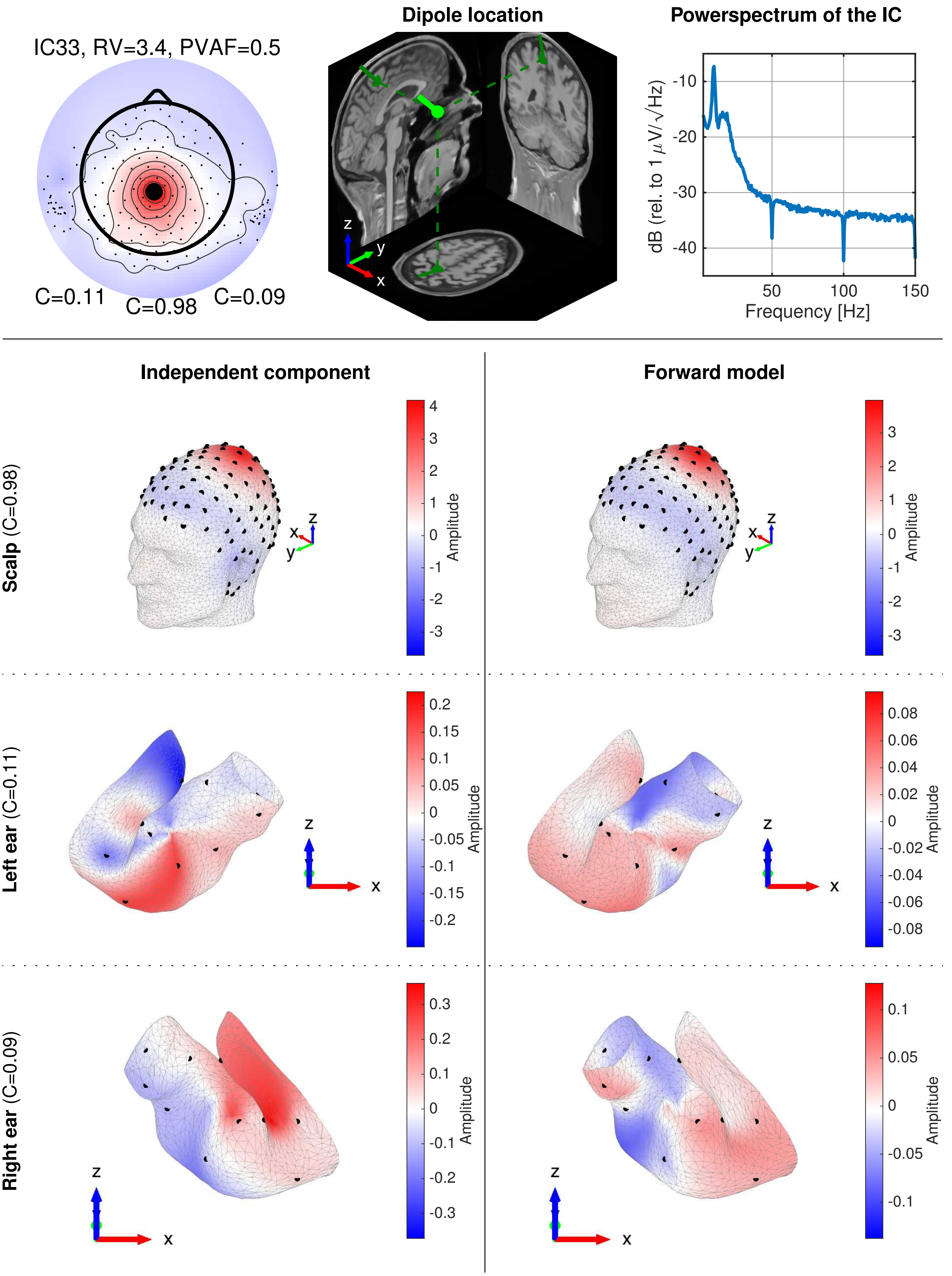

Subject B - IC 34

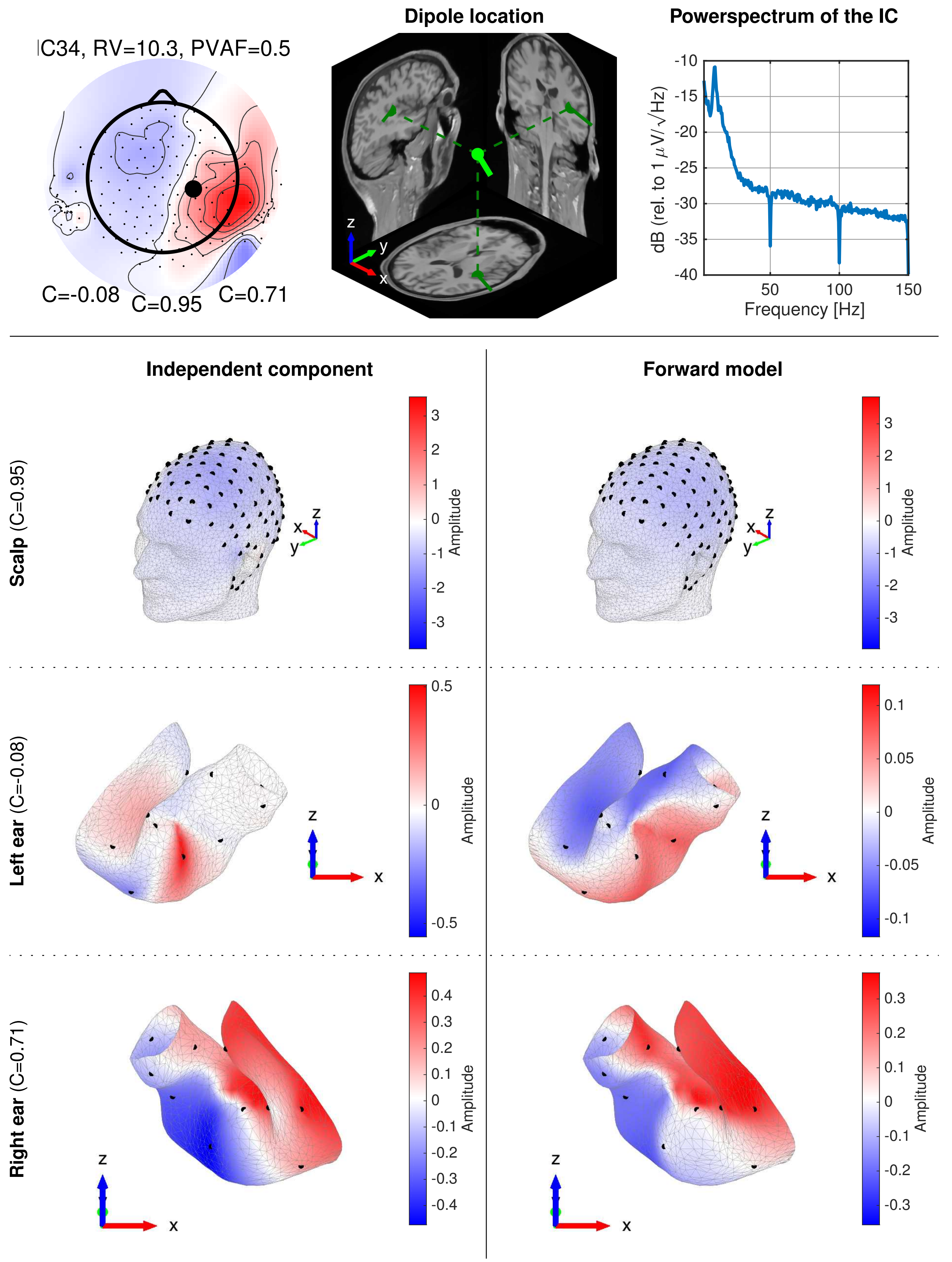

Subject B - IC 40

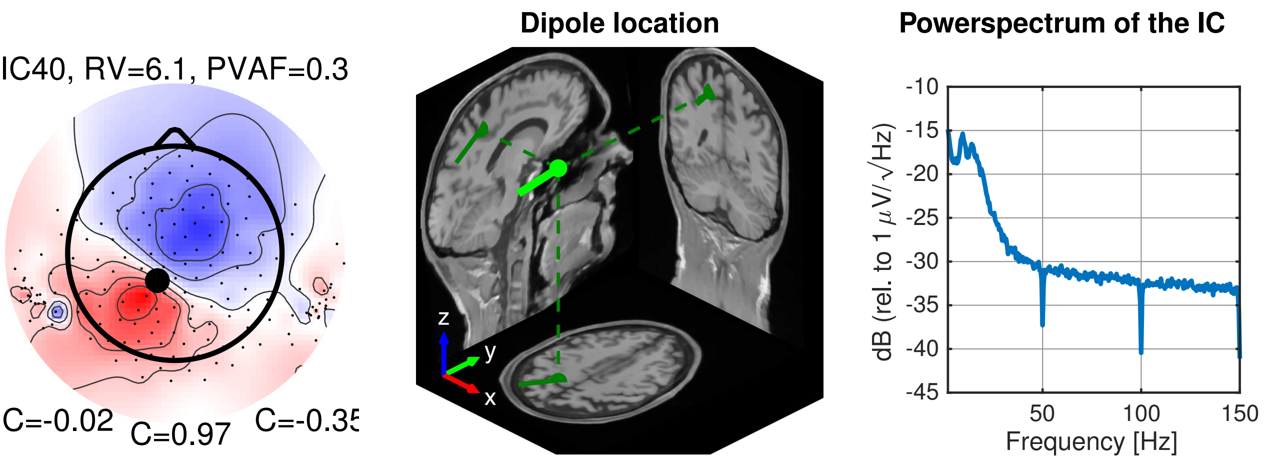

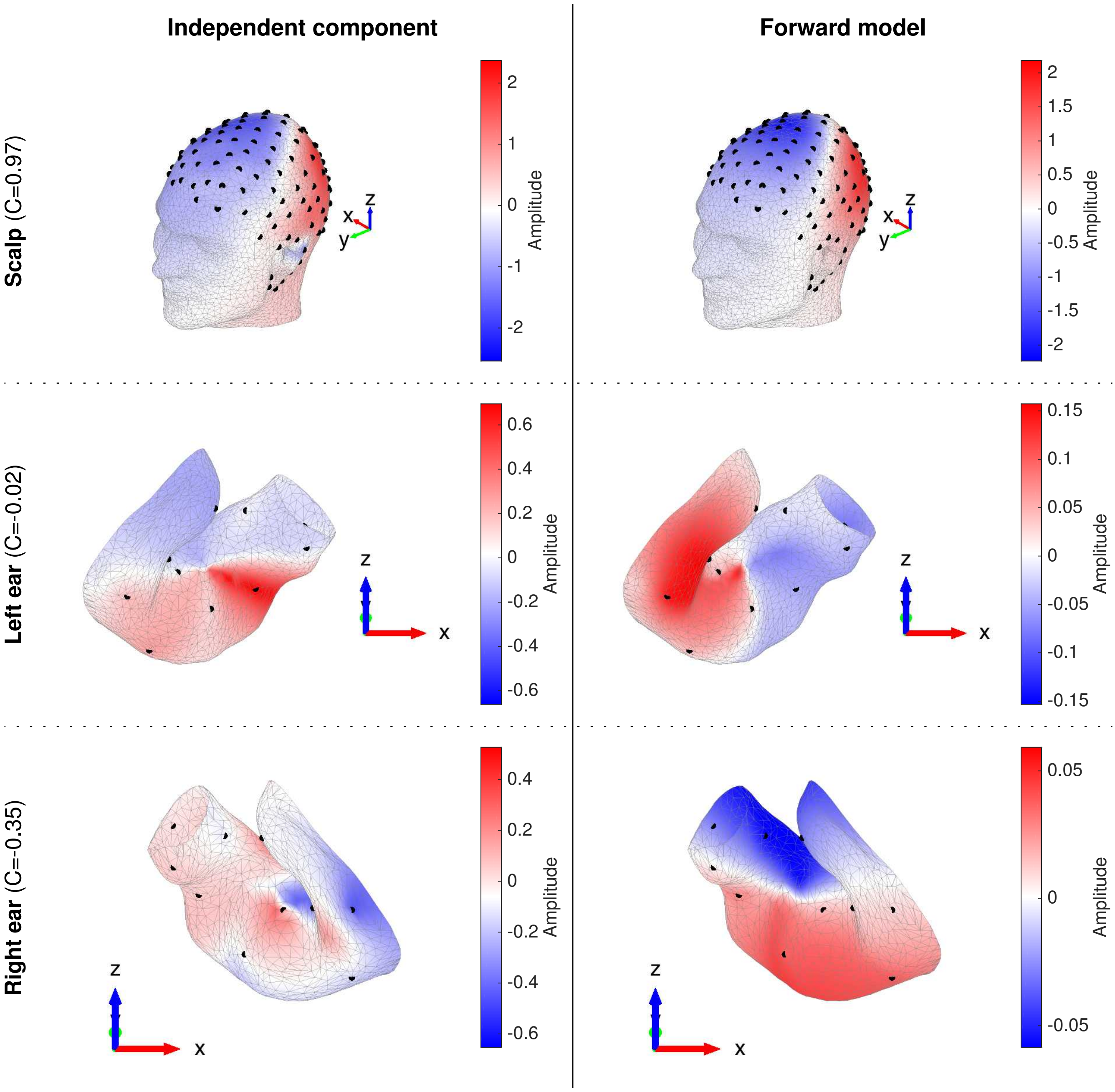

Subject B - IC 43

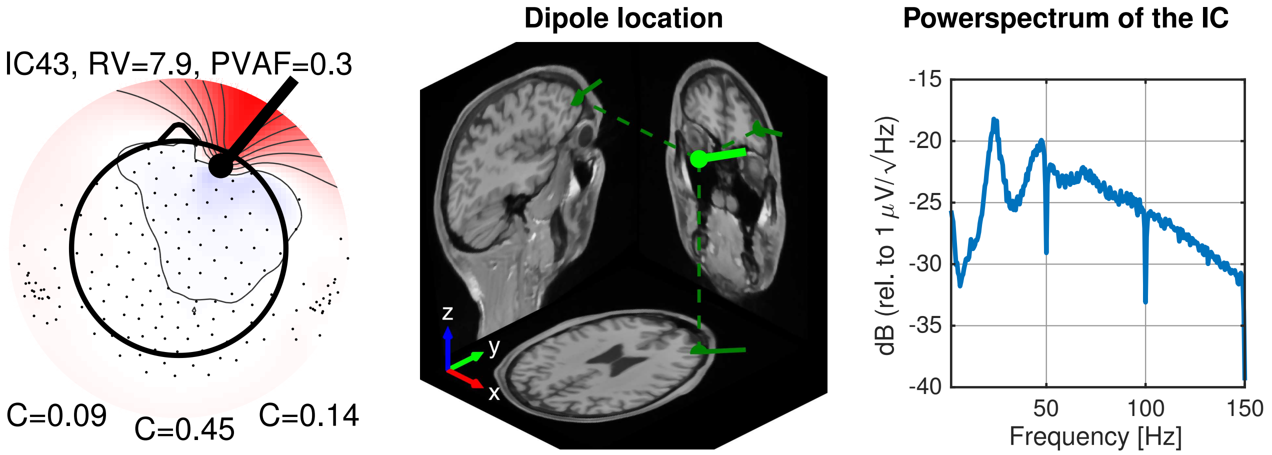

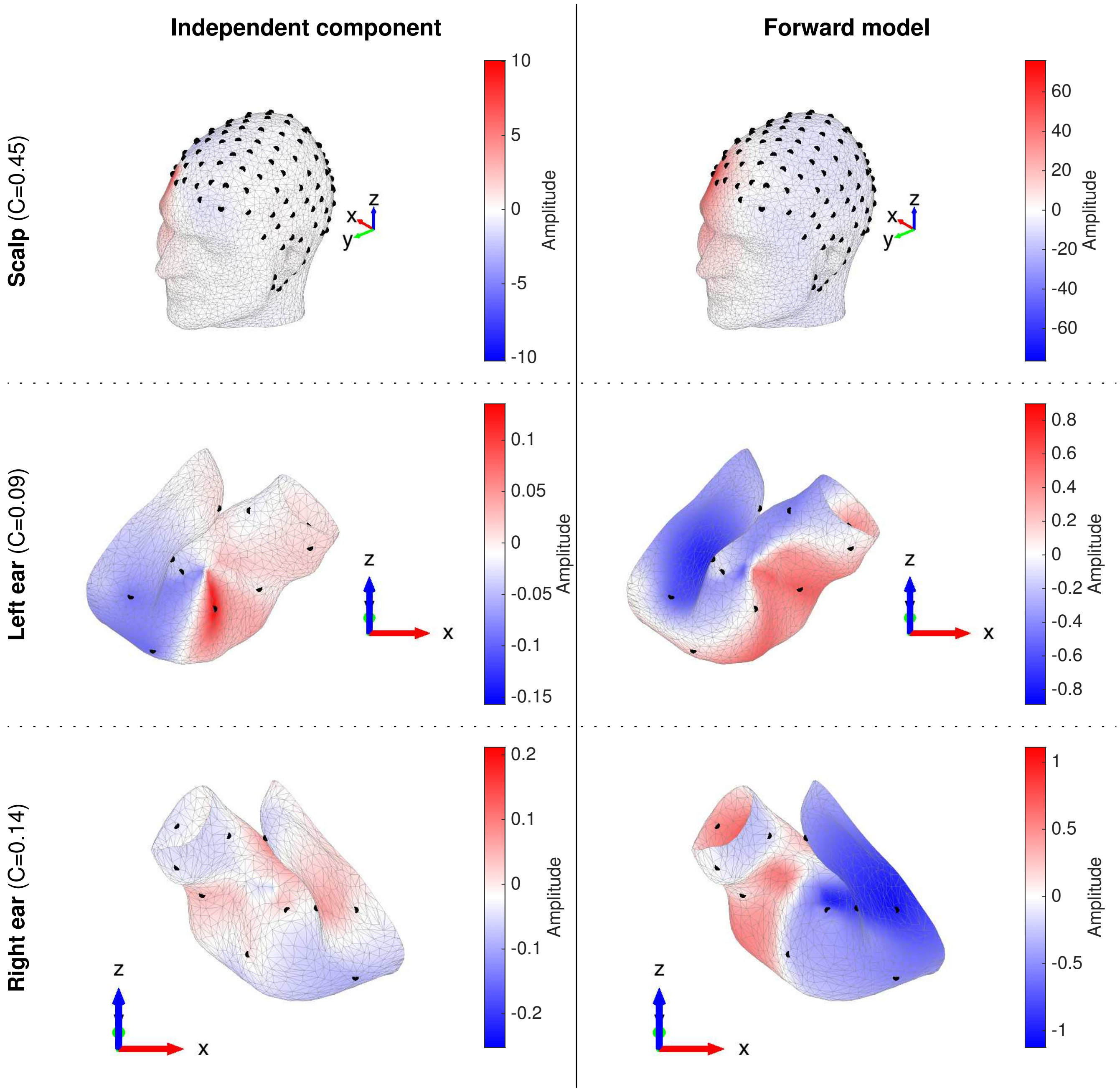

3 SUBJECT C

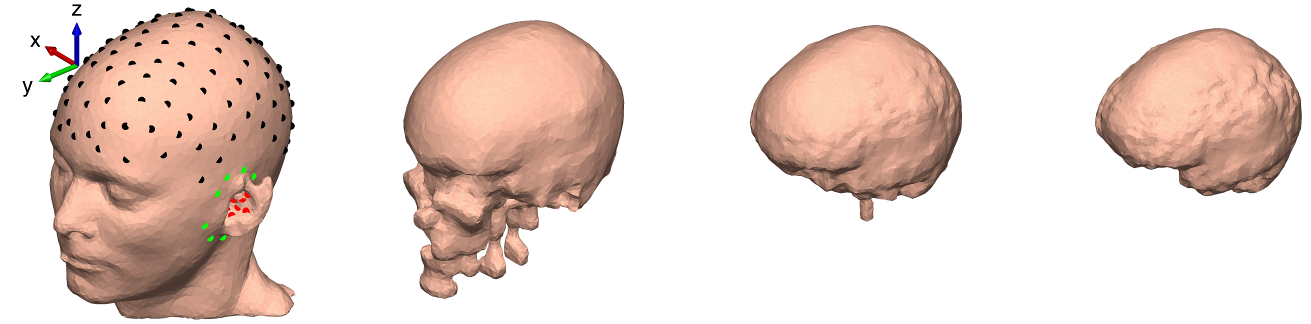

(a) Scalp (b) Outer skull (c) Inner skull (d) Brain

Figure S6. Headmodel mesh grids.

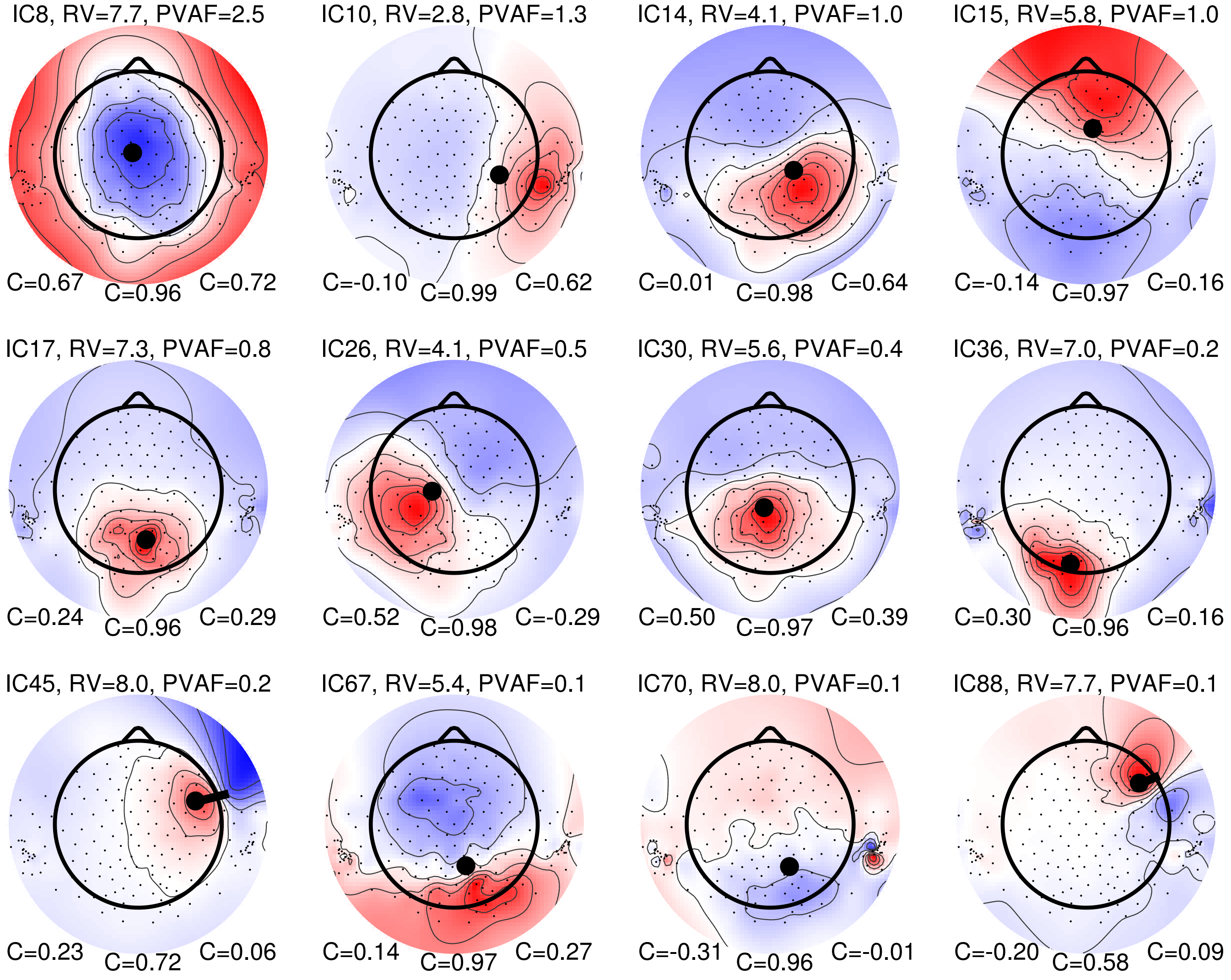

Figure S7. Topographic plots of the 12 independent components with the lowest residual variance.

Subject C - IC 8

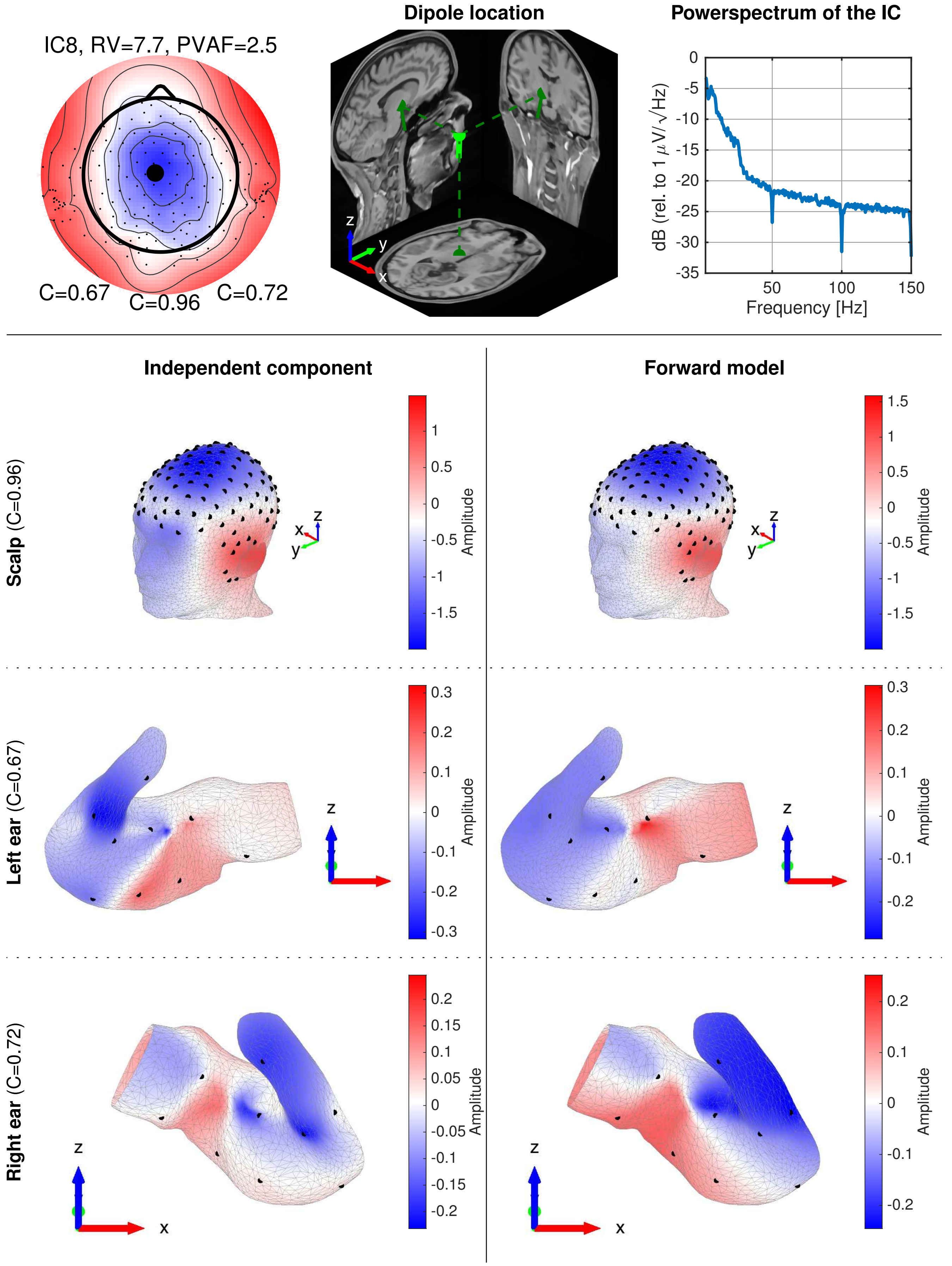

Subject C - IC 10

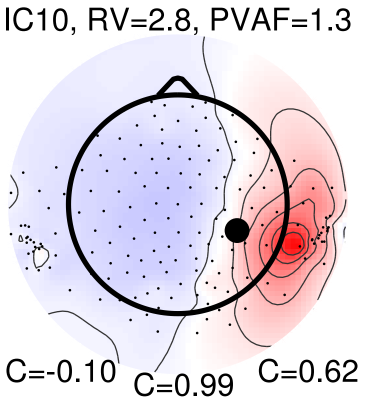

Dipole location

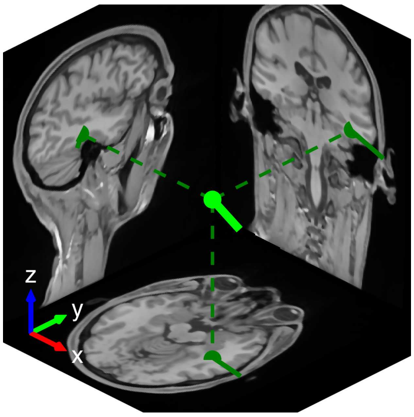

Powerspectrum of the IC

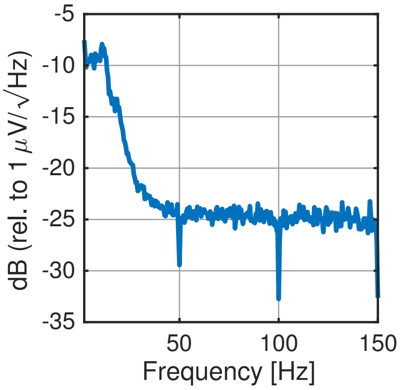

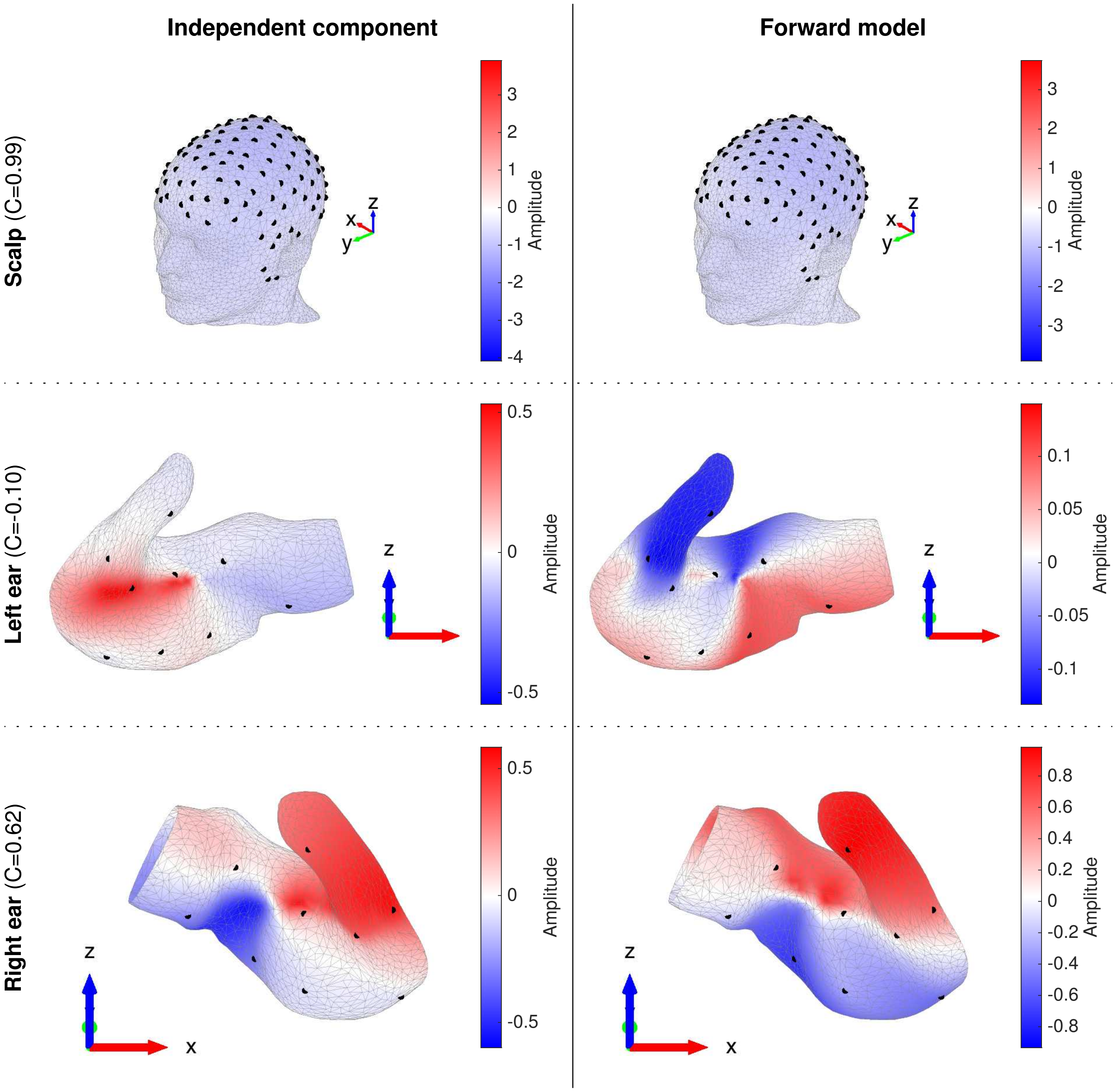

Subject C - IC 14

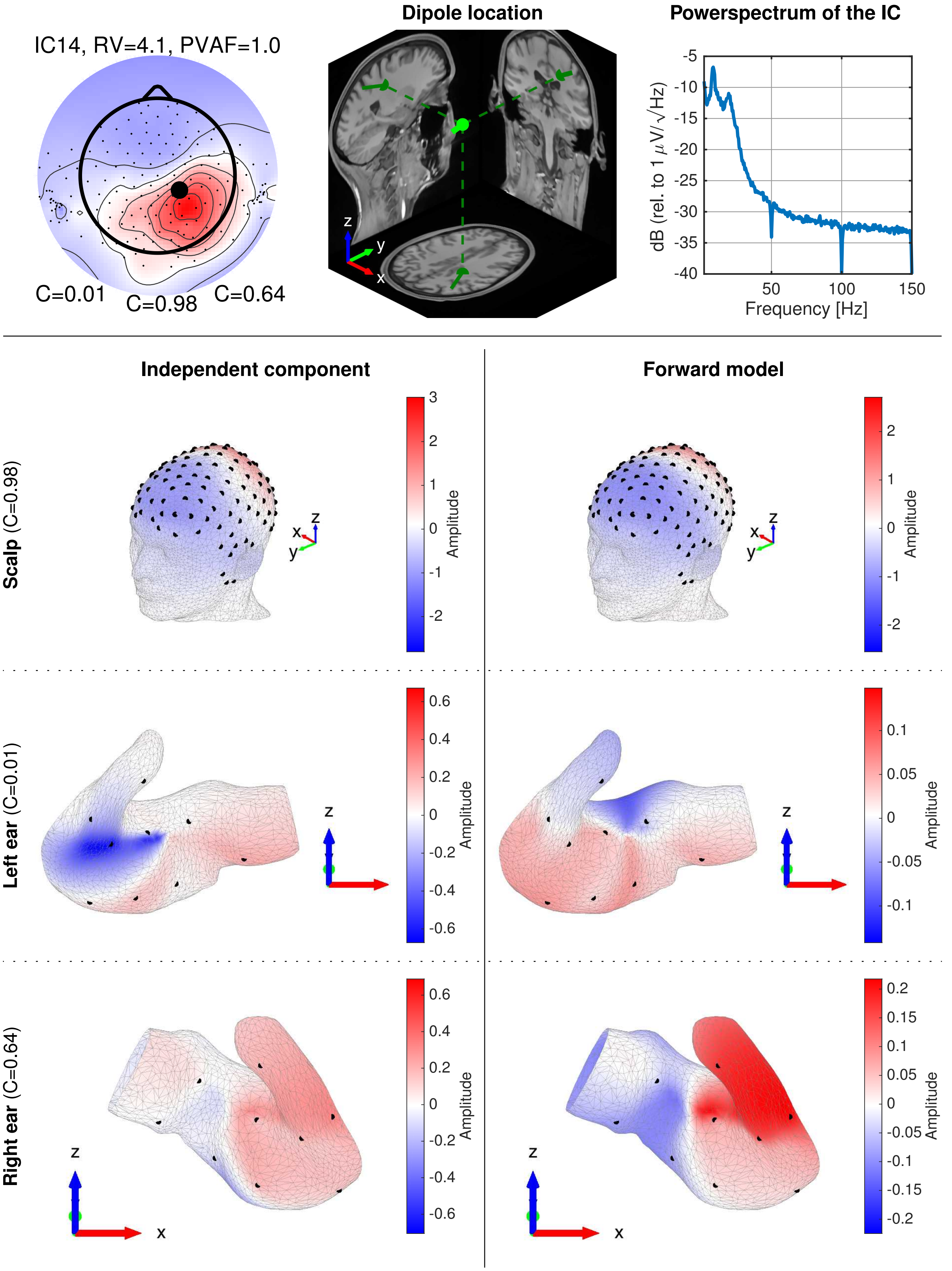

Subject C - IC 15

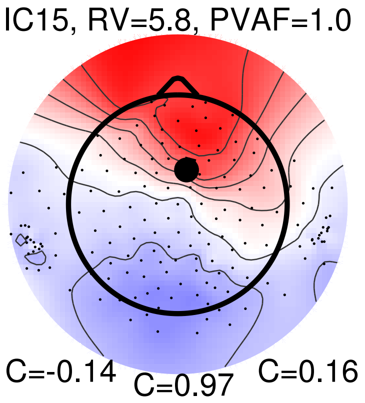

Dipole location

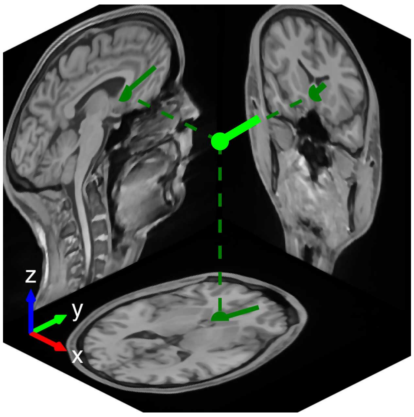

Powerspectrum of the IC

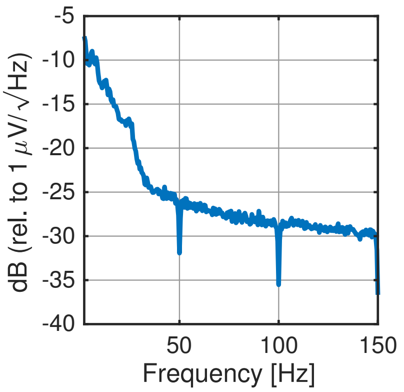

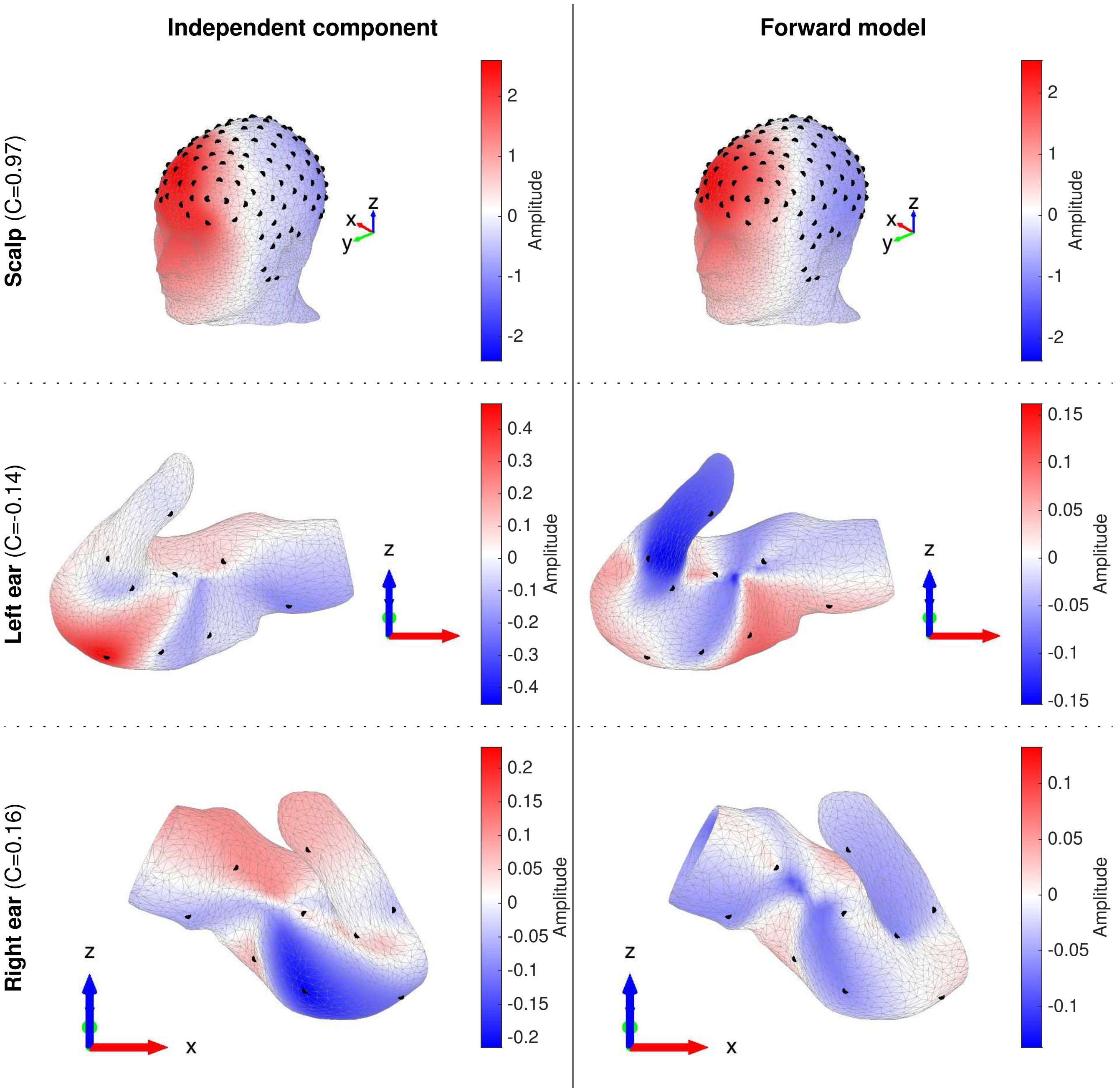

Subject C - IC 17

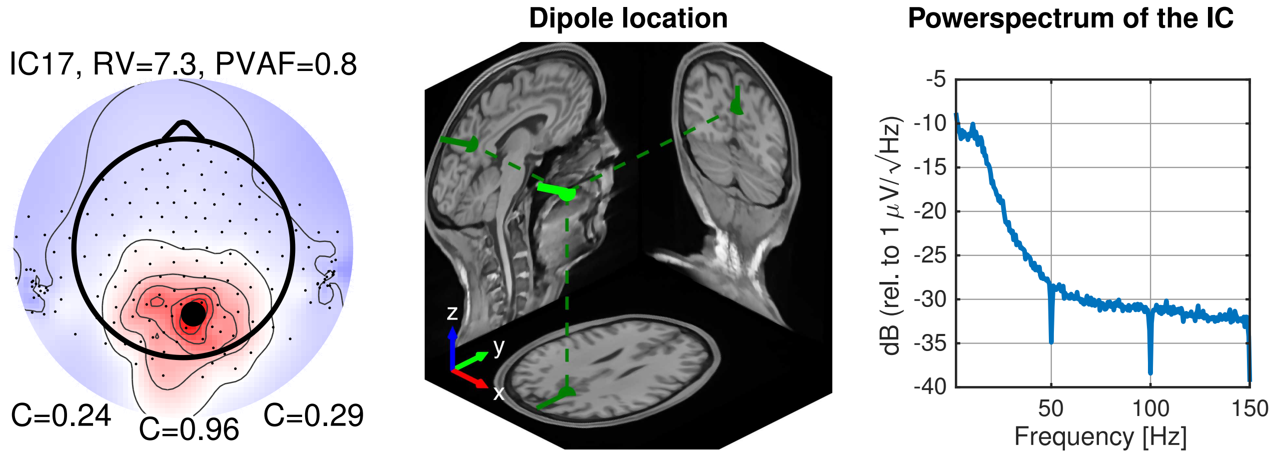

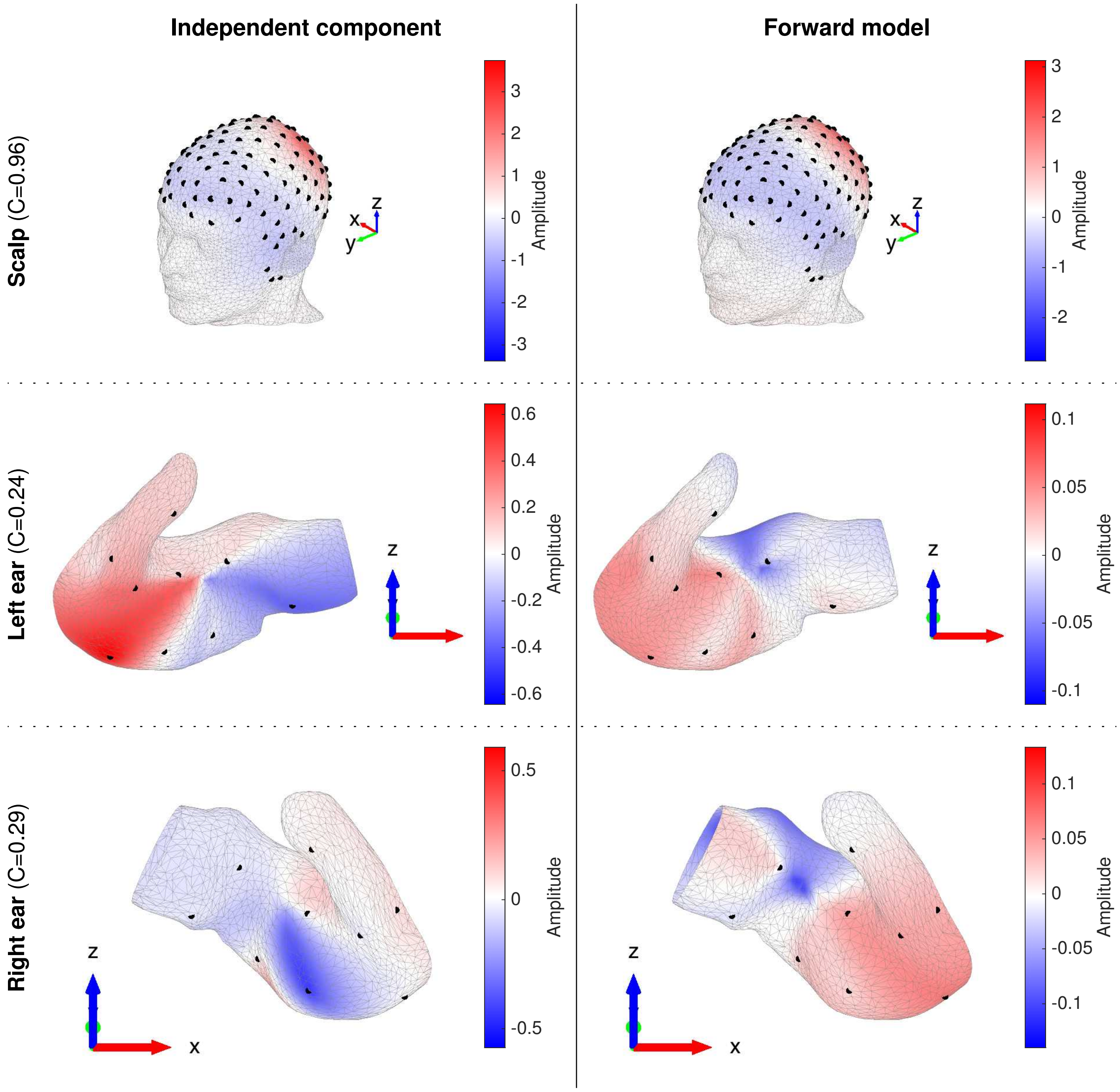

Subject C - IC 26

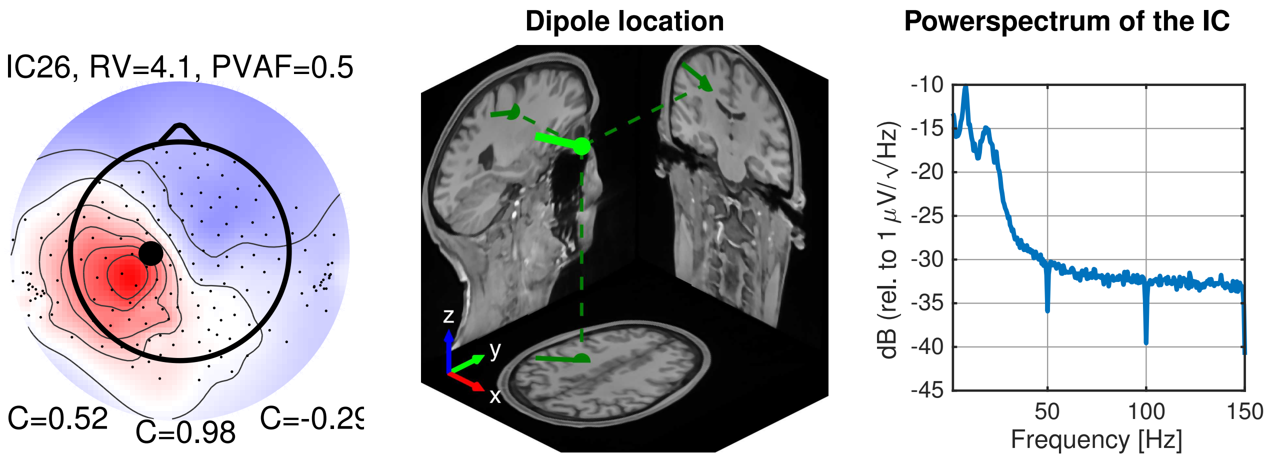

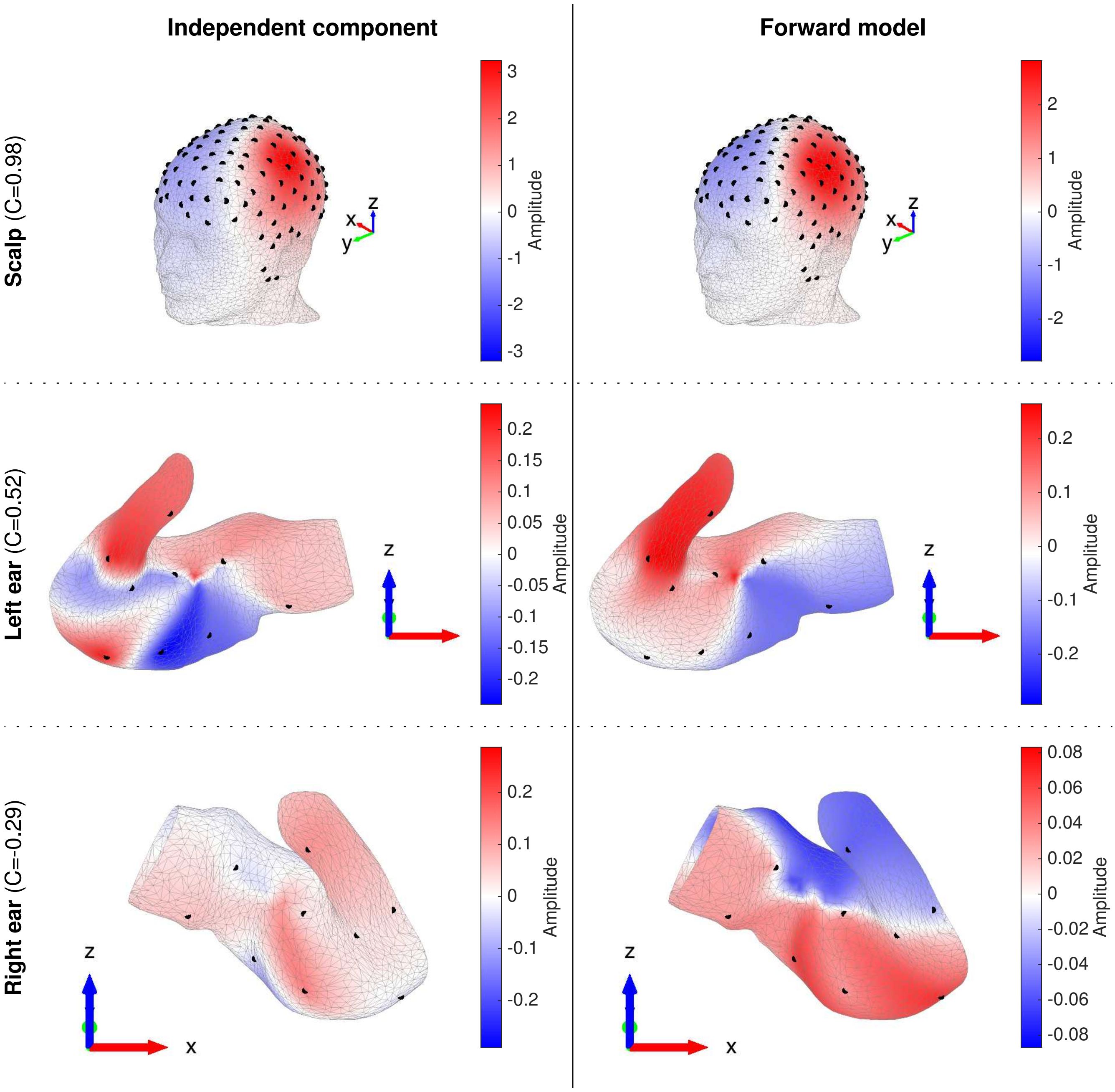

Subject C - IC 30

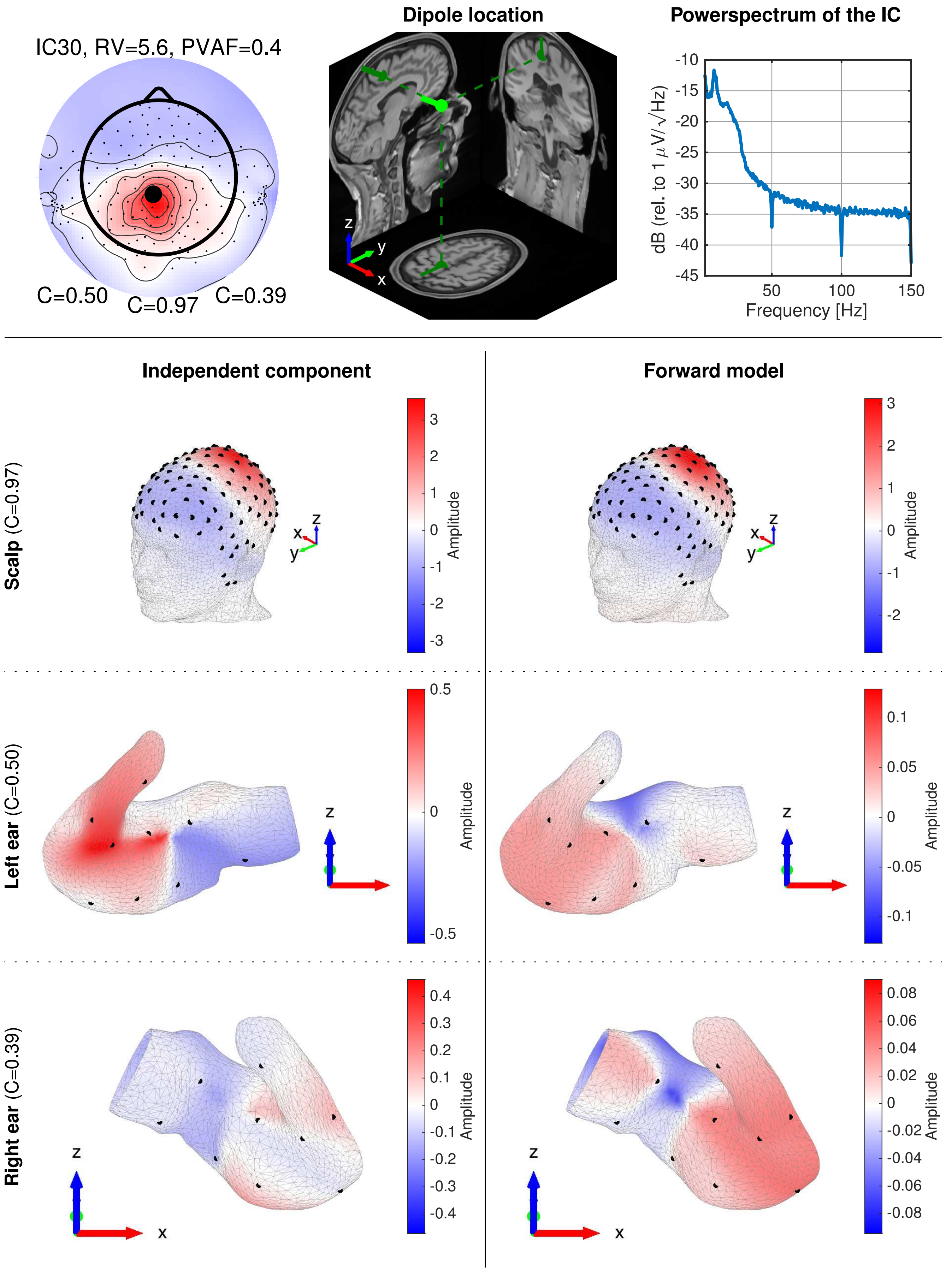

Subject C - IC 36

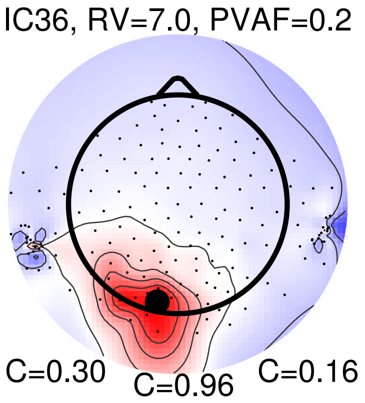

Dipole location

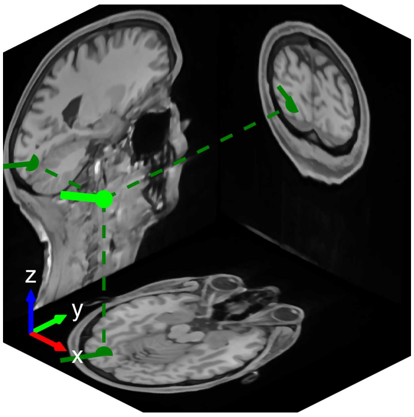

Powerspectrum of the IC

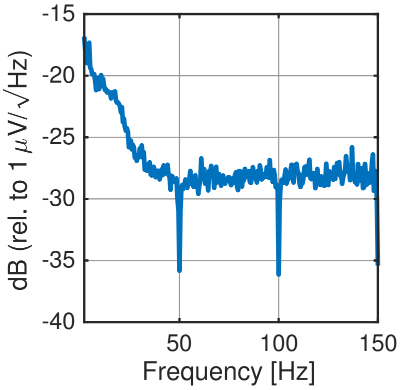

Independent component | **Forward model**

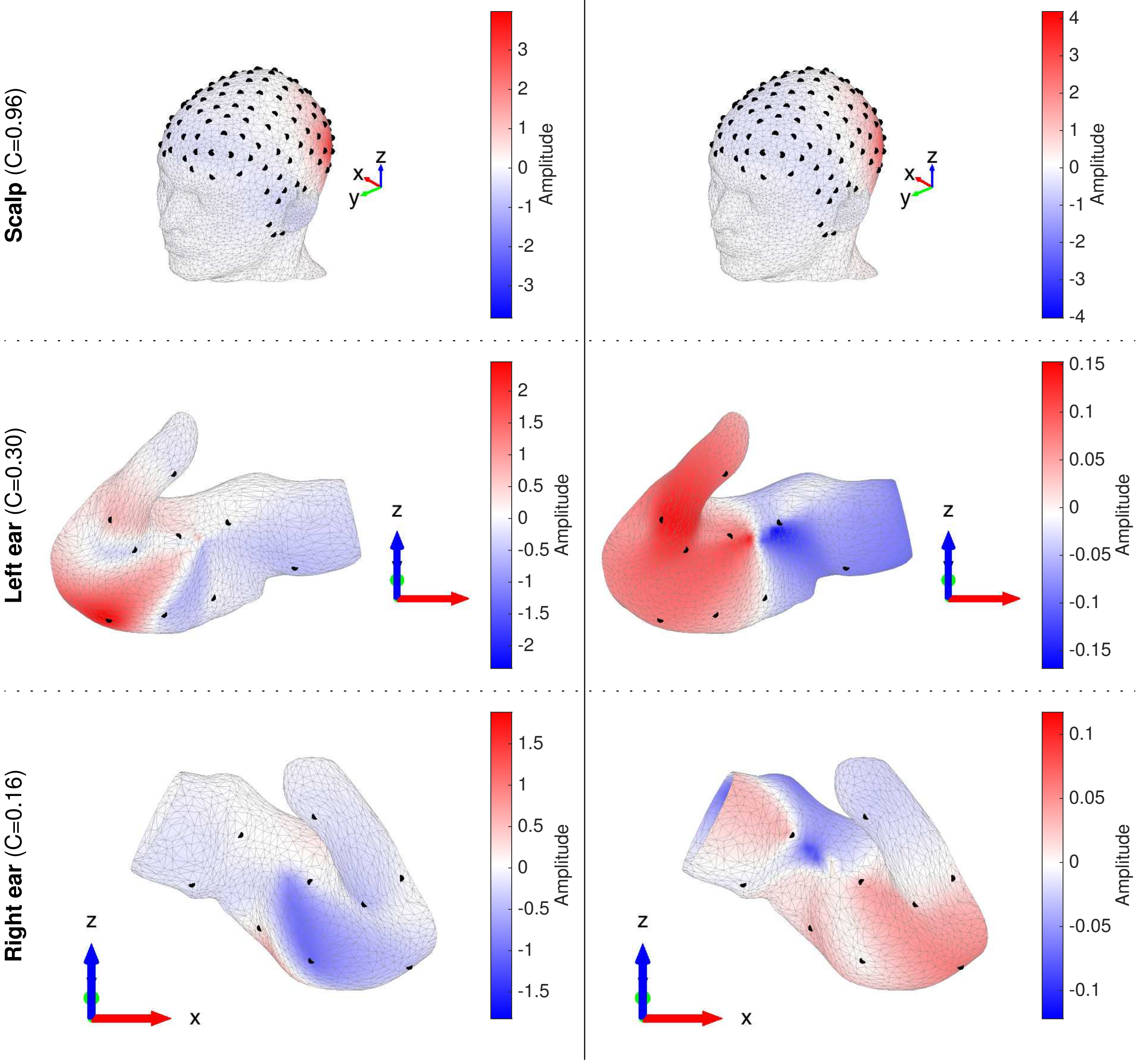

Subject C - IC 45

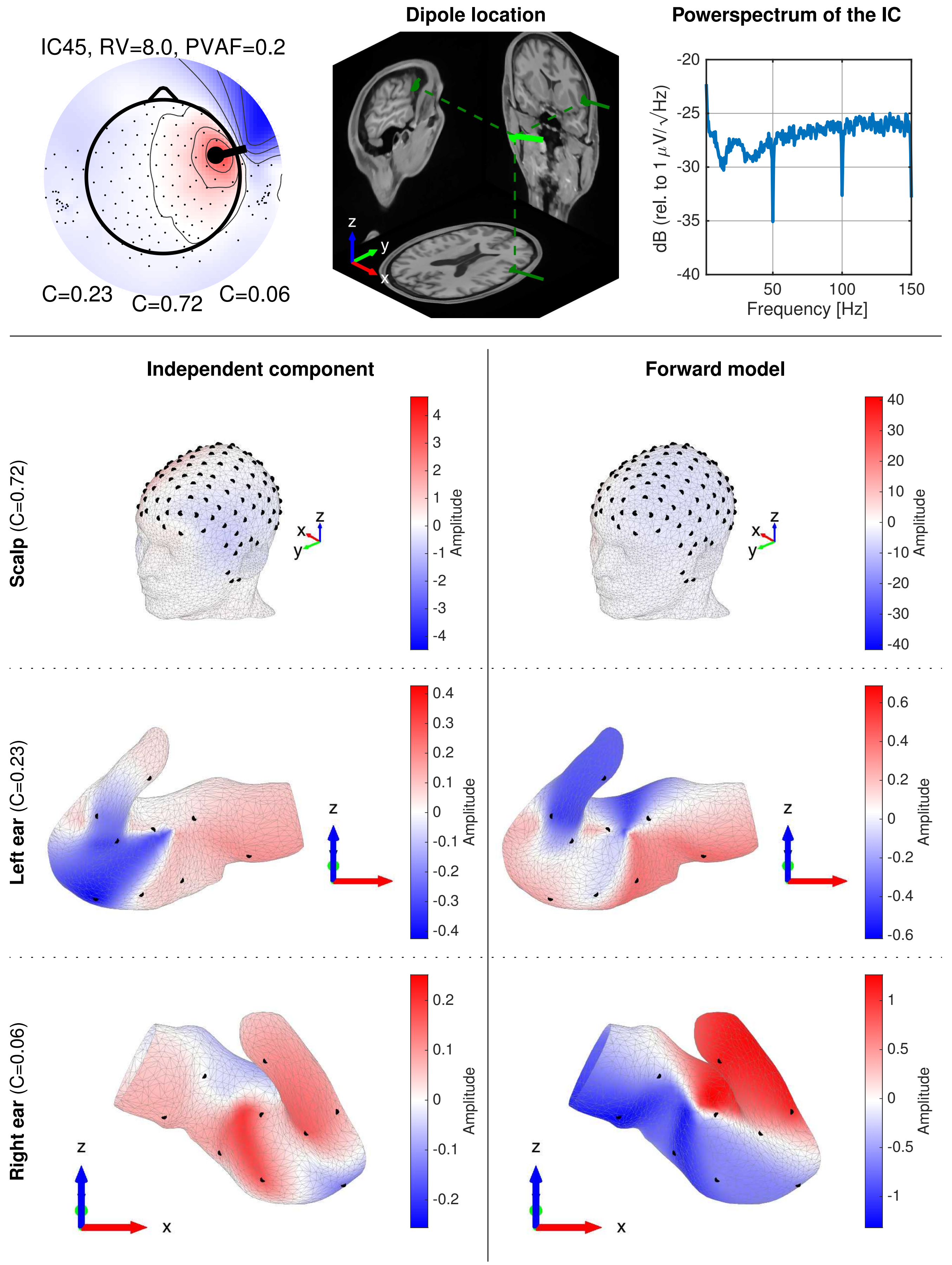

Subject C - IC 67

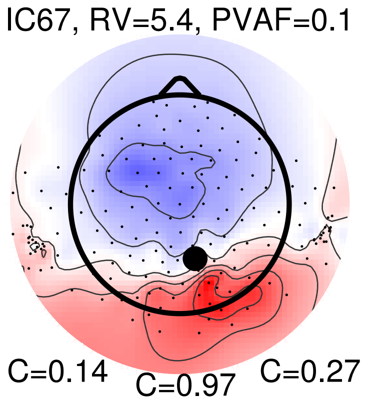

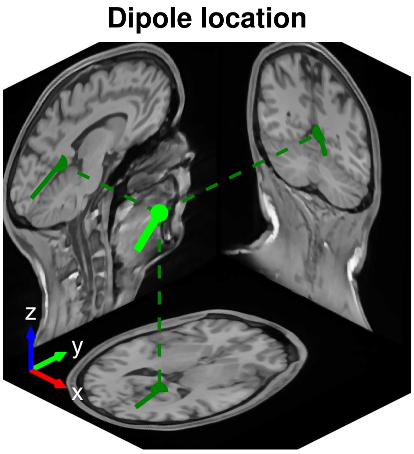

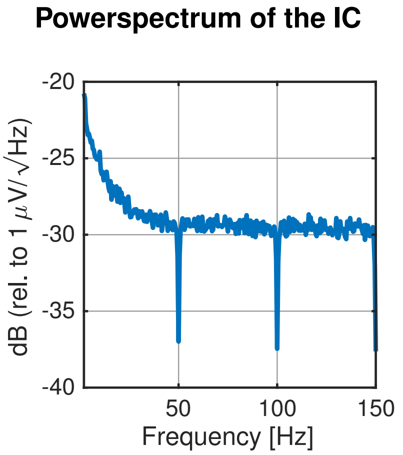

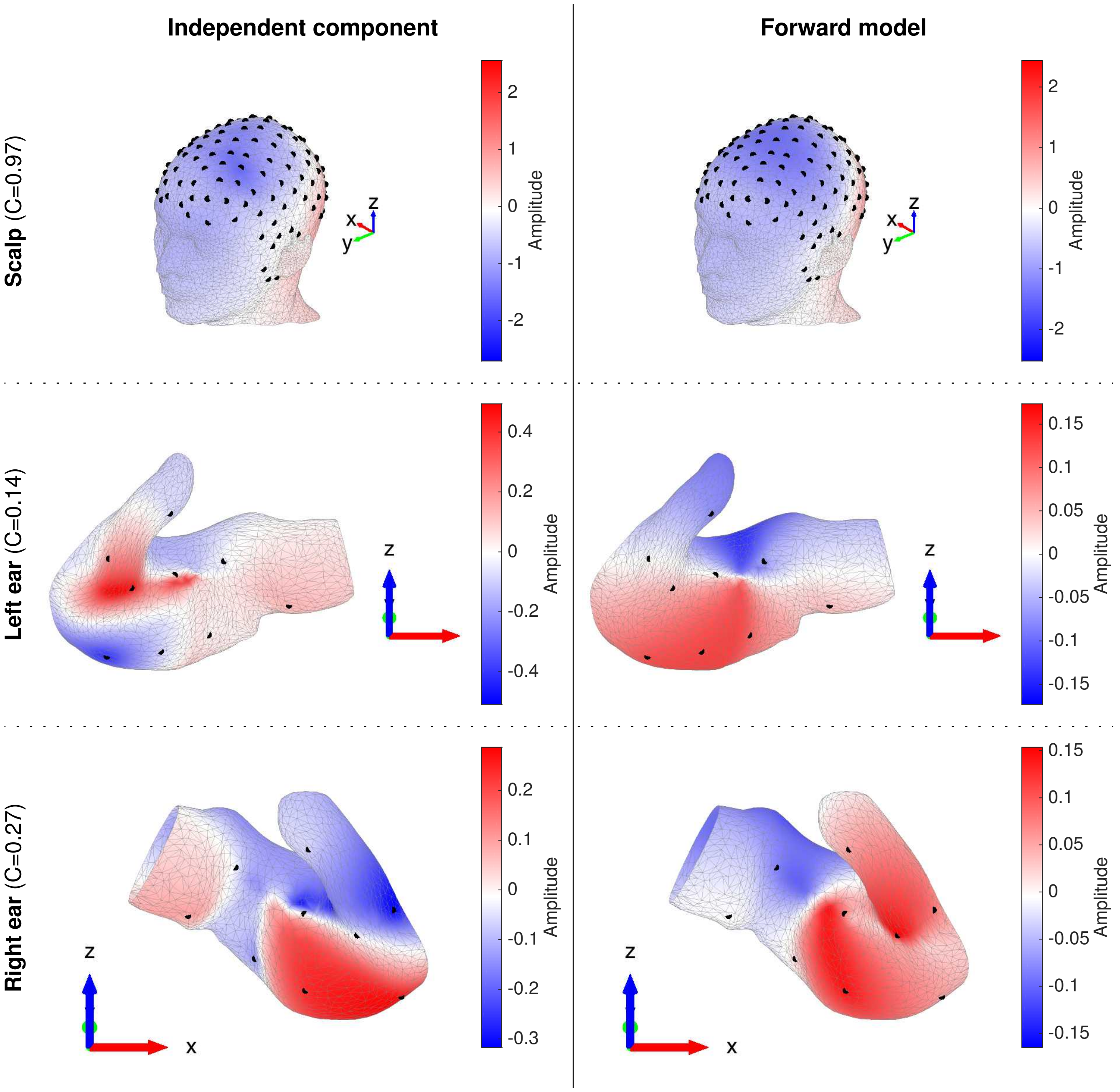

Subject C - IC 70

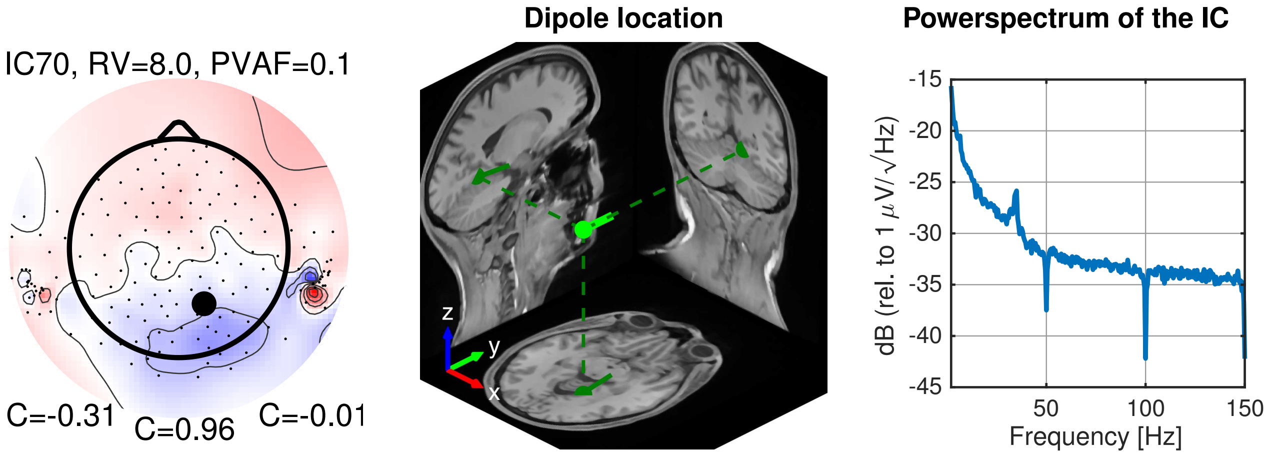

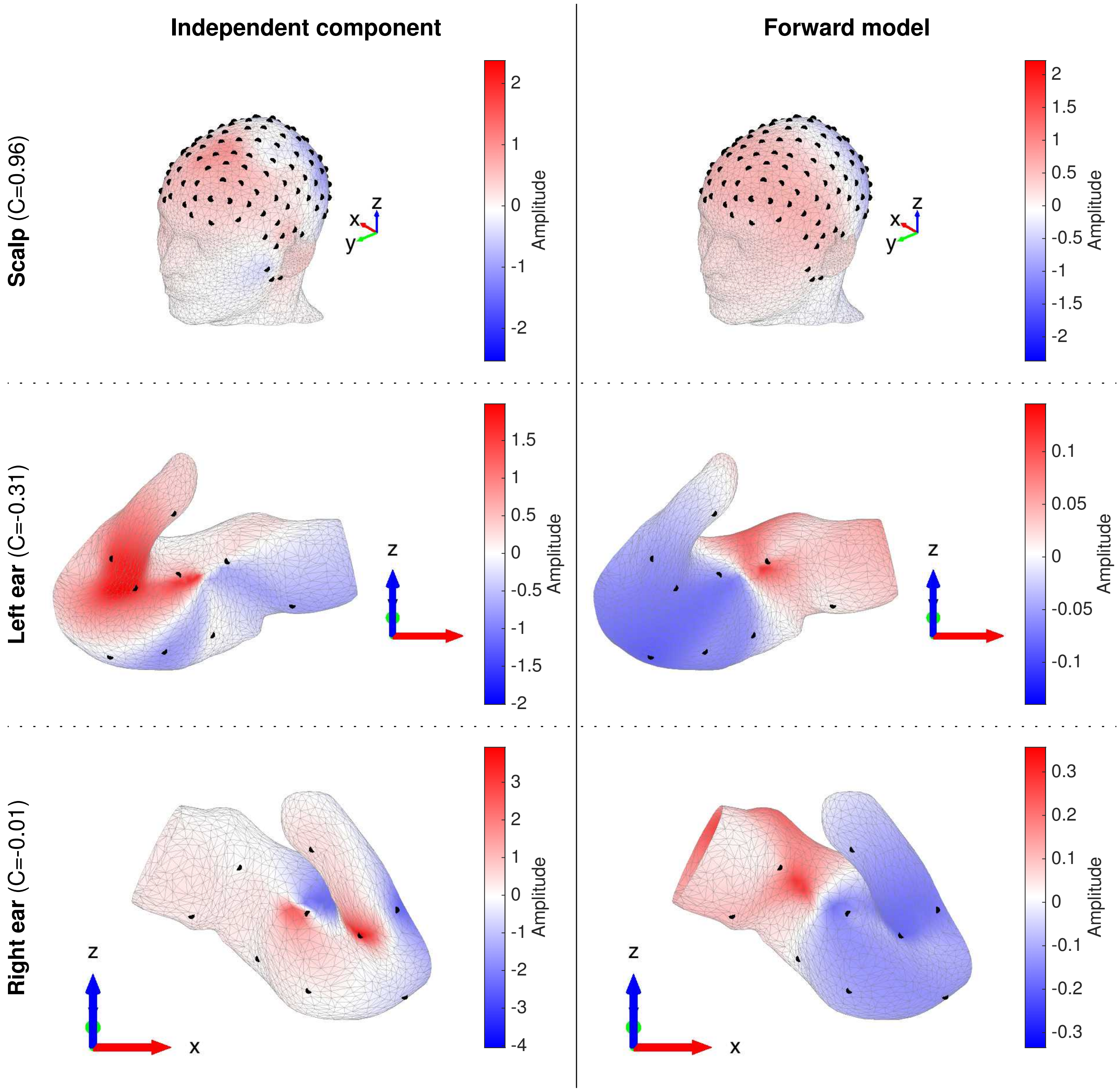

Subject C - IC 88

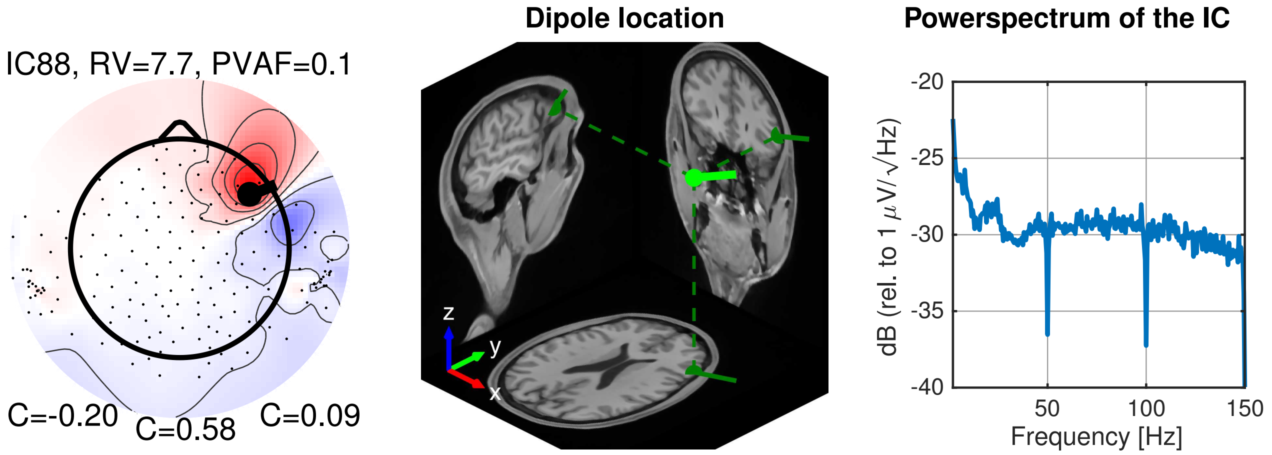

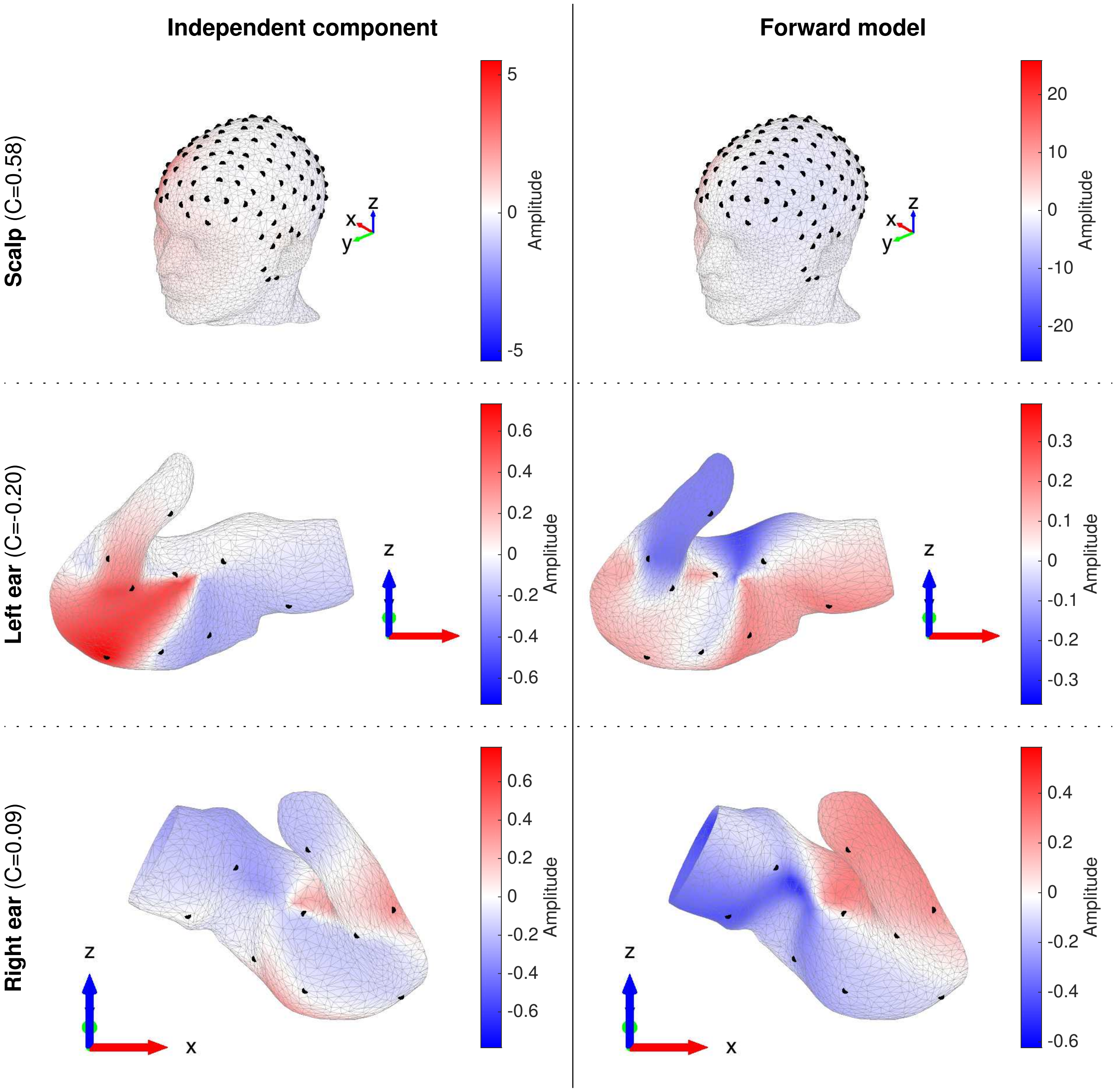

4 SUBJECT D

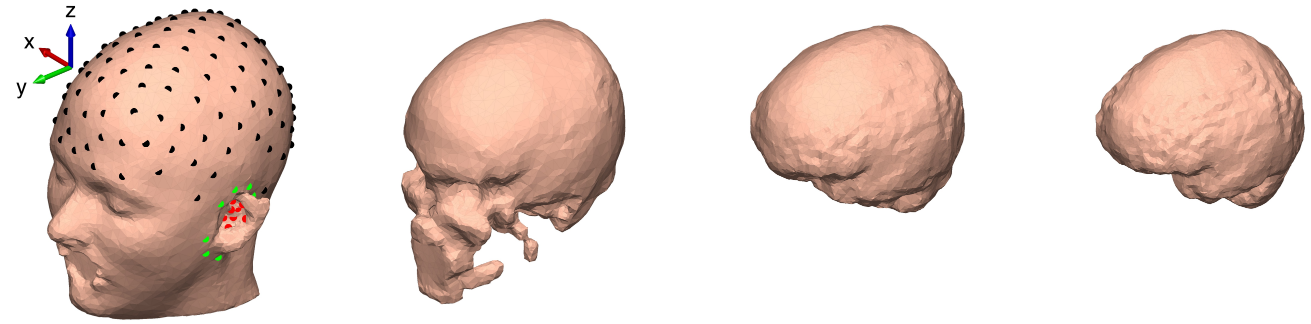

(a) Scalp (b) Outer skull (c) Inner skull (d) Brain

Figure S8. Headmodel mesh grids.

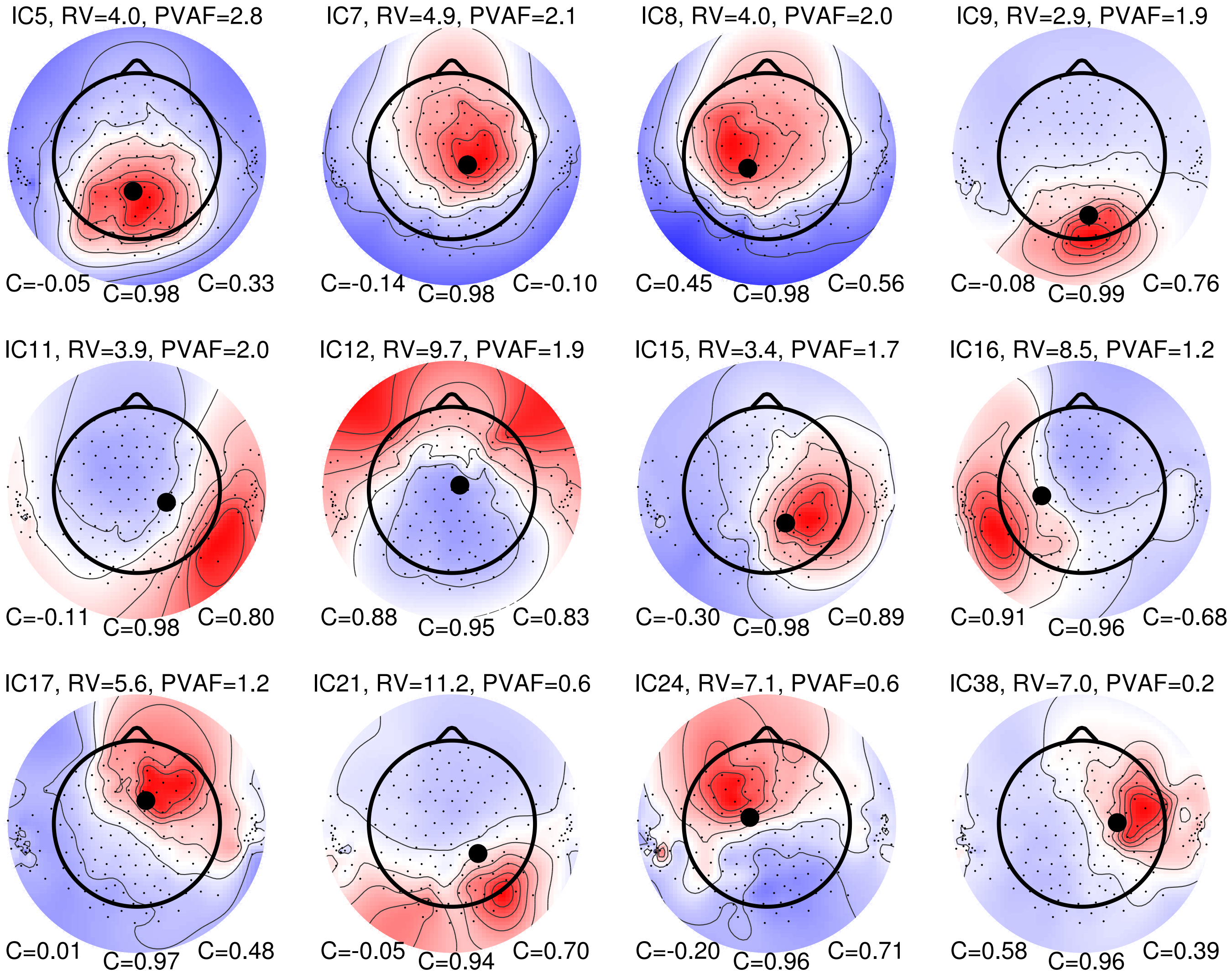

Figure S9. Topographic plots of the 12 independent components with the lowest residual variance.

Subject D - IC 5

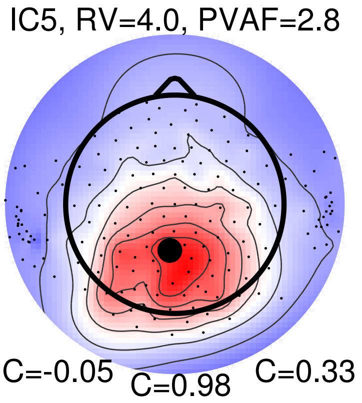

Dipole location

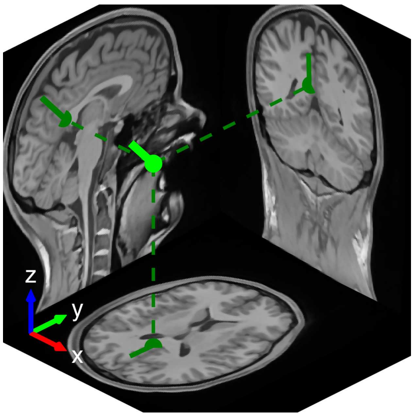

Powerspectrum of the IC

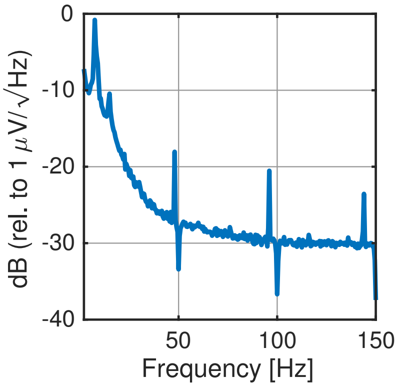

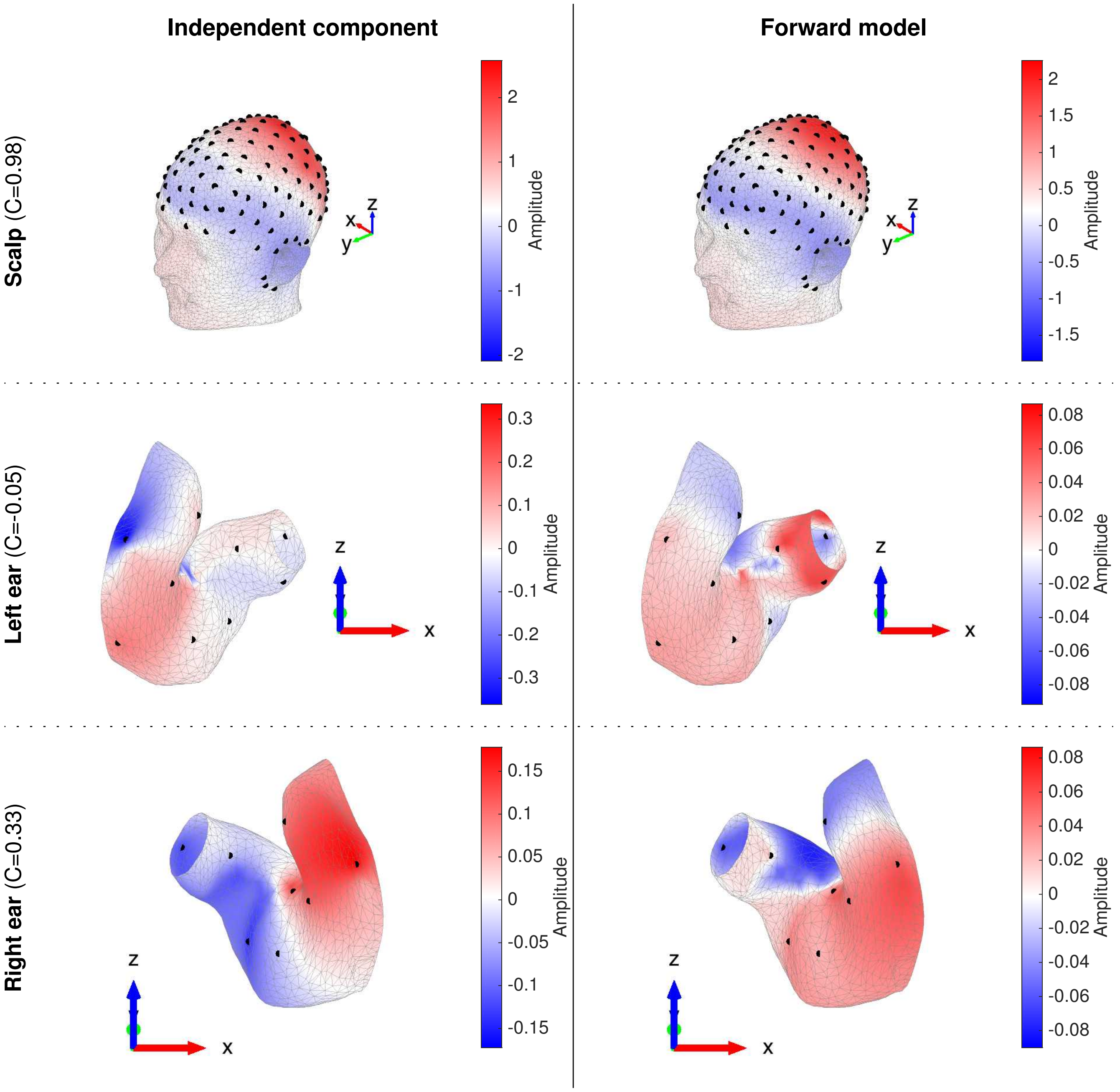

Subject D - IC 7

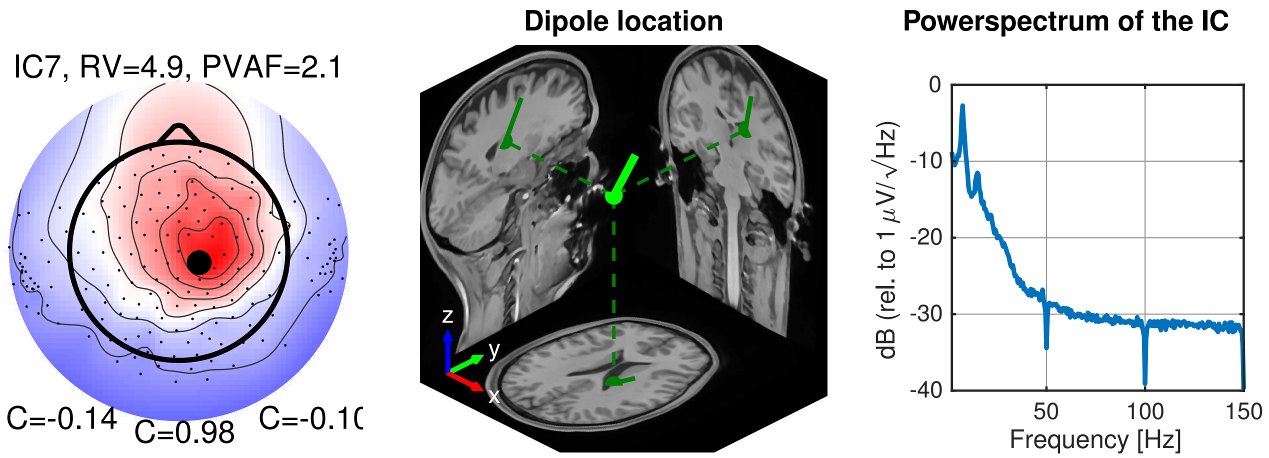

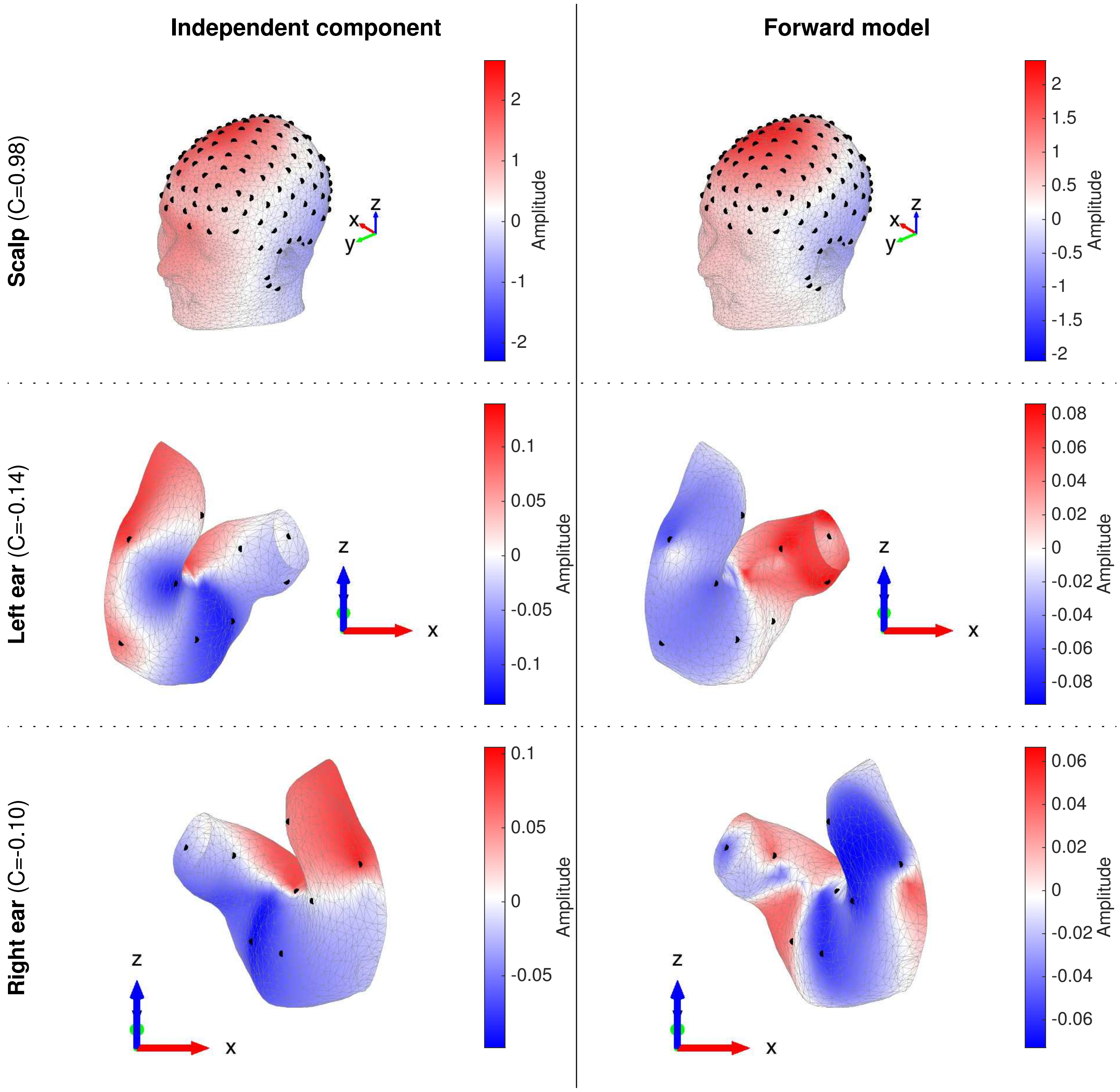

Subject D - IC 8

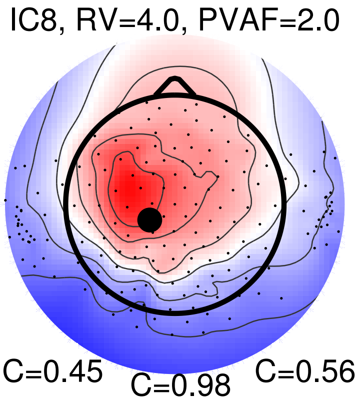

Dipole location

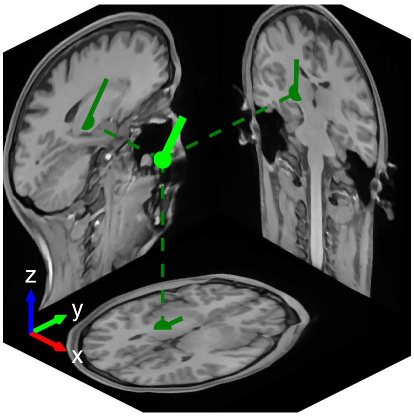

Powerspectrum of the IC

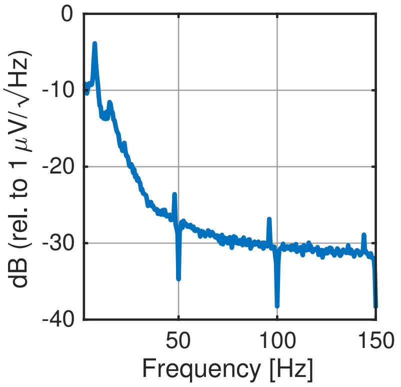

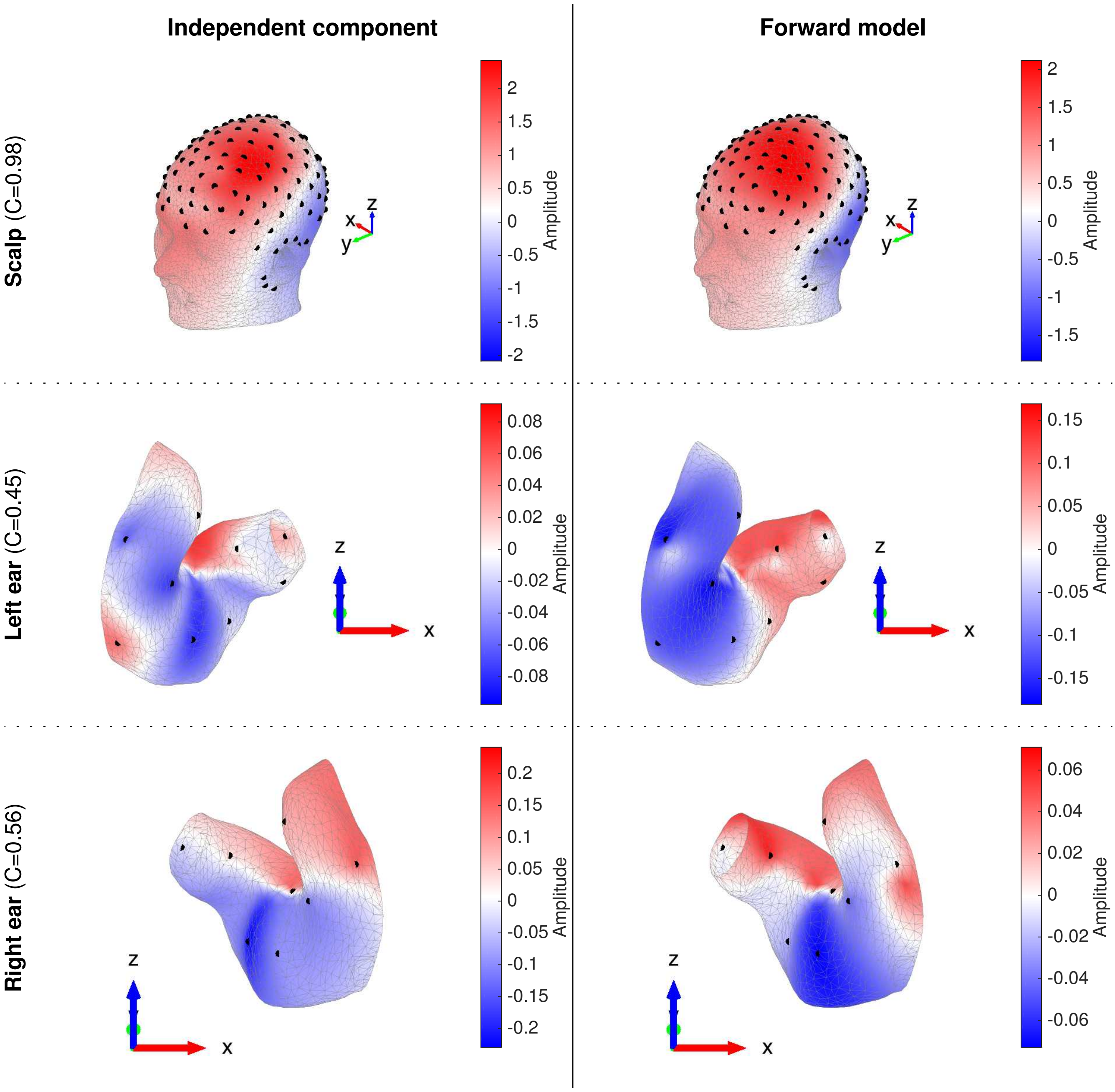

Subject D - IC 9

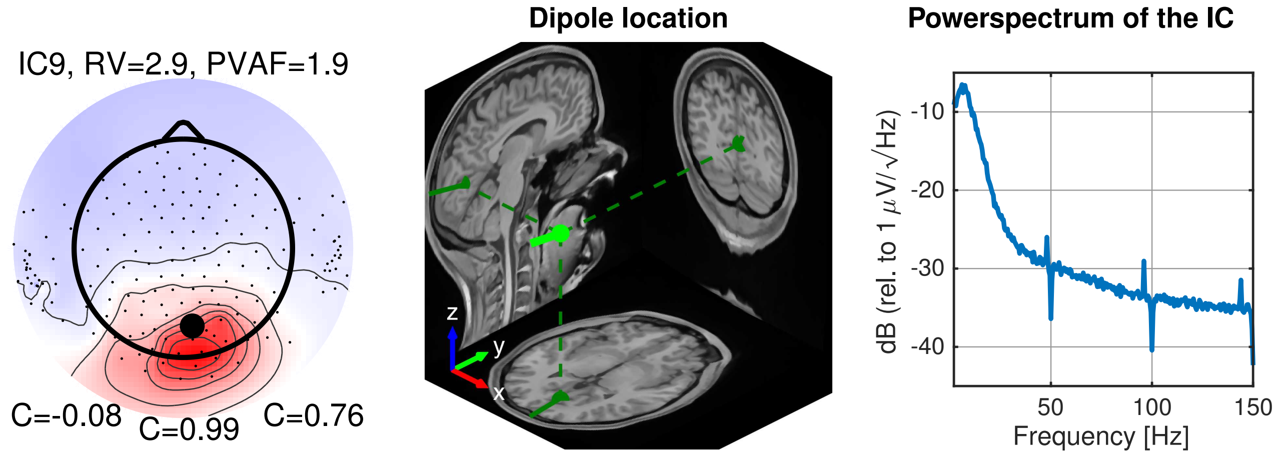

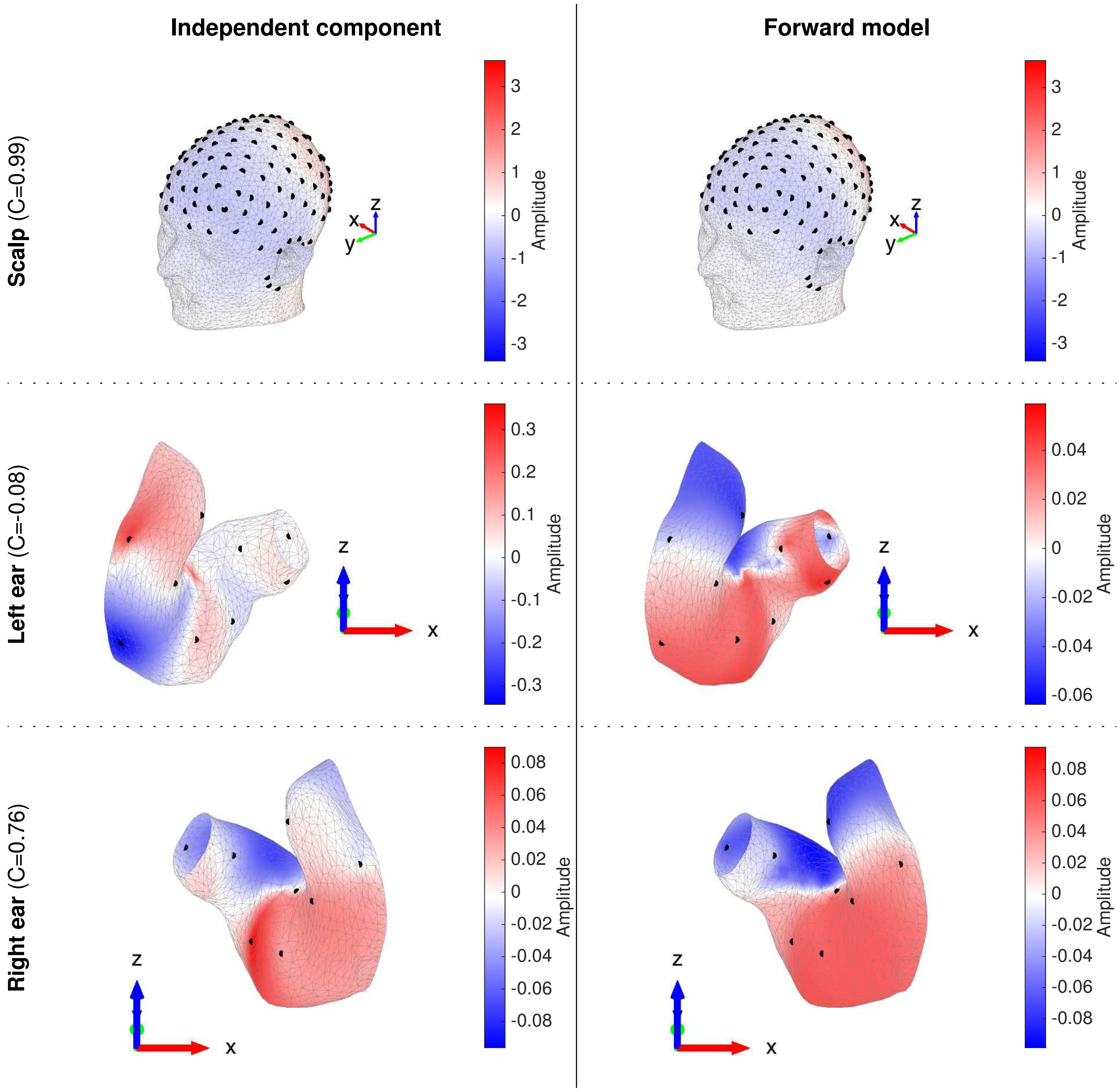

Subject D - IC 11

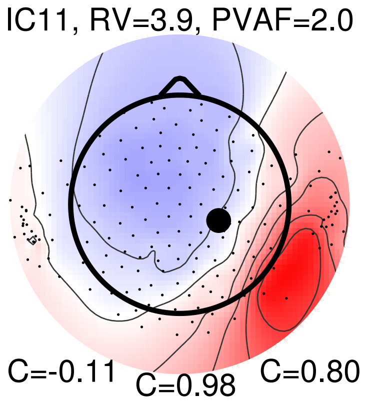

Dipole location

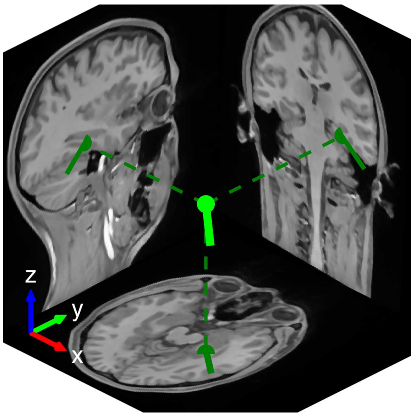

Powerspectrum of the IC

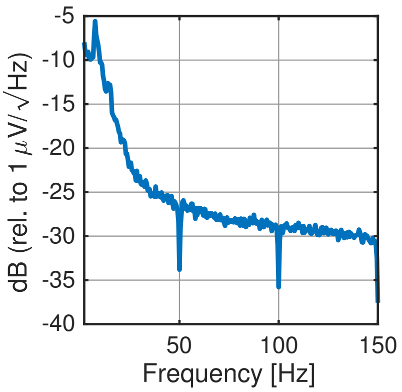

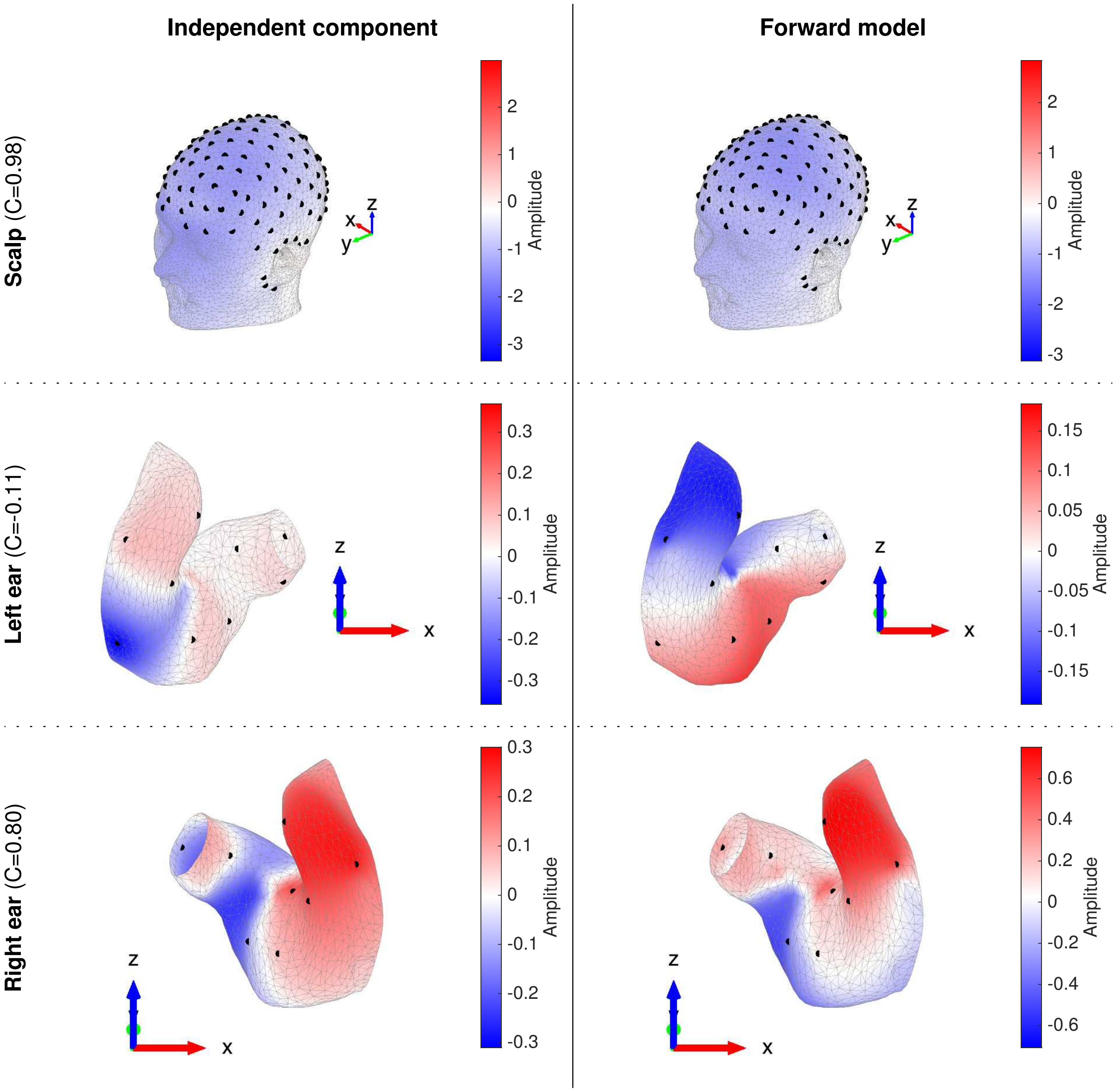

Subject D - IC 12

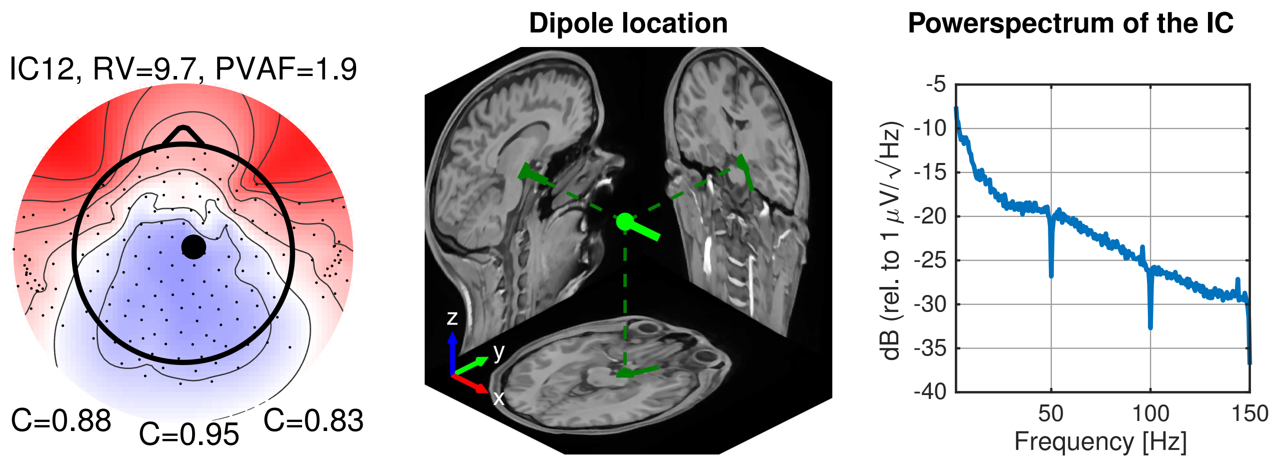

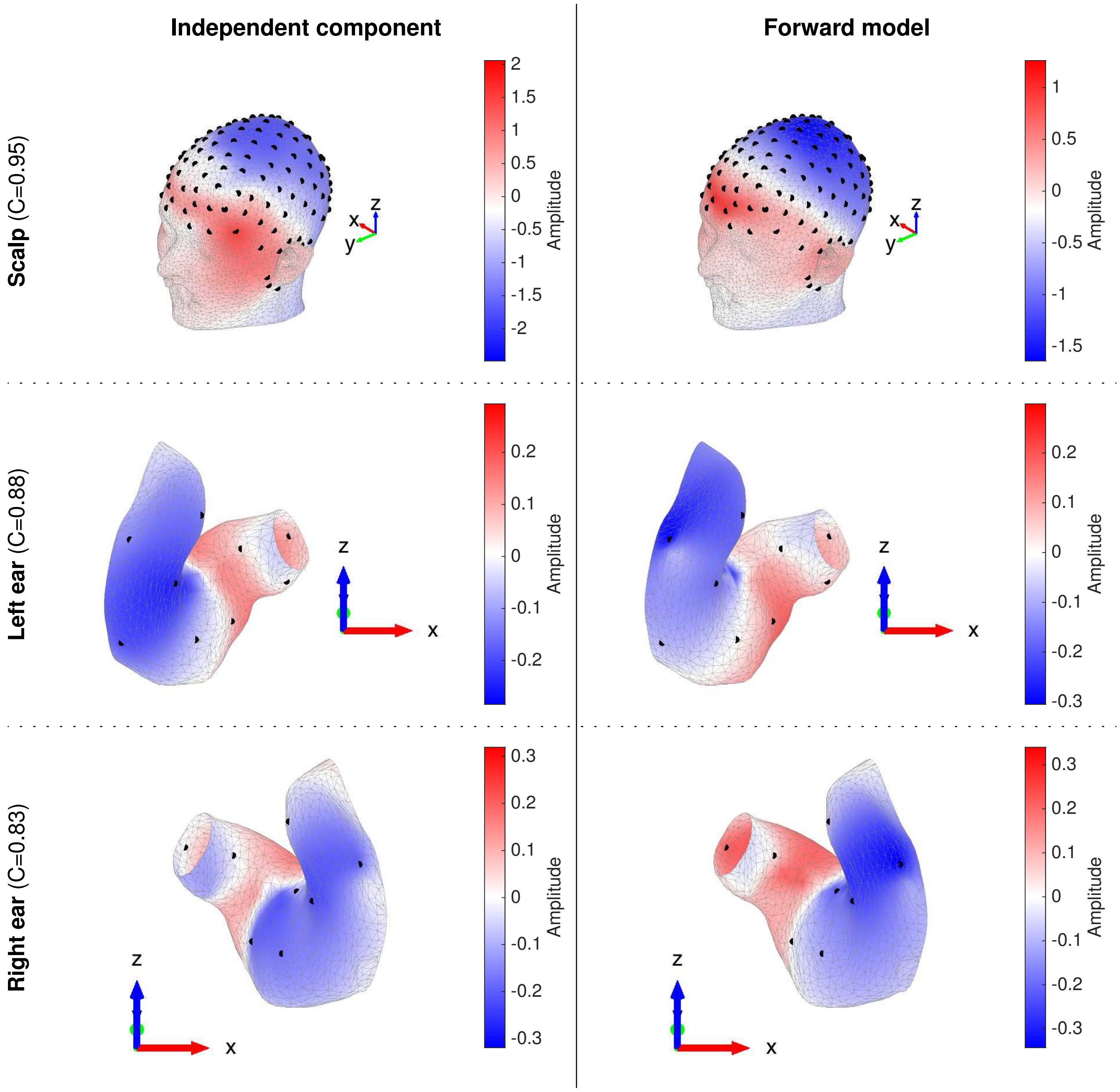

Subject D - IC 15

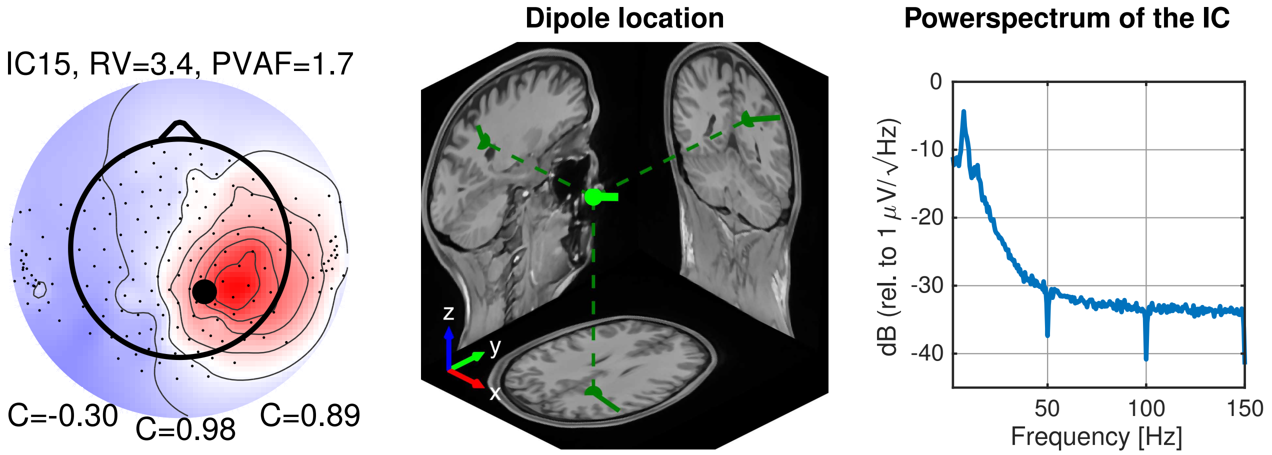

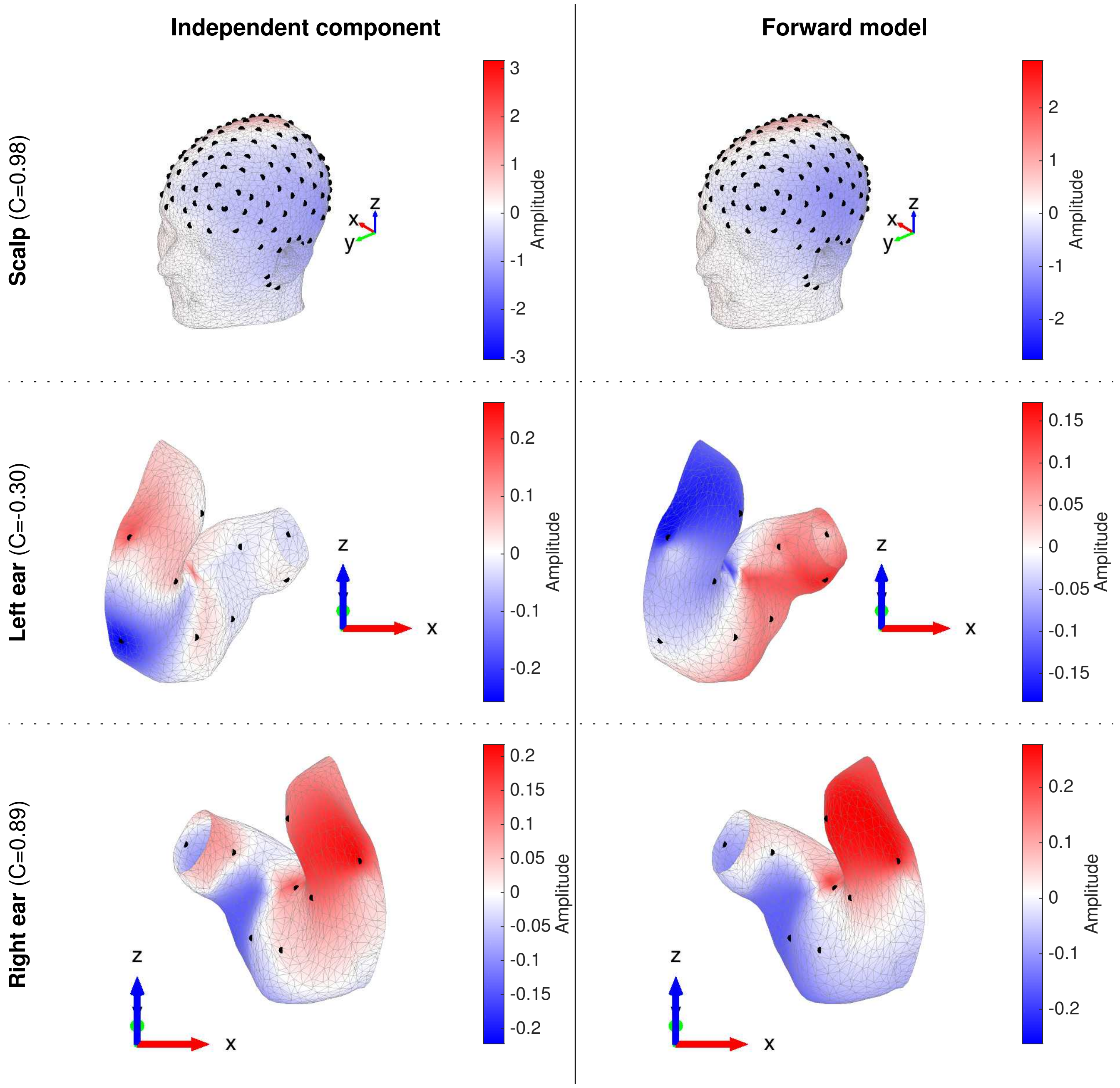

Subject D - IC 16

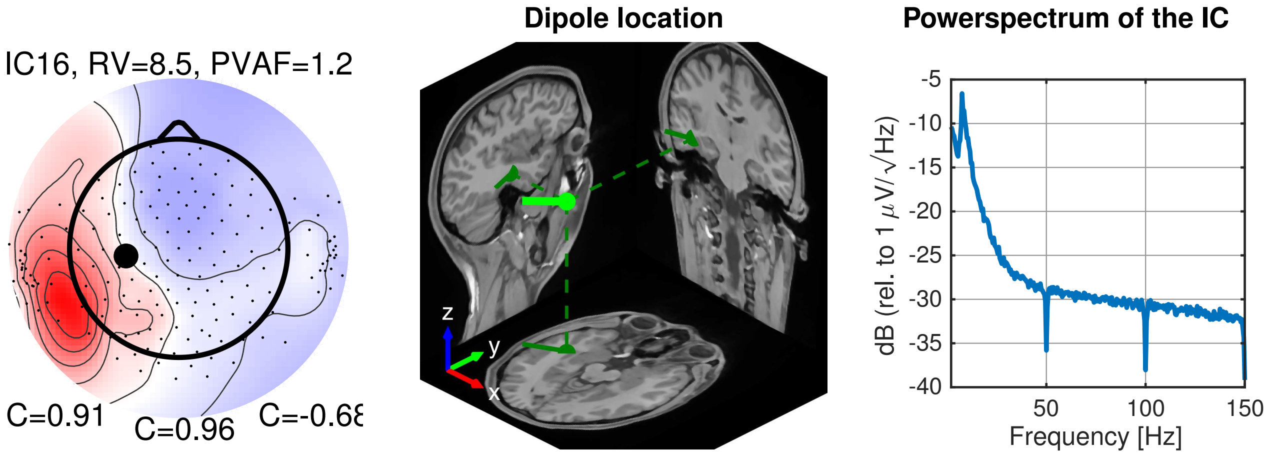

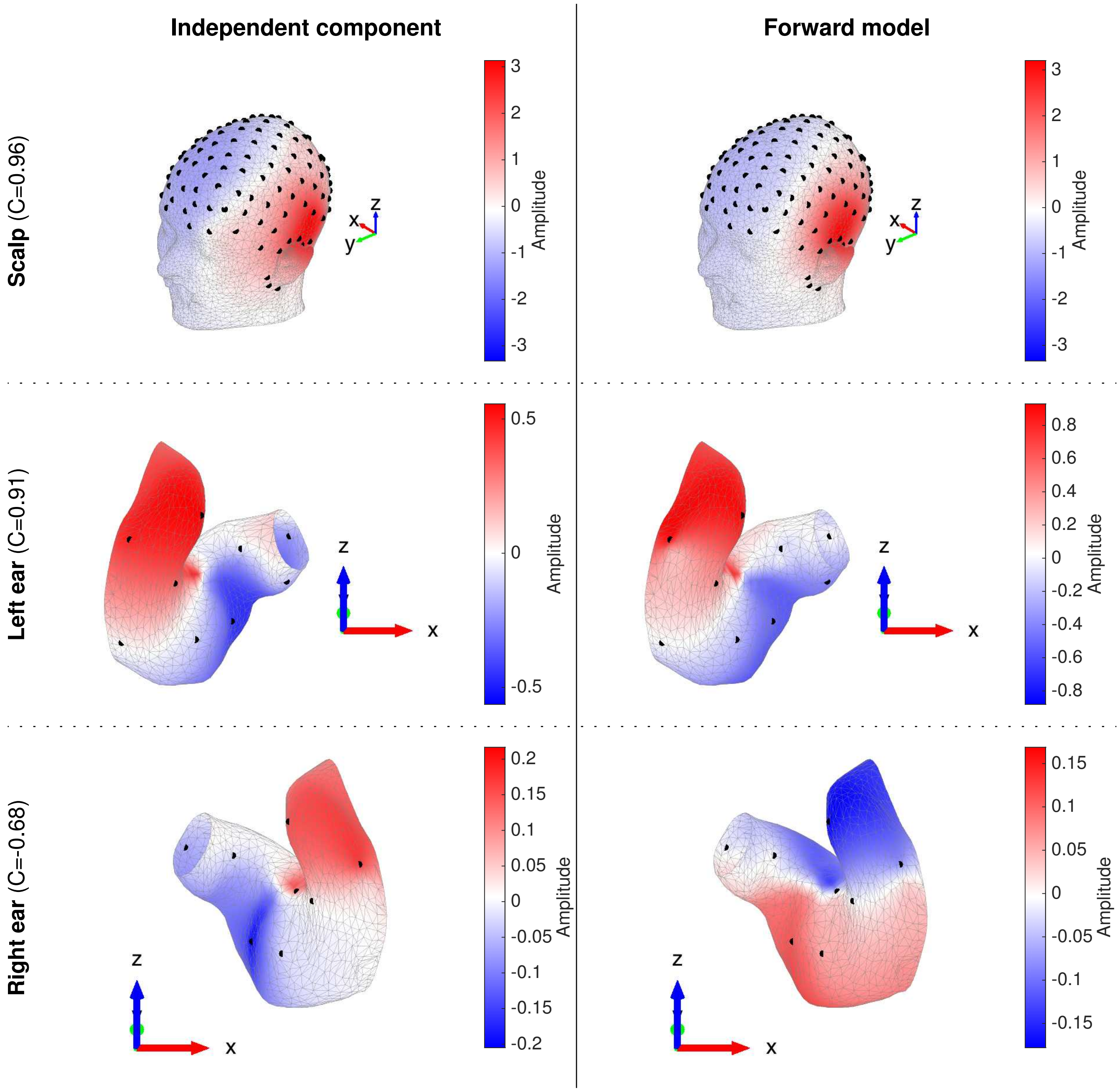

Subject D - IC 17

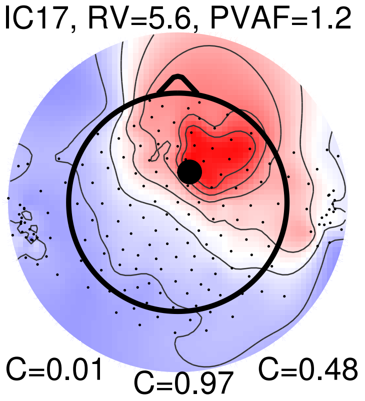

Dipole location

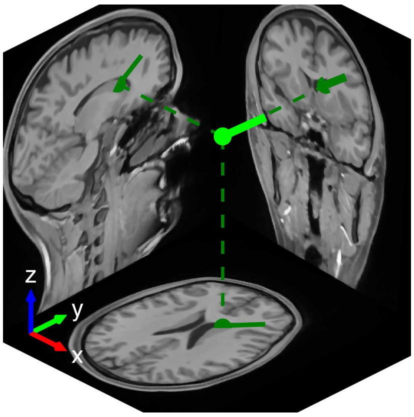

Powerspectrum of the IC

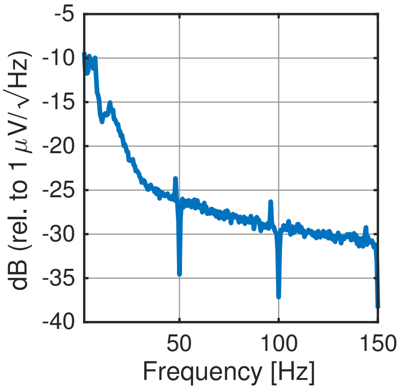

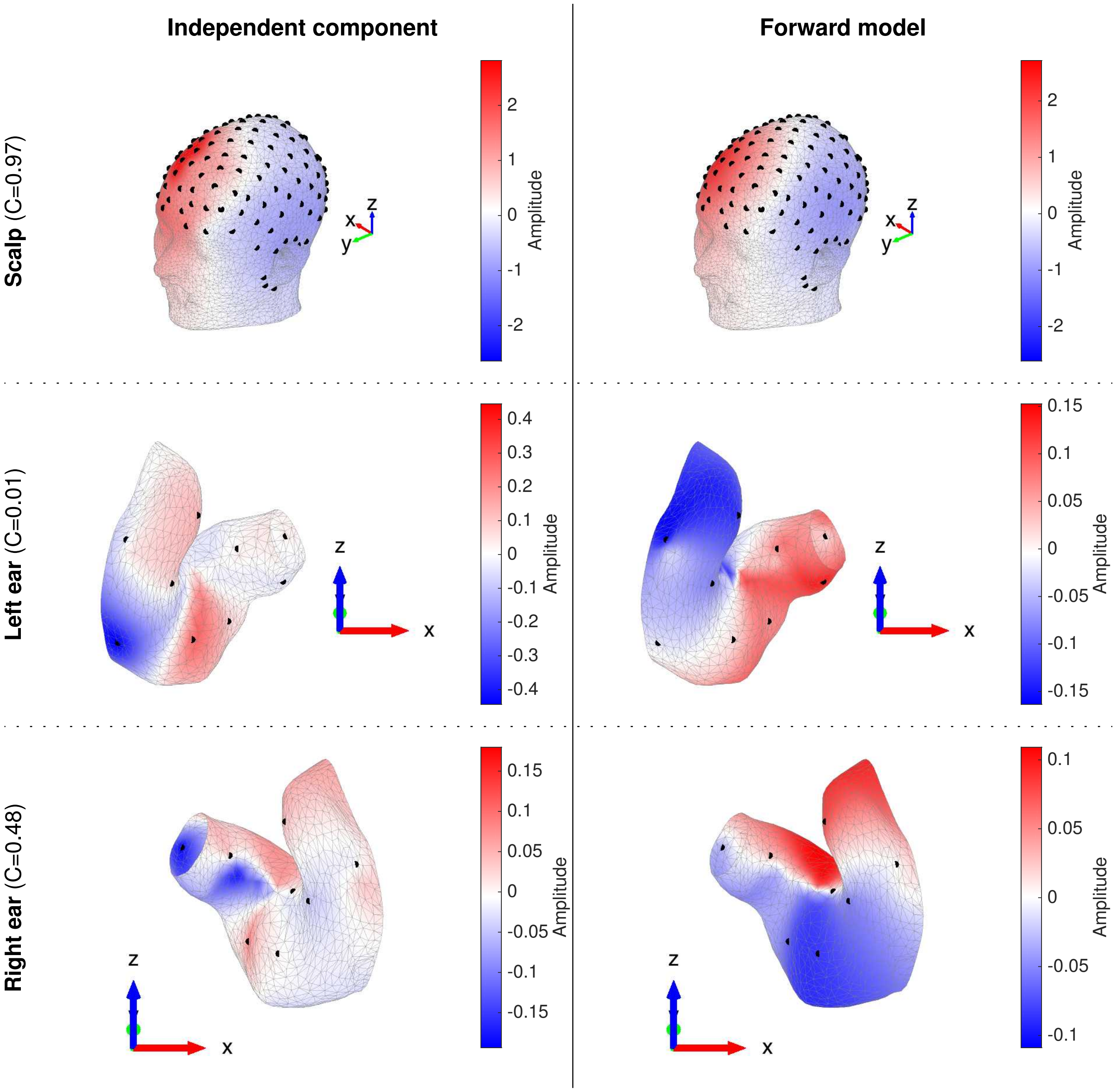

Subject D - IC 21

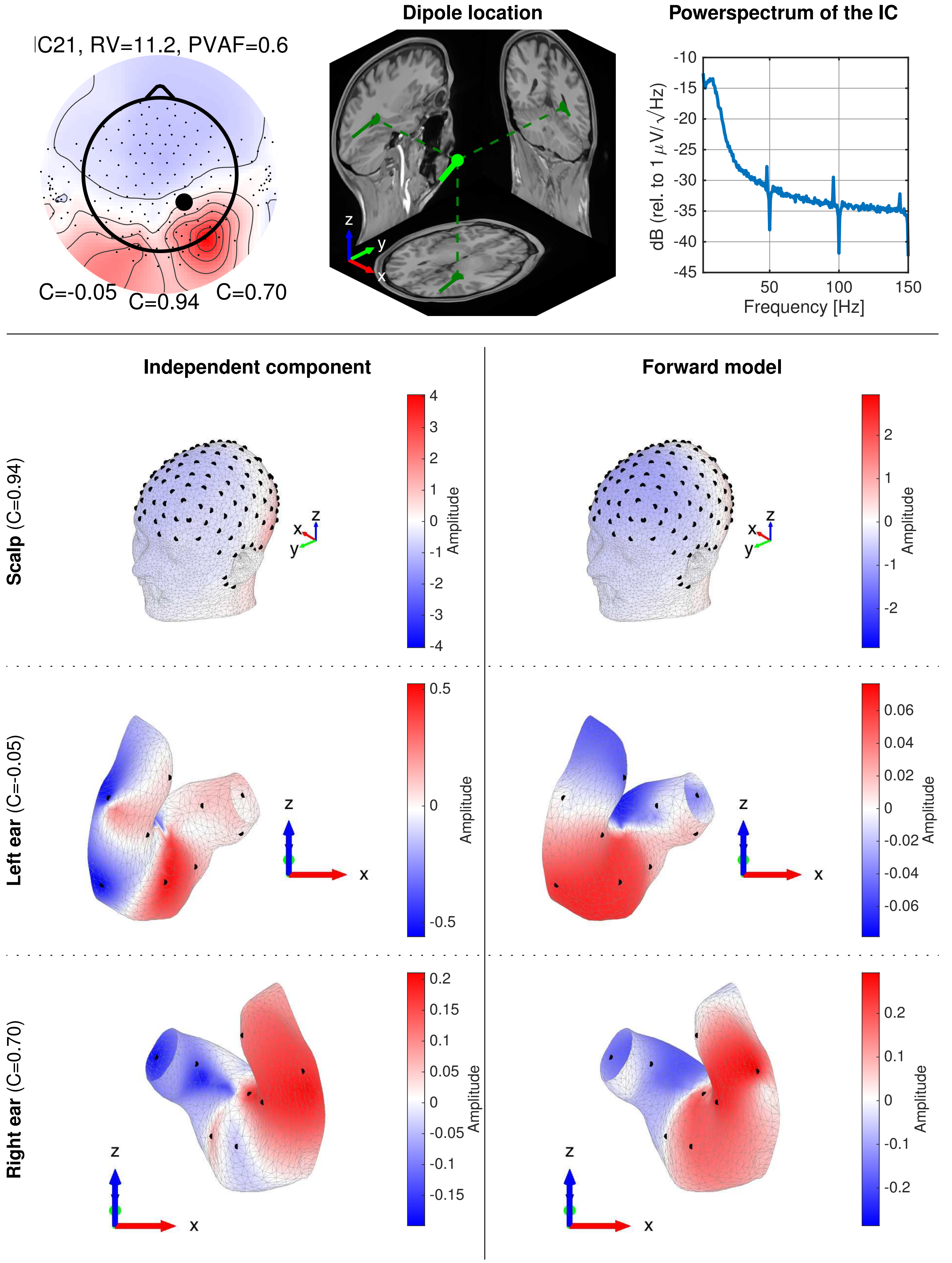

Subject D - IC 24

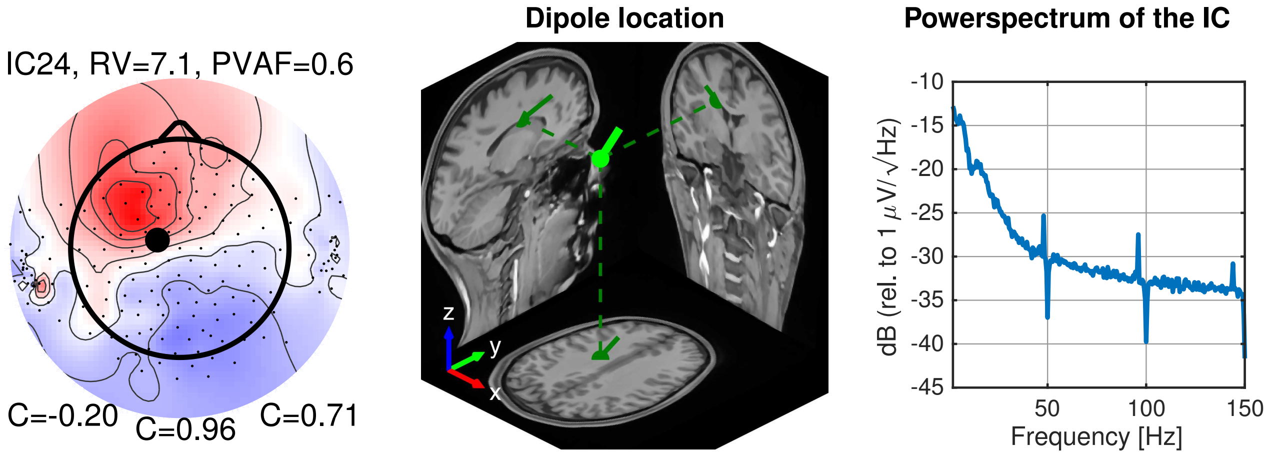

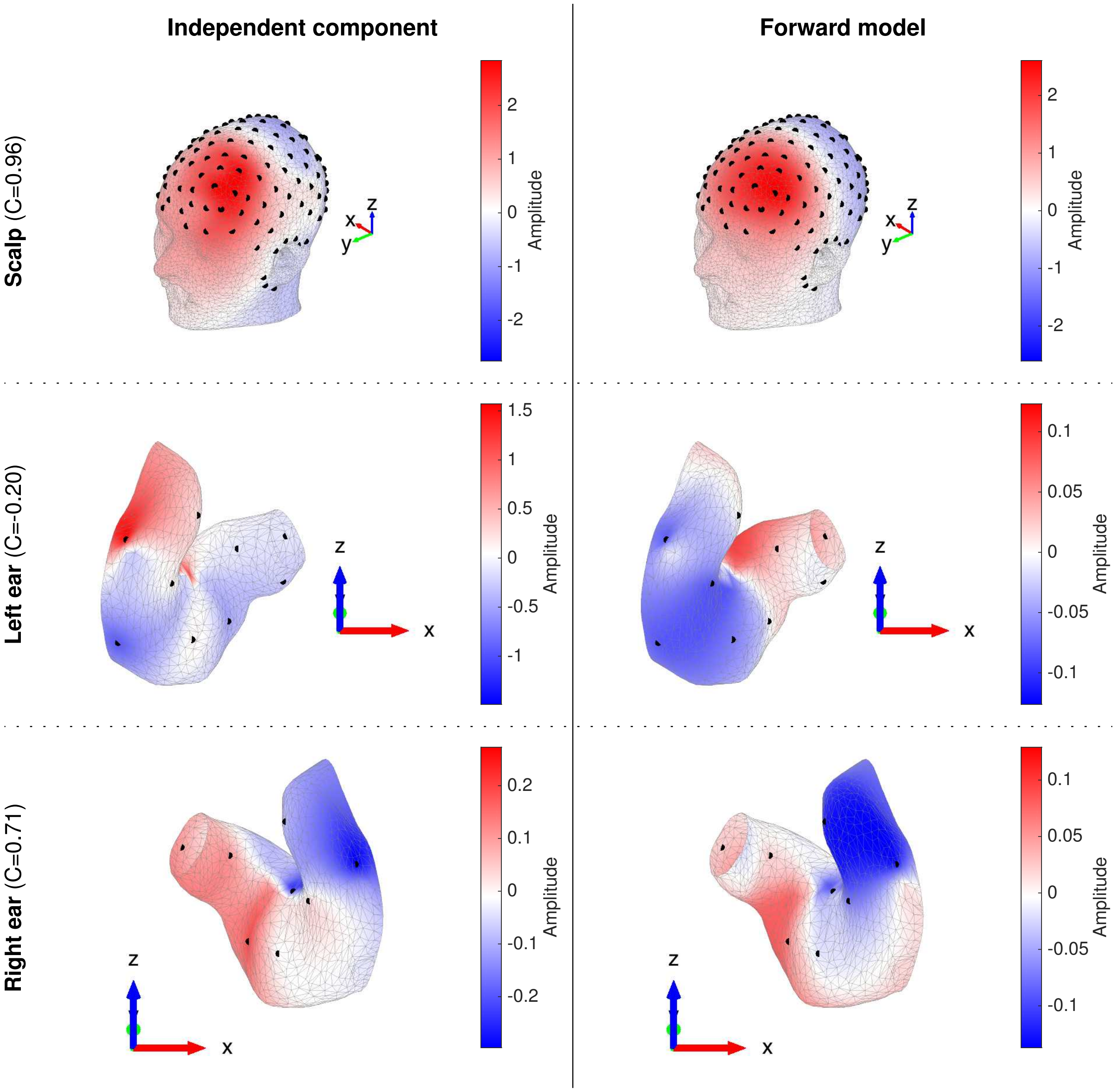

Subject D - IC 38

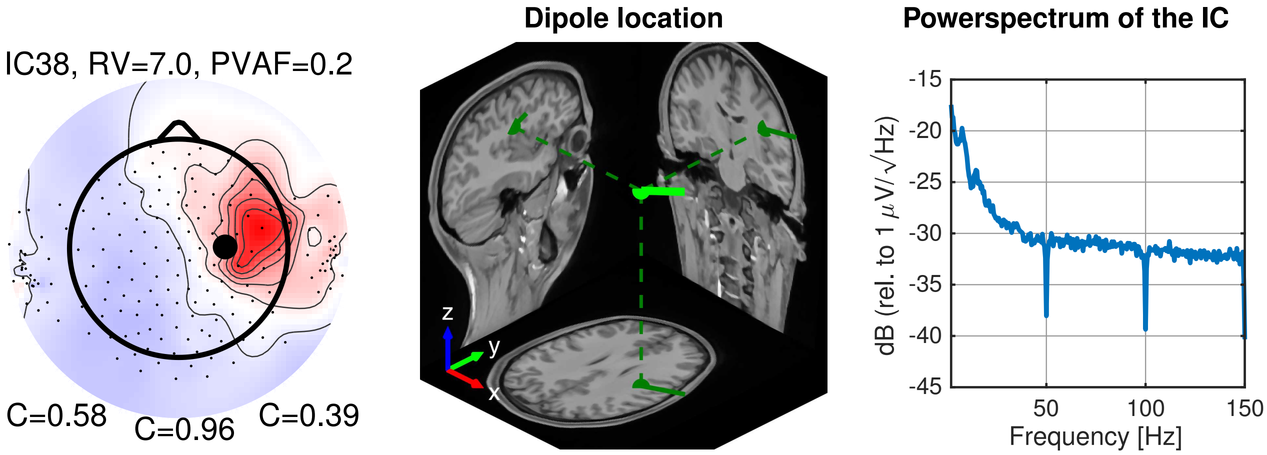

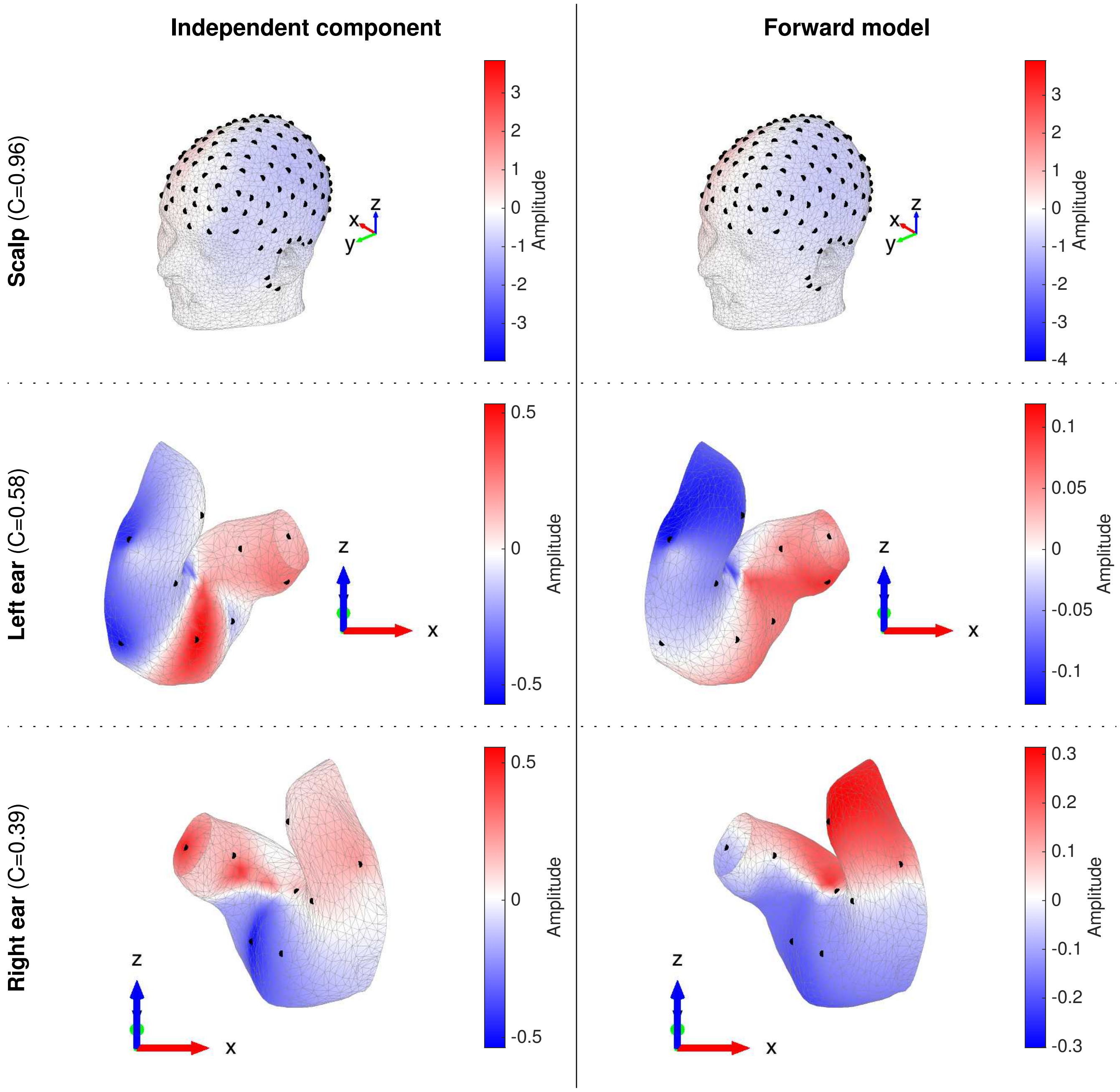

Supplement: Supplementary file 1 [file Data_Sheet_1.pdf]
